# A survey of highly cited studies on plant pathogen effectors during the last two decades (2000-2020)

**DOI:** 10.3389/fpls.2022.920281

**Published:** 2022-12-05

**Authors:** Clémentine Louet, Sébastien Duplessis, Pascal Frey, Benjamin Petre

**Affiliations:** Université de Lorraine, INRAE, IAM, Nancy, France

**Keywords:** bibliometric pipeline, web of science, plant immunity, avirulence, virulence, pathogenicity, receptor

## Abstract

Plant effector biology is a research area that describes how plant-associated organisms modulate host structures and function to promote colonization by using small molecules (effectors). In this article, we analyzed 249 highly cited publications focused on plant pathogen effectors (*i.e.*, Highly Influential studies on plant Pathogen Effectors; thereafter HIPEs) published between 2000 and 2020. This analysis identifies countries, organizations, and journals that contributed HIPEs, and reveals the evolution of research trends, model molecules, and model organisms over the last two decades. We notably show an increasing proportion of studies focused on effectors of biotrophic and hemibiotrophic fungi upon time. Our snapshot of the highly influential plant effector biology papers may help new comers in the field to gain an analytical understanding of this research area.

## Introduction

Plant pathogens threaten agricultural production by triggering dramatic yield losses worldwide (Savary et al., 2019). The effector biology research field explains how pathogens manipulate their host(s) to promote infection through the modulation of host structures and processes, and aims at leveraging that knowledge to ultimately improve plant health (Win et al., 2012). Since the 2000s, this field of research grew spectacularly, with a steady increase in the number of publications (https://www.webofscience.com; **Supplementary Figure S1**). During these two decades, our understanding of effector diversity and functions has improved, notably thanks to functional genomic studies. Few recent studies inventoried the major findings and concepts of plant effector biology (Win et al., 2012; Toruño et al., 2016), which makes it difficult for junior researchers and new comers in the field to gain a broad and analytical understanding of the research area.

To build a snapshot of top cited research in plant effector biology, we implemented the ‘HIP in’ (‘Highly Influential Publication in’…) method that we recently described (Petre et al., 2022). We report here the bibliometric analysis of 249 Highly Influential (*i.e.* highly cited) papers on plant Pathogen Effectors (thereafter HIPEs) published between 2000 and 2020. We describe the concepts, model objects, and findings shared by HIPEs, as well as the research community that shared them. We notably show the increasing importance of fungi as model pathogens over time, and highlight key fungal species.

## A bibliometric pipeline identifies 249 highly cited publications addressing plant pathogen effectors

**Figure 1:**
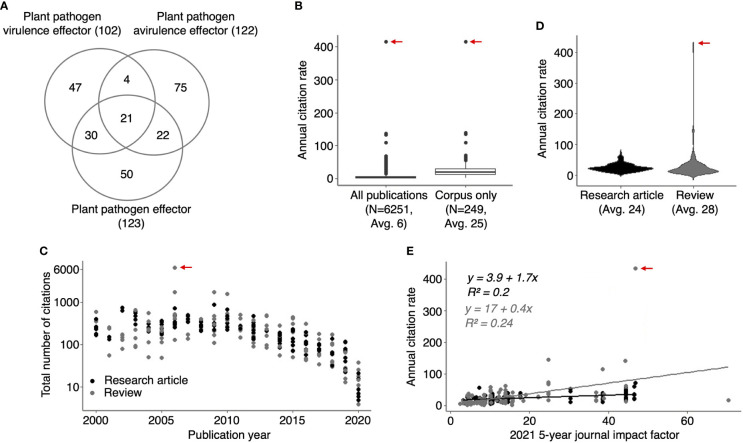
HIPEs are highly cited studies addressing plant pathogen effectors. **(A)** Venn diagram indicating the number of overlapping publications between the three groups of keywords (‘plant pathogen effectors’, ‘plant pathogen avirulence effectors’ and ‘plant pathogen virulence effectors’) used to build the HIPE collection. The number of publications is shown between parentheses. **(B)** Boxplots displaying the annual citation rate (*i.e*., average number of citations per year) of all publications identified with the keyword searches (left-hand side; ‘All publications’) and the HIPEs only (right-hand side; ‘Corpus only’). The boxplot outliers are indicated as dots. The number of publications and the average of annual citation rate for publications considered is indicated below each boxplot (N and Avg value, respectively). **(C)** Scatterplot displaying the number of total citations of individual HIPEs according to their publication year. The total number of citation value was extracted from Web of Science portal (accessed in July 2021). **(D)** Violin plots displaying the annual citation rate of HIPE research articles and reviews. The average of annual citation rate for publications considered is indicated below each violin plot (Avg value). **(E)** Scatterplot displaying the annual citation rate of individual HIPEs according to the value of the 2021 5-year journal impact factor (‘current five-years journal impact factor’ on the Web Of Science portal) of the journal in which the HIPEs were published. Black and grey dots indicate research articles and reviews, respectively. Linear trendlines are indicated for each article type along with trendline equations and r-squared values, with the same color-code as previously. In **(B-E)** the red arrow indicates the HIPE with the highest annual citation rate, which corresponds to the 2006 review by Jones and Dangl that presented the zig-zag model of the plant immune system. The raw data used to build this figure are available in the **Dataset 1**.

To identify the most cited publications that addressed plant pathogen effectors over the last two decades, we implemented a previously described bibliometric pipeline (Petre et al., 2022), which mostly uses the Web of Science database (https://www.webofscience.com) and a reference management software (here Mendeley). We selected the top three most cited research articles or reviews published each year between 2000 and 2020, which we identified *via* three successive searches using the key terms ‘plant pathogen effectors’, ‘plant pathogen avirulent effectors’, and ‘plant pathogen virulent effectors’ (**Figure 1A**; **Dataset 1**; see **Supplementary Methods**, **Supplementary Figure S2** for details). In total, we collected and archived 249 HIPEs (127 research articles and 122 reviews; hereafter the ‘HIPE collection’) on the following public web address: https://www.zotero.org/groups/4410902/hipe_collection/library.

The HIPEs represent nearly 4% of all the publications identified *via* the three searches mentioned above (249 out of 6251), but their annual citation rate is approximately four times higher (25 vs. 6 on average; **Figure 1B**). The HIPE with the highest number of citations (6503) and annual citation rate (414) is the review that presented the seminal zig-zag model (Jones and Dangl, 2006). The total number of citations received by a given HIPE unsurprisingly correlates with its publication year (*i.e.* HIPES published between 2000 and 2010 received on average 404 citations, ranging from 49 to 6503, while HIPEs published in 2020 received on average 13 ± 7 citations; **Figure 1C**). Interestingly, the annual citation rates of research articles and reviews are comparable (24 vs. 28 on average; **Figure 1D**). In addition, the current impact factors of the journals in which HIPEs were published do not correlate with HIPEs annual citation rate (**Figure 1E**). Thus, publication types and journal impact factors poorly explain the variation of citation rates between HIPEs. As a note, only 19 out of 249 HIPEs overlap with the collection of highly-cited papers in plant immunity (HIPPYs) described in our previous bibliometric analysis (Petre et al., 2022; **Dataset 1**, column B). To summarize, HIPEs represent an original collection of influential studies that pertains to plant pathogen effectors, and that is suitable for further analyses.

## A small influential community publishes the majority of the HIPEs

To identify the research community that publishes the HIPEs, we extracted from HIPEs metadata the main countries and institutions to which HIPEs corresponding authors are affiliated, as well as the journals that published them. Overall, 19 countries, 87 institutions (comprising 154 affiliated corresponding authors), and 67 journals published the HIPEs (**Dataset 1**). Only a handful of scientific actors contributed most of the collection (**Table 1**). Three countries (USA, UK, and Germany) published 62% of the HIPEs, whereas four institutions published a quarter of the HIPEs: the Max Planck Institute (MPI, Germany), The Sainsbury Laboratory (TSL, UK), the French National Research Institute for Agriculture, Food, and Environment (INRAE, France), and Wageningen University & Research (WUR, Netherlands). Also, four journals (Science, Annual Review of Phytopathology, PNAS, and The Plant Cell) contributed over a quarter of the HIPEs. Altogether, this analysis indicates that HIPEs originate from a restricted number of academic actors mostly based in Western Europe and in the USA.

**Table 1:** Top countries, institutions, and journals that publish the most HIPEs.

Country, institution, or journal^a^	Number of HIPEs	Number of citations	Number of corresponding authors	2021 5-year journal impact factor
USA	88	25,167	52	–
UK	35	13,939	25	–
Germany	31	7,581	19	–
INRAE (France)	18	3,099	13	–
TSL (UK)	15	10,082	7	–
MPI (Germany)	15	4,034	10	–
WUR (Netherlands)	14	3,190	6	–
Science	20	7,013	–	44.37
Annual Review of Phytopathology	19	4,925	–	13.87
PNAS	17	4,702	–	10.62
The Plant Cell	17	3,686	–	10.14

a USA, United States of America; UK, United Kingdom; INRAE, the French National Research Institute for Agriculture, Food, and Environment; TSL, The Sainsbury Laboratory; MPI, Max Planck Institute; WUR, Wageningen University & Research; PNAS, Proceedings of the National Academy of Science of the USA.

## HIPEs collectively address seven main research questions, whose relative importance within the HIPE collection evolved over time

To identify the main research questions addressed by the HIPEs, we performed an iterative analytical reading combined with keyword tagging of all the collection aimed at identifying HIPEs main research topics and sub-topics. This analysis identified seven main research topics, as follow: 1) the ‘PTI’ (PAMP-triggered immunity; *what is the interplay between PTI and effectors?*; 10 HIPEs), 2) the ‘ETI’ (Effector-triggered immunity*; what is the interplay between ETI and effectors?*; 41 HIPEs), 3) the ‘ETS’ (Effector-triggered susceptibility; *how do effectors modulate host functions?*; 78 HIPEs), 4) the ‘Effector trafficking’ topic (*how do pathogens deliver effectors to the host?*; 16 HIPEs), 5) the ‘Pathogen highlight’ topic (*what is the current knowledge in the field for specific pathogen species?*; 12 HIPEs), 6) the ‘Pathoresources’ topic (*how to build and leverage technological innovation and resources to better understand plant-pathogen interactions?*; 53 HIPEs), and 7) the ‘General review’ topic (*how do we conceptually understand the role of effectors in plant-pathogen interactions?*; 39 HIPEs) (see **Supplementary Text** for details). Interestingly, these seven main research topics present comparable annual citation rates (**Supplementary Figure S3**).

**Figure 2:**
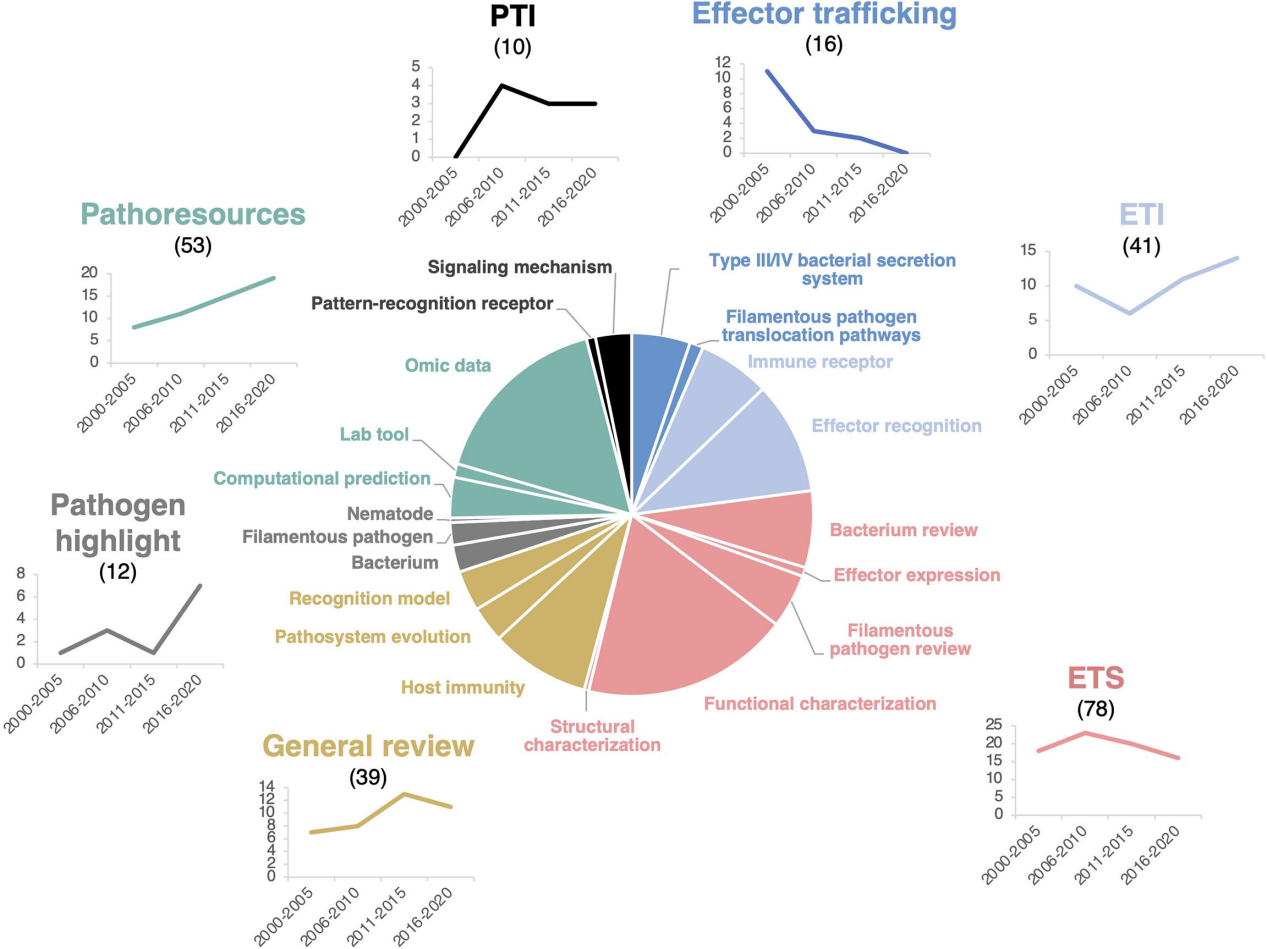
HIPEs pertain to seven main research topics. Pie chart displaying the seven main research topics and 20 sub-topics addressed by the HIPEs and their publication trends over time. The main research topics, sub-topics, and trendlines associated are color-coded as indicated in **Figure S3**. Sub-topics are indicated as separated pie segments within a main topic. The categorization of each HIPE was based on iterative expert reading and keyword association of the entire HIPE collection. Numbers above the main research topics indicate the total number of HIPEs for each topic. Trendlines indicate the number of HIPEs for each topic according to one six-year time frame (low number of publications during this frame) and four five-year time frames: 2000-2005, 2006-2010, 2011-2015, and 2016-2020, respectively. PTI: Pattern-triggered immunity; ETI: Effector-triggered immunity; ETS: Effector-triggered susceptibility. The percentage of reviews within each topics is as follow: effector trafficking (63%), ETI (27%), ETS (42%), general reviews (100%), pathogen highlight (100%), pathoresources (21%), and PTI (60%). The raw data used to build this figure are available in the **Dataset 1**.

To gain a more accurate understanding of HIPEs research questions, we further grouped HIPEs into 20 sub-topics so that each of the seven main research topics comprises two to five sub-topics that pertain to a more specific research question (**Figure 2**). For example, the ETI topic comprises two sub-topics: ‘Immune receptors’ (*what are the receptors that recognize effectors*?) and ‘Effector recognition’ (*how do immune receptors recognize effectors and signal that recognition*?). Overall, the 20 sub-topics highlight several specific research questions; the most addressed being ‘what is the function of pathogenic effectors in the host?’ (18% of HIPEs, ETS topic) and ‘how do we develop and use omics data to identify pathogenic effectors?’ (16% of HIPEs, Pathoresources topic).

To identify potential research trends in plant effector biology, we analyzed the evolution of the number of HIPEs in each of the seven topics between 2000 and 2020, by one six-year time frame (*i.e.* 2000-2005; low number of publications during this frame) and three five-year time frames (*i.e.* 2006-2010, 2011-2015, and 2016-2020). That analysis showed that five topics remain stable over time, while two topics - Effector trafficking and Pathoresources - showed marked trends of decrease and increase over time, respectively. The Effector trafficking topic declined markedly over the years, mainly due to the reduction of HIPEs addressing the bacterial Type III or Type IV secretion system. The controversy that persisted throughout the 2010s about the mechanisms of delivery of filamentous pathogen effectors may also explain the topic decline (Tyler et al., 2013; Wawra et al., 2013; Anderson et al., 2015; Wawra et al., 2017). At the opposite, the Pathoresources topic steadily increased, probably benefiting from the cost-effective progresses in genomics that assisted the massive identification and testing of candidate effectors in a diversity of pathogens (**Figure 2**).

**Figure 3:**
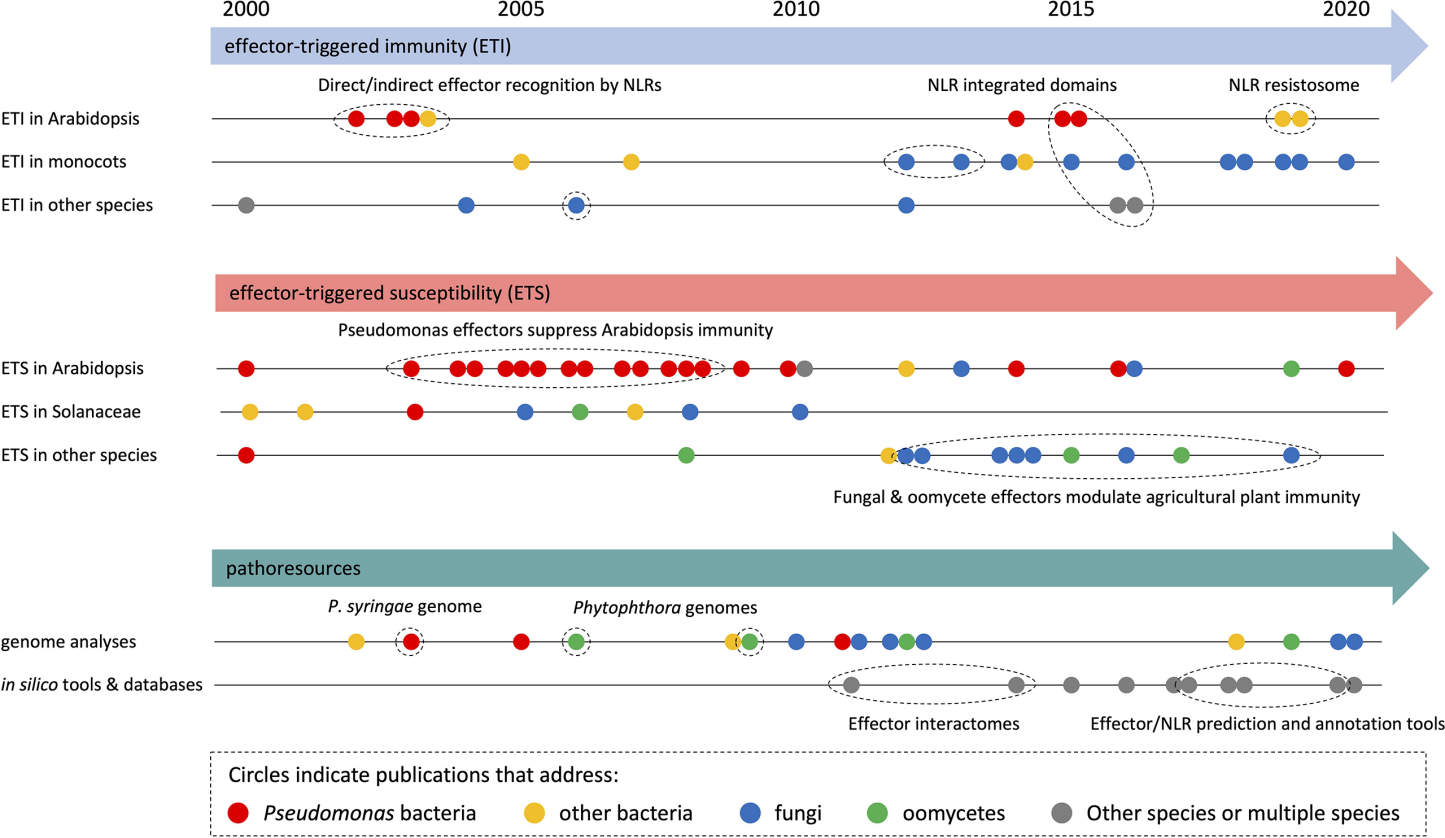
HIPEs model objects shifted within the early 2010s. Analytical timeline of selected HIPEs from the three largest categories: effector-triggered immunity (ETI; top panel), effector-triggered susceptibility (ETS; intermediate panel), and pathoresources (bottom panel). HIPEs are shown as dots, which are: i) colored according to the model organism that they mainly address (see legend on top), ii) positioned onto various timeline (see on the left) according to their content, and iii) positioned horizontally according to their publication date. Details are shown for some HIPEs (of coherent groups of HIPEs). Dotted ellipses highlight key HIPEs (or groups of HIPEs) that reported coherent discoveries or addressed linked research questions. The raw data used to build this figure are shown in **Dataset 1**.

To analyze research trends in more detail, we built timelines with selected HIPEs from the three largest categories (*i.e.*, ‘ETI’, ‘ETS’, and ‘pathoresources’, which account for three quarters of the HIPE collection) (**Figure 3**). Overall, this analysis revealed that HIPEs focus shifted over time, with a tipping point around the early 2010s. Firstly, for all three categories, HIPEs addressing bacteria (mostly *Pseudomonas* spp.) and Arabidopsis predominate between 2000 and 2010; in contrast, HIPEs addressing filamentous pathogens (fungi and oomycetes) and other plants species (notably monocots such as wheat, barley, and rice) become predominant after 2010. Secondly, regarding the ETI category, most HIPEs focused on NLR function, by first addressing the direct or indirect nature of effector recognition in the 2000s, then the identification and characterization of integrated domains present in NLRs in the mid-2010s, and finally the structural re-arrangements associated with effector-mediated NLR activation in 2019. Thirdly, regarding the ETS category, nearly all HIPEs published in the 2000s addressed how bacterial effectors suppress immunity in Arabidopsis or Solanaceae; after 2010, over 70% of the HIPEs addressed fungal or oomycete effectors, mostly in species different from Arabidopsis or Solanaceae. Finally, regarding the pathoresources category, genome analyses shifted focus from bacteria to filamentous pathogens in the early 2010s. By mid 2010s, the growing number of genomic and post-genomic studies permitted the generation of a series of freely accessible online databases and resources, for instance to predict effectors in filamentous pathogens.

In conclusion, this set of analyses shows that HIPEs pertain to key research questions that address well-defined aspects of the molecular interaction between plants and pathogens; the relative importance of some of those increased (*i.e.* Pathoresources) or declined (*i.e.* Effector trafficking) over the last two decades. Also, the number of HIPEs addressing filamentous pathogens, agricultural species (notably monocots such as wheat, barley, and rice), and the development of online resources increased markedly in the early 2010s.

## HIPEs address a handful of key organisms and proteins

**Figure 4:**
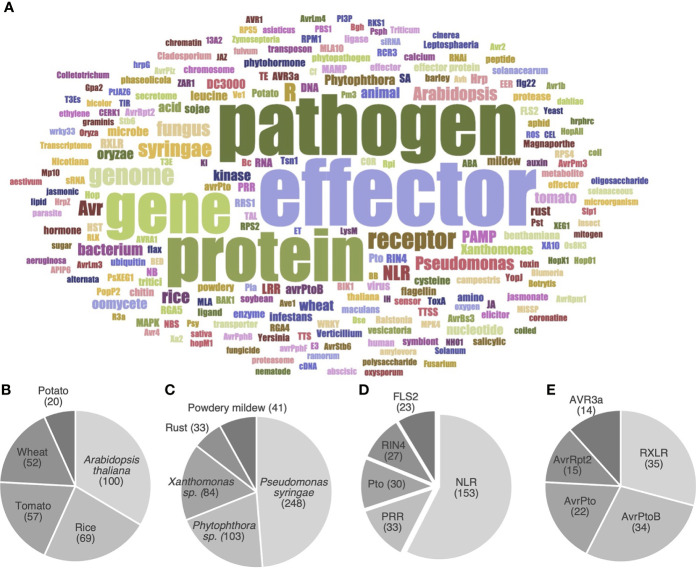
HIPEs mainly address a handful of model organisms and proteins. **(A)** Word cloud displaying the most frequent words referring to organisms or molecules in the title and abstract of the HIPEs. The word cloud was built using a filtered text file including HIPEs metadata (publication title and abstract) deprived from words that did not explicitly refer to organisms or molecules. Word colors were randomly generated to assist visual word discrimination. The word cloud displays 200 words. The size of the words positively correlates with their frequency in the text file. A top five of plant organisms **(B)**, plant-associated pathogens **(C)**, immune receptors **(D)** and pathogen effectors **(E)** was identified based on **Dataset 2**. The following specific keywords were considered for the following topics: *Arabidopsis thaliana* (*‘Arabidopsis’* and *‘thaliana’*), *Pseudomonas syringae* (*‘Pseudomonas’*, *‘syringae’*, ‘DC3000’ and ‘pst’), *Phytophthora* sp. (*‘Phytophthora’*, *‘infestans’*, *‘ramorum’* and *‘sojae’*), *Xanthomonas* sp. (*‘Xanthomonas’*, *‘campestris’* and *‘oryzae’* if used in a meaningful context), Powdery mildews (‘Powdery’ and ‘mildews’), NLR (‘NLR’, ‘LRR’, ‘NB-LRR’, ‘TNL’, ‘CNL’, ‘sNLR’ and ‘hNLR’). NLR: nucleotide-binding leucine-rich-repeat receptor; PRR: pattern recognition receptors. The raw data used to build this figure are available in the **Dataset 2**.

To further identify the main objects (*i.e.* organisms or molecules) investigated by the HIPEs, we performed a word occurrence analysis of the title and abstracts combined with word cloud generation and iterative text enrichment for words referring to organisms or molecules (**Dataset 2**; see **Supplementary Methods** for details). That pipeline generated a world cloud of the 200 most frequently used words referring to organisms or molecules (**Figure 4A**). As expected, the most used words refer to key groups of organisms (pathogen, *Arabidopsis*, bacterium, fungus, *Pseudomonas*) or groups of molecules (effector, receptor, NLR, gene, protein, genome). We further quantitatively analyzed **Dataset 2** to identify the top five most frequently referred to plant species, pathogen species, immune receptors, and effectors (**Figures 4B-E**). Firstly, the top five model plants comprise a Brassicaceae (*Arabidopsis thaliana*), two Poaceae (*i.e.* cereals; rice and wheat), and two Solanaceae (potato and tomato). The fact that three out of these five model plants are key crops providing staple food worldwide (*i.e.* rice, wheat, and potato; Savary et al., 2019) suggests that fundamental research and agricultural efforts align to meet the challenge of global food security (**Figure 4B**).

Secondly, the top five model pathogens comprise two bacteria (*Pseudomonas syringae* and *Xanthomonas* spp.), two groups of fungi (Pucciniales and Erysiphales, causing rust and powdery mildew diseases, respectively), and one genus of oomycetes (*Phytophthora* spp.) (**Figure 4C**). These pathogens all cause a wide range of diseases that significantly impact agricultural production worldwide (Savary et al., 2019; Fones et al., 2020). Thirdly, the top five model receptors comprise two general groups of immune receptors (NLRs and PRRs), as well as three specific proteins: FLS2 (the PRR that recognizes flagellin), Pto (a cytosolic kinase), and RIN4 (an immune regulator). The prominence of NLRs mirrors their importance in ETI, and the importance of ETI as a research topic (**Figure 4D**). Finally, the top five model groups of effectors comprise three *P. syringae* effectors (AvrPto, AvrPtoB, and AvrRpt2) and two oomycete effector families (the RXLR superfamily and the AVR3a family) (**Figure 4E**). Altogether, these analyses reveal the prominence of *A. thaliana*, *P. syringae*, as well as receptor and effector gene families as model objects in effector biology. We refer readers to the **Supplementary Text** for a more detailed and contextualized analysis of the organisms and molecules listed above.

## Temporal analysis of model pathogens reveals the increasing importance of plant pathogenic fungi

**Figure 5:**
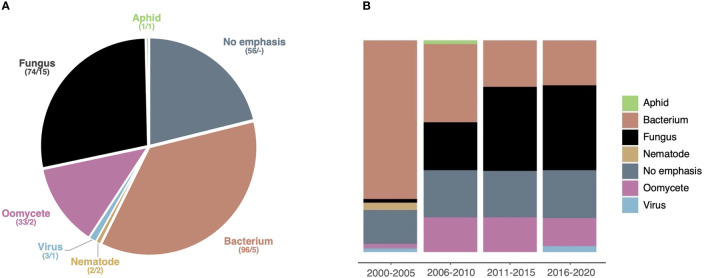
Studies emphasizing fungi gain importance over time. **(A)** Pie chart displaying the seven taxonomic groups of pathogens emphasized (if any) addressed by the HIPEs. The assignment of each HIPE was based on iterative expert reading of the publications. For each taxonomic group, the number of HIPEs and the total number of species are indicated (*e.g*. ‘74/15’). **(B)** Percent stacked bar chart displaying the evolution of the relative size of taxonomic groups over one six-year time frame (low number of publications during this frame) and four five-year time: 2000-2005, 2006-2010, 2011-2015, and 2016-2020, respectively. Color code is the same as in **(A)**. The raw data used to build this figure are available in the **Dataset 1**.

To get a more detailed understanding of the model pathogens addressed in HIPEs, we performed an iterative analytical reading of all HIPEs and categorized them according to the taxonomic group of the pathogens they emphasized (if any). This analysis revealed that 80% of the HIPEs emphasize a specific pathogen; we grouped those HIPEs into six categories: bacteria (96 HIPEs), fungi (74), oomycetes (33), viruses (3), nematodes (2), and aphids (1) (**Dataset 1**; **Figure 5A**). Only a handful of HIPEs emphasize aphids, viruses and nematodes, suggesting that the effector biology community revolves mostly around large communities studying bacterial, fungal and oomycete model systems. Notably, the categories ‘bacteria’ and ‘oomycetes’ (33% and 13% of the collection, respectively) show a low model diversity, as they mostly address *Pseudomonas* spp. and *Phytophthora* spp., respectively (**Dataset 1**). In contrast, the fungal group (28% of the HIPEs) shows a high model diversity, as it addresses 15 different fungal genera (**Dataset 1**). Interestingly, the size of the categories evolved drastically between 2000 and 2020 (**Figure 5B**). Indeed, between the 2000-2005 and the 2016-2020 time periods, studies focusing on bacteria declined from 75% to 21% of the HIPE collection, while studies focusing on oomycetes and fungi increased from 2% to 13% and from 2% to 40%, respectively. These results indicate that in the early 2000s model bacterial systems vastly dominated effector biology, but that over time filamentous pathogens (*i.e.* fungi and oomycetes) gained importance. This likely reflects the impact of pathogenomics and the possibility for systematic effectors prediction in sequenced genomes of a larger number of filamentous microbes such as fungi.

## The influence of publications addressing fungal effectors increased over time: analysis of the ‘HIPE-fun’ collection

To perform an objective analysis of the most prominent fungal species in highly cited publications addressing fungal plant pathogen effectors, we repeated the bibliometric analysis that identified the HIPE collection, by considering only the research articles addressing fungi. This analysis helped to build a collection of 100 HIPEs focusing on fungi (thereafter referred to as ‘HIPE-funs’; **Dataset 3**; see **Supplementary Methods**, **Supplementary Figure S2** for details), archived on the following public web address: https://www.zotero.org/groups/4410905/hipe-fun_collection/library.

**Figure 6:**
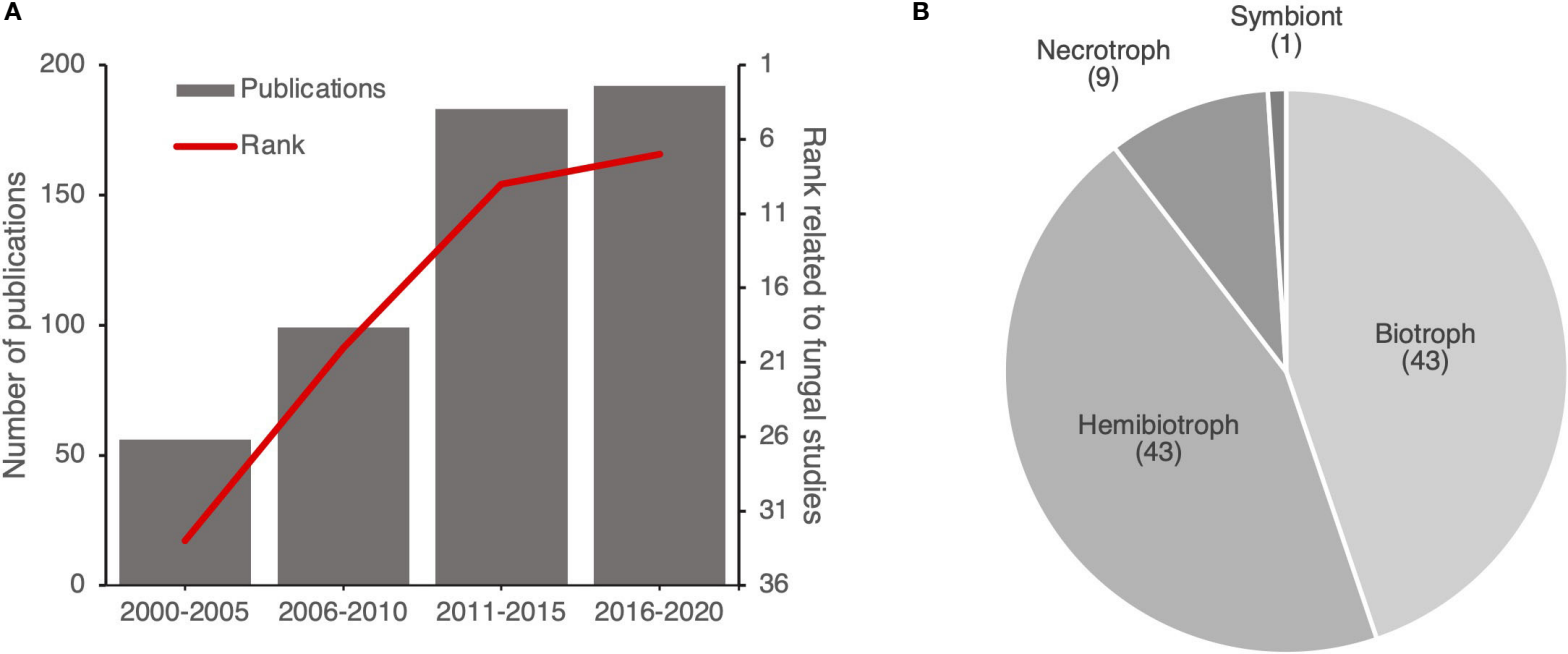
Publications addressing effectors of (hemi)biotrophic fungi gain prominence over time. **(A)** Evolution of the number of publications related to plant pathogen effectors and the citation rank for HIPE-funs. Each citation rank was calculated based on the mean of rank of publications by time frames (*i.e.* position of the publication on the Web of Science website based on the number of citations). **(B)** Pie chart displaying the proportion of each lifestyle of plant pathogenic fungi studied in the HIPE-funs. The categorization of each lifestyle was based on iterative expert reading and association with the pathogenic species studied for the entire HIPE-fun collection. Numbers above lifestyle topics indicate the total number of HIPE-funs for each topic. The raw data used to build this figure are available in the **Dataset 3**.

To understand the increase of the number of publications focused on fungi within HIPEs, we extracted the citation rank over years of the HIPE-funs (*i.e.* rank of the publication on the Web of Science database based on the number of citations) and the number of publications addressing plant pathogen effectors. This analysis revealed an obvious increase over the years of the citation rank of the HIPE-funs, which coincided with the increasing number of publications addressing plant pathogen effectors in general. Indeed, in 2000-2005 fungal studies ranked 33rd out of 56 publications, while in 2016-2020 they ranked 7th out of 192 publications (**Figure 6A**). Thus, publications addressing fungi drastically gained visibility over time. Interestingly, most of those studies (90%) focused on either biotrophic or hemibiotrophic fungi (**Figure 6B**).

## The HIPE-funs appertain to influential fungal species in the field of molecular plant pathology

In 2012, Dean and colleagues surveyed hundreds of mycologists who voted for the most important fungal species in molecular plant pathology based on their scientific and economic importance (495 votes in total). We performed a comparative analysis to evaluate the correspondence between the fungal species addressed in HIPE-funs and the top 10 list from Dean et al. (2012). We first identified a total of 21 fungal genera covered in the HIPE-funs. We ranked fungal genera according to HIPE-funs numbers and annual citation rates, and then we compared this ranking with the list by Dean and colleagues (**Table 2**). First, this analysis showed that all top 10 species occur in at least one HIPE-fun (**Dataset 3**). Notably, the genera *Magnaporthe* and *Puccinia* appear in the top of both lists (**Table 2**; Dean et al., 2012). These genera comprise species that trigger dramatic epidemics on rice and/or wheat, threatening global food security (Talbot, 2003; Wilson and Talbot, 2009; Figueroa et al., 2018). That may explain why they represent important fungal models in the field of fungal effector biology. In contrast, the ranking of some species differs between the two lists. For instance, the genus *Botrytis* dominates the top 10 list of Dean et al. (2012), whereas it is barely represented in the HIPE-funs (**Table 2**). This genus, and notably the species *Botrytis cinerea*, is a strong model for molecular plant pathology but not in effector biology. This is probably because of its necrotrophic lifestyle (poorly represented within the HIPE-funs; **Figure 6B**); a lifestyle that is thought not to rely much on effectors for host infection, but rather on toxins. To conclude, our analysis of HIPE-funs confirms the importance of well-established fungal pathogens as agronomical models in both effector biology and molecular plant pathology.

**Table 2:** Most occurring fungal genera within the HIPE-funs.

Fungal genera	Rank	Number of HIPE-funs	Annual citation rate	Rank based on Dean et al., 2012 top 10 list
*Blumeria* sp.	1	12	240	6
*Magnaporthe* sp.	2	10	206	1
*Leptosphaeria* sp.	3	10	136	–
*Cladosporium* sp.	4	9	171	–
*Puccinia* sp.	5	9	73	3
*Verticillium* sp.	6	6	163	–
*Melampsora* sp.	7	6	121	10
*Zymoseptoria* sp.	8	6	117	7
*Fusarium* sp.	9	6	84	4 & 5
*Colletotrichum* sp.	10	4	124	8
*Ustilago* sp.	11	4	87	9
*Sclerotinia* sp.	12	4	67	–
*Botrytis* sp.	13	2	117	2

## Conclusion and outlook

In the present study, we showed that publications highly cited in plant effector biology have developed as a well-structured research area, with key academic actors and model objects. The study also reveals that the focus of the most cited publications in the field has partially shifted over time, from bacterial pathogens to filamentous pathogens (notably fungi). As an outlook for the next twenty years, we anticipate that the research area will continue to diversify the organisms it addresses (*i.e.* aphids, herbivores, viruses), which may help understand better the diversity of virulence strategies that arose throughout evolution. In addition, we prompt readers who want to gain a deeper understanding of the field and develop creative research thinking to diversify their sources of information by exploring the literature beyond the HIPEs. Notably, we encourage readers who want to update their knowledge and gain fresh perspectives on the topics developed in this study to consult recent special issues (Jones and Dangl, 2021; Innes et al., 2022) as well as excellent comprehensive reviews addressing plant pathogen effectors and molecular plant immunity in general (Lo Presti et al., 2015; Xin et al., 2018; Harris et al., 2020; Mukhi et al., 2020; Ngou et al., 2022).

As a concluding and cautionary note, although our approach may facilitate the discovery of the field of plant effector biology by early-career researchers and newcomers, it neither claims to be exhaustive nor sufficient to assist the development of an expert knowledge of the field. For instance, keywords searches are not infallible; the terms we used to identify HIPEs have missed some seminal and highly cited publications. For instance, our searches missed the key publications that reported the TAL effector code or clusters of virulence effectors in fungal pathogens, because the metadata of the publications (*i.e.*, the title, the abstract, and the keyword list) did not use the key terms ‘pathogen’ and ‘effector’, respectively (Kämper et al., 2006; Boch et al., 2009). This illustrates well the necessity for readers to diversify the search terms while exploring publication databases. It also prompts authors and editors to carefully select keywords that appear in the metadata in order to maximize the discoverability of their study and its visibility among the targeted readership.

## Author contributions

CL acquired data and wrote the manuscript. CL performed data analysis and interpretation, with inputs from BP and SD. All authors conceived and designed the study, as well as critically revised and edited the manuscript. All authors contributed to the article and approved the submitted version.

## Funding

The authors are supported by the Pôle Scientifique A2F of the Université de Lorraine and by the French “Investissement d’Avenir” program ANR-11-LABX-0002-01, Lab of Excellence ARBRE. CL was supported by a PhD fellowship from the Région Lorraine and the French National Research Agency (ANR-18-CE32-0001, Clonix2D project).

## Acknowledgments

We acknowledge M. Saubin, F. Lauve-Zannini, and the members of the UMR IAM for fruitful discussions and continuous support throughout the course of this project. The authors also acknowledge members of the community met over the past ten years at the different meetings of the Effectome network (supported by the INRAE scientific divisions SPE and EFPA) for stimulating discussions on the topic of plant pathogen effectors.

## Conflict of interest

The authors declare that the research was conducted in the absence of any commercial or financial relationships that could be construed as a potential conflict of interest.

## Publisher’s note

All claims expressed in this article are solely those of the authors and do not necessarily represent those of their affiliated organizations, or those of the publisher, the editors and the reviewers. Any product that may be evaluated in this article, or claim that may be made by its manufacturer, is not guaranteed or endorsed by the publisher.
