# Supplementary material for: A survey of highly cited studies on plant pathogen effectors during the last two decades (2000-2020)
Source: Front Plant Sci. 2022 Dec 5;13:920281. doi: 10.3389/fpls.2022.920281 (PMC9762492; doi:10.3389/fpls.2022.920281)
Supplement: Supplementary file 1 [file DataSheet_1.zip › Data Sheet 1 (17)/Dataset 2.docx]

A effector as a a bacterium on injection effector protein by into where they to pathogen such effector protein injected by Xanthomonas into is AvrBs3 which to hypertrophy mesophyll We that AvrBs3 a master upa20 which a a basic loop AvrBs3 to a upa20 promoter its gene through its AvrBs3 other this provoke developmental reprogramming by mimicking

A protein to have a to defend against by most pathogen such as Pseudomonas syringae have countermeasures protein into to intensive efforts protein that are for have Here we that a syringae protein HopM1 an protein AtMIN Arabidopsis thaliana HopM1 destruction AtMIN proteasome Our a by which a pathogen exploits proteasome to

A calmodulin gated calcium channel pathogen to pathogen PAMP both animal calcium has been as an for PAMP PAMP calcium unknown1 Here we that calcium status is for calcium PAMP When calcium supply is gene that cyclic nucleotide gated channel CNGC protein CNGC2 CNGC4 are for PAMP calcium Arabidopsis3 a reconstitution we that CNGC2 CNGC4 protein —but neither alone—assele into a calcium channel that is blocked by calmodulin resting Upon pathogen channel is by effector kinase BOTRYTIS KINASE1 BIK1 receptor this an concentration cytosolic calcium8 CNGC calcium a link between receptor calcium programs PAMP

A catalogue effector secretome oomycete oomycete a microorganism that some most notorious pathogen oomycete accomplish by modulating through an  effector protein effector is but tremendous has been This classifies catalogues  effector secretome oomycete effector   effector are into     effector are     where they Considering that are undergoing genome we are moving genome catalogues oomycete effector Already it is evident that  effector secretome oomycete is more than expected with perhaps hundred protein dedicated to manipulating

A Barley  pathogen  Pyrenophora as Drivers Pyrenophora net net barley is an pathogen is resources to a quality genome was generated for Here we quality we genome for an depth hypotheses pertaining to were performed between all genome examining organization variations on gene to effector protein We that trait QTL span with these being drivers Additionally these were frequently chromosome with QTL localizing to chromosome which was between nonhomologous chromosome at a higher than rest genome These that not only most but that these have to

A decade decoded spies hackers TAL effector effector TALEs from Xanthomonas are protein with to promoters gene to their which helps Metaphorically TALEs as spies that infiltrate disguised as ranking civilians to trick into activating weak that an how TALEs operate researchers to predict their counterespionage exploit their them to do our bidding a Manchurian This has been thanks to their DNA which obeys amino acid DNA correspondences TALE code Here we how researchers these protein what has changed ten code Recommended music for this can be Supplemental Material

A dual for pathogen effector protein protein from pathogen with have dual they on they as by eliciting reactions appropriate gene virus all protein by genome can be expected to be for have that are to most these protein bacterium fungus have gene latter were originally through gene elicitor their Their only became apparent when they were inactivated or transferred to microbe or after their microbe to maintain these gene their disadvantageous introducing only few to their with This has been interpreted as been indicative a gene that is not impaired by Alternatively bacterium there is that was acquired through gene gene supporting donor original to have traveled along Being gratuitous situation they have been inactivated any for pathogen

A effector salicylic acid necrosis Salicylic acid SA a coating pathogen Inactivation SA would be a a     pathogen It is that  effector  CEL     pathogen Pseudomonas syringae ACEL Erwinia amylovora dspA/ Pantoea stewartii subsp stewartii wtsE exert on their by We that DeltaCEL dspA/ was to their inability to to normal necrosis Arabidopsis apple DeltaCEL SA callose deposition Arabidopsis but failed to callose Arabidopsis blocked SA or This multiplied more aggressively SA deficient than hopPtoM avrE gene CEL syringae were to this SA conservation HopPtoM AvrE effector bacterium that SA promotion are for

A effector from Aphid Myzuspersicae Green Peach Aphid Aphids are amongst most sap feeding insect most parasites aphids require intimate associations with their to to Aphid feeding such as clogging phloem sieve callose formation which are by protein aphid saliva it is that aphids pathogen protein effector their to infestation We publicly aphid salivary gland tags ESTs to apply a for effector from Myzus persicae green peach aphid on pathogen effector A 48 effector were subjected to Nicotiana benthamiana to assay for a pathogen PAMP on aphid reproductive performance We effector Mp10 which chlorosis benthamiana conferred to recoinant Potato virus PVX Mp10 PVX Mp10 tabacum that this protein ubiquitin ligase protein SGT1 was for Mp10 chlorosis benthamiana Mp10 by flg22 but not by chitin Aphid fecundity that Mp10 Mp42 aphid fecundity effector MpC002 aphid fecundity these that Mp10 flg22 it a an decrease aphid performance fecundity we aphid salivary protein that with pathogen effector as aphid effector by perturbing

A game hide seek between gene AvrLm4 AvrLm3 Leptosphaeria maculans Extending durability gene pathogen is a We investigated between gene Leptosphaeria maculans AvrLm3 AvrLm4 When an possesses both gene Rlm3 oilseed rape Brassica napus is not to AvrLm4 but Rlm recover AvrLm3 Coining mapping RNA seq BAC artificial chromosome clone novo we AvrLm3 a telomeric gene maculans AvrLm3 is a gap maculans genome is surrounded by repeated for a cysteine protein is at stages Complementation masking AvrLm4 on AvrLm3 by Rlm3 we that AvrLm4 does not impede AvrLm3 Y2H between protein This unusual is for aiming to evaluate that Rlm durability

A near saturating for effector gene from Pseudomonas syringae Pseudomonas syringae nuers effector protein into during These protein are for because incapable delivering them are nonpathogenic We implemented a genome for syringae effector gene relied on FACS an arabinose inducible hrpL σ to automate HrpL gene We whether gene effector protein by creating testing protein fusions to a reporter Δ9AvrRpt2 that when fused to effector protein is elicits a Arabidopsis thaliana RPS2 protein Δ9AvrRpt2 is a marker for most criterion for defining effector protein We our collection effector protein from syringae This stringent 29 protein from syringae tomato from syringae phaseolicola Our hrpL effector suites from syringae that effector protein suites are this pathogen reflecting imposed by

A holistic on effector presented as an iceberg is It which is signaled manifested through branched multi To this pathogen effector steps This can cytosolic nucleotide

NLR receptor to effector pathogen have gene NLR effector only a few segregate as R gene Avr effector gene an attempt to explain this contradiction a is where most NLR effector effector keep a silent this so "iceberg " a few NLR effector coinations are visible above while is hidden below addressing existence NLR effector helps to explain why downregulation effector why lesion are Finally iceberg accommodates genuine as effector

A connecting pathogen pathogen Any given colonizes a outside this mount to attempted by this case pathogen same nucleotide leucine NB LRR protein receptor PRR Here we hypothesize that relative NB LRR PRR to as a time between Similarly pathogen expansions to be by pathogen effector to reproductive pathogen

A paralogous protects Phytophthora sojae effector PsXEG1 from a inhibitor a battleground between attacking microbe Here we that a pathogen xyloglucan endoglucanase PsXEG1 is a this struggle Phytophthora sojae soybean We that soybean an glucanase inhibitor protein GmGIP1 that to PsXEG1 to its to sojae a paralogous PsXEG1 protein PsXLP1 that has lost enzyme but to GmGIP1 more tightly than does PsXEG1 freeing PsXEG1 to sojae gene pair PsXEG1 PsXLP1 is Phytophthora parasitica orthologs PpXEG1 PpXLP1 have this be Phytophthora

A Phytophthora effector RNAi to RNA RNAi has a pathogen Phytophthora RNAi PSRs which enhance RNA against pathogen is unclear Here we that Phytophthora Arabidopsis to a pool interfering RNA siRNA Instead gene these siRNA are vesicles silence gene Phytophthora during Introduction a siRNA Phytophthora to developmental deficiency abolishes while Arabidopsis defective siRNA biogenesis are hypersusceptible Phytophthora effector PSR2 siRNA biogenesis Arabidopsis These uncover siRNA as agents against pathogen a /counter centered on gene between pathogen

A Phytophthora sojae Glycoside Hydrolase Protein Is a    during Soybean Is as a PAMP We a glycoside hydrolase GH12 protein XEG1 by soybean  pathogen  Phytophthora sojae that exhibits xyloglucanase beta glucanase It as an    during sojae but as a  pathogen PAMP soybean Glycine max solanaceous where it can GH12 protein taxa these GH12 protein Nicotiana benthamiana PAMP XEG1 is its xyloglucanase XEG1 can     a BAK1 XEG1 ethylene inducing xylanase XEG1 is sojae min soybean then slowly declines Both XEG1 sojae   sojae RXLR effector could by XEG1 that are min our that PsXEG1 to sojae  but soybean PsXEG1 to which can be by RXLR effector XEG1 an  effector that is   PAMP

A receptor detects pathogen effector that WRKY against pathogen multicellular eukary otes on receptor by these receptor is under stood nucleotide leucine NB LRR receptor fusions with other protein Arabidopsis RRS1 R NB LRR protein a WRKY DNA a receptor with RPS4 NB LRR protein This tects effector AvrRps4 or PopP2 then Both protein with RRS1 WRKY PopP2 acetylates lysines to DNA PopP2 AvrRps4 with other WRKY con taining protein these effector inter fere with WRKY fense RPS4/RRS1 has a ‘‘’’ that effector that WRKY protein We that NB LRR receptor onemeer which an pro tein pathogen effector whose is to that

A Pseudomonas syringae effector inactivates MAPK to PAMP pathogen PAMP pathogen have to these PAMP To bacterium Pseudomonas syringae effector protein which We that HopAll an effector both plantand animal pathogen Arabidopsis mitogen protein kinase MAPK by exposure to PAMP HopAll inactivates MAPK by removing phosphate from phosphothreonine through a phosphothreonine lyase which is for HopAll MAPK by HopAl namely reinforcement PAMP gene MAPK MPK3 MPK6 with HopAll that they are HopAll These uncover a by which Pseudomonas syringae overcomes to

A receptor pair with an converts pathogen disabling to pathogen infect by delivering effector that with fenses nucleotide / leucine receptor NLR effector by an Arabidopsis interacting NLR pair RRS1 R with RPS4 to pathogen Ralstonia solana cearum bacterium acetyltransferase effector PopP2 We that PopP2 acety lates a lysine an WRKY RRS1 R that DNA This disrupts RRS1 R DNA RPS4 PopP2 uses same lysine acetylation to promoting WRKY fac tors WRKY DNA activating needed for gene RRS1 R tegrates an effector with an NLR at DNA to a potent into gene

A receptor kinase BIK1 associates with a flagellin receptor to animal rely on to infections by microbe MAMP through receptor PRR PRR FLS2 a leucine receptor kinase flagellin initiates by with leucine receptor kinase BAK1 It how FLS2/BAK1 receptor Here we receptor kinase BIK1 that is upon flagellin on both FLS2 BAK1 BIK1 associates with FLS2 BAK1 vivo BIK1 is by BAK1 BIK1 phosphorylates BAK1 FLS2 flagellin Thr 23 BIK1 is for its on BAK1 FLS2 that BIK1 is upon flagellin subsequently transphosphorylates FLS2/BAK1 to propagate flagellin Importantly bik1 are compromised flagellin to nonpathogenic BIK1 is an MAMP which MAMP receptor to

A renaissance elicitor microbe danger by receptor Microbe MAMP are signa tures typical microbe their a elicitor are similarly recog nized as DAMPs This focuses on MAMP/DAMPs on to iden tify receptor PRR best MAMP/PRR flagellin/FLS2 EF Tu/EFR are detail put into a per spective Both FLS2 EFR are leucine receptor kinase LRR RKs Upon treatment with flagellin FLS2 a heteromeric with BAK1 an LRR RK that as coreceptor for brassinolide receptor BRI1 ofMAMP/PRR for is highlighted by finding that pathogen effector to PRR or that MAMP DAMPs effector are all perceived as danger a stereotypic

A protein Zymoseptoria tritici is for on wheat Stb6 gene Zymoseptoria tritici is Septoria tritici a pathogen wheat globally most damaging pathogen wheat Europe A GFG between tritici wheat Stb6 gene has been postulated for but gene have not been We AvrStb6 by coining trait mapping a between Swiss with a genome a 100 from France We AvrStb6 transformations AvrStb6 a cysteine protein that an on wheat Stb6 gene We 16 nonsynonymous nucleotide that AvrStb6 is very AvrStb6 is a is surrounded by which its to Stb6 AvrStb6 is gene to be tritici contributing to our pathogen GFG between tritici wheat

A for oomycete effector protein into oomycete pathogen by effector protein into where they bacterium is through but analogous for effector are uncharacterized fungus oomycete Here we RXLR EER oomycete effector We Phytophthora infestans RXLR EER protein Avr3a as a reporter for because it RXLR EER that R3a protein We that Avr3a with or RXLR EER is from infestans haustoria that these are not for to haustoria or for replacement Avr3a RXLR EER with alanine singly or coination or with KMIK DDK representing a change that conserves physicochemical protein infestans fails to Avr3a or an Avr3a GUS fusion protein into that these are for We that RXLR EER gene are upregulated during Bioinformatic 425 gene RXLR EER protein infestans genome this protein unparalleled opportunities to how oomycete to

A Xanthomonas uridine monophosphate transferase kinase is on pathogen PAMP at or pathogen effector protein PAMP effector constitute against phytopathogen bacterium a effector protein into to assist or propagation A effector protein have been to but for these effector Here we that Xanthomonas campestris campestris effector AvrAC enhances by Arabidopsis BIK1 RIPK receptor kinase to AvrAC is a uridylyl transferase that adds uridine ′ monophosphate to conceals loop BIK1 RIPK reducing their kinase consequently inhibiting

rust fungus Rust fungus Pucciniales are pathogen most threats to these pathogen their because rust fungus are biotrophs rise technology have presented options for effector gene rust fungus Transcriptome bioinformatics have to to by rusts Thousands gene protein during have been for rust which rust effector effectoromics have pushed ahead further priority effector gene These into rust effector promise to inform spur for managing rust This is protected by copyright All

barley MLA receptor effector powdery mildew pathogen gene nucleotide leucine protein NLR are AVR effector from pathogen NLR gene define fastest gene flowering are arranged gene paralogs contributing to copy NLR a Barley mildew a Mla has been subject to specificities each a but unidentified AVRa gene powdery mildew fungus Blumeria graminis hordei Bgh We applied a transcriptome Bgh AVRa gene AVRa1 AVRa13 effector by Mla1 Mla13 effector gene barley or protoplasts was to Mla1 or Mla13 a hallmark NLR receptor AVRa1 AVRa13 are that certain MLA receptor to effector They are ancient effector because are wheat powdery mildew AVRA1 by barley MLA1 is retained Arabidopsis that AVRA1 MLA1 or that its an evolutionarily AVRA1 transcriptome Bgh for Bgh that is ially to geographic

effector protein is integral to pathogen pathogen effector both into but no method exists to discriminate between these localizations We method for whether an effector or protein to uncovers to both effector protein namely depletion glutamic acid acidic amino acid charged amino acid enrichment amino acid predicts effector with a sensitivity % a false % improving accuracy cysteine classifiers by > % does not on a peptide correctly predicts unconventionally protein secretome saprophytes as as pathogen are for protein Rust pathogen have proportions protein but these are for effector pioneers It will will be for if an effector to or if it enters

Arabidopsis kinase regulates gene through animal microbe through pathogen protein to kinase gene is about how kinase gene We that pathogen Arabidopsis kinase MPK4 exists with WRKY33 This on MPK4 substrate MKS1 with Pseudomonas syrin gae or flagellin to MPK4 phos phorylation MKS1 Subsequently with MKS1 andWRKY33 are fromMPK4 WRKY33 promoter PHYTOALEXIN DEFICIENT3 PAD3 encod an enzyme for camalexin Hence wrky33 are impaired PAD3 mRNA camalexin upon That WRKY33 is an effector MPK4 is further supported by PAD3 mpk4 wrky33 backgrounds Our estab lish between MPK4 an how a kinase can gene by releasing upon

Arabidopsis RIN4 is a effector AvrRpt2 RPS2 pili effector protein from pathogen to express R protein that to a effector by activating We that effector from Pseudomonas syringae modify Arabidopsis RIN4 protein Here we that AvrRpt2 a effector RIN4 its disappearance This is RPS2 Arabidopsis R protein that senses AvrRpt2 RIN4 with AvrRpt2 Conversely RIN4 RPS2 lethality RPS2 RIN4 We that RIN4 is AvrRpt2 that perturbation RIN4 RPS2 RIN4 is a convergence for at syringae effector

secretory Yersinia Salmonella Shigella Bordetella Pseudomonas aeruginosa enteropathogenic Escherichia coli adhering at a to protein to destroy or These consist a apparatus ~25 protein an protein by this apparatus Some these protein are effector which are into are translocators which help effector to Most effector on cytoskeleton or on A protein injected by enteropathogenic coli serves as a receptor for docking bacterium itself at pathogen where they are both eliciting so or They consist Hrp protein building a apparatus groups effector harpins protein Harpins are protein are to be into a coherent picture is clearly basic to be answered is about how apparatus fits to protein animal It is more mysterious for where a thick has to be crossed spite these haunting as a fascinating device

gene from Rice pathogen Magnaporthe oryzae To rice Oryza sativa ascomycete fungus pathogen Magnaporthe oryzae a battery effector some with AVR which are by R protein To gene from oryzae we examined DNA protein gene from genome looked for an with AVR This scale more / than nucleotide 1032 protein gene Nucleotide oryzae 46 a collection was extremely theta = suggestive pathogen dispersal no between DNA AVR was we genome resequencing Ina168 an oryzae that AVR gene a 68 comprising 316 effector gene were Ina168 but absent asseled these 316 gene AVR gene AVR Pia AVR Pii AVR Pik/km/kp to AVR gene whose are rice possessing R gene AVR Pia AVR Pii have by gene / AVR Pik/km/kp has by nucleotide substitutions gene /

AvrPtoB LysM receptor kinase CERK1 to on on a receptor PRR that to ligands as path ogen PAMP [ ] To over come such bacterium effector protein into that [ ] PRR PAMP by effector to these are Here we that Arabidopsis LysM receptor kinase CERK1 [ ] which is for chitin elicitor resis tance to pathogen an restrict on This is supported by fact that CERK1 is a effector protein AvrPtoB which all through this receptor AvrPtoB ubiquitinates CERK1 kinase CERK1 for vivo We that CERK1 is a determinant but its is by bacterium AvrPtoB Our a for immu nity against bacterium a for AvrPtoB E3 ligase

AvrPtoB A effector that both elicits with Pseudomonas syringae tomato DC3000 is a pathogen for studying tomato Arabidopsis DC3000 uses a to effector protein into effector are to by enhancing to trapped AvrPtoB effector elicits when tomato Pto protein Pto AvrPtoB to tomato Here we examining AvrPtoB "trump " is to explain how protein successfully to effector that

bacterium an aqueous for has a influence on above ground phyllosphere fields ecosystems but this is not Previous have emphasized as a Here we that pathogen an aqueous is phyllosphere effector such as Pseudomonas syringae HopM1 aqueous are to transform syringae into pathogen immunodeficient Arabidopsis thaliana under Arabidopsis quadruple simultaneously defective a AtMIN HopM1 could not only be to reconstitute basic but exhibited dyshomeostasis endophytic commensal community phyllosphere These a framework for phyllosphere

effector ner BAK1 to disrupt MAMP receptor impede    pathogen have to with For ubiquitous pathogen Pseudomonas syringae injects effector AvrPto AvrPtoB to intercept convergent stimulated by microbe MAMP precise effector We that AvrPto AvrPtoB Arabidopsis receptor kinase BAK1 a ner both flagellin receptor FLS2 brassinosteroid receptor BRI1 This interferes with ligand FLS2 with BAK1 during It impedes BAKI1 to other MAMP brassinosteroid AvrPto BAK1 to be from AvrPto Pto for effector These uncover a where effector transmission through a MAMP receptor

evasion on to has receptor that pathogen more protein These have shed on our by which bacterium how pathogen have to or these

nanomachines for protein into ofthe most exciting developments ofbacterial is thatmanypathogens nanoma chines to bacterially effector protein into These effector protein a for pathogen ’ these protein machines is T3SS T3SSs are are not only by bacterium to vertebrates or but by bacterium that are syiotic to or insect A T3SSs is needle a supramolecular that passage protein envelope Working conjunction with needle engages sequential moves them envelope ultimately them into T3SSs makes them for

to  effector  to pathogen machines to effector during Such are or prototypical is Agrobacterium tumefaciens DNA which oncogenic nucleoprotein icles to Other pathogen Bordetella pertussis Legionella pneumophila Brucella Helicobacter pylori machines to effector protein to milieu or

BED receptor spectra to yellow rust wheat yields by ~25% globally pose a threat to can can be selected through breaks down pathogen as experienced with wheat yellow stripe rust fungus Puccinia striiformis tritici Pst This highlights need to gene that alone or coination our modes Here we yellow rust gene Yr Yr5 YrSP from hexaploid wheat Triticum aestivum each having a We that Yr5 which to a Pst is from Yr YrSP is a truncated Yr5 with 99 % identity All Yr gene to a gene on chromosome 2B nucleotide leucine protein NLR with a canonical zinc finger BED domain3 that is from those NLR wheat protein We diagnostic to haplotype for marker assisted to expedite stacking Yr gene Our that BED NLR gene can to such as wheat yellow rust

Behind actions effector protein most bacterium on T3S which translocates effector protein into effector to pathogen multiplication effector is which is upon pathogen by receptor protein effector with proteasome protein phytohormone formation cytoskeleton vesicle gene This our on effector protein with for effector protein or effector are

Bidirectional RNAi uptake external RNA pathogen such as Botrytis Verticillium severe We that Botrytis cinerea RNA Bc sRNA into to silence gene Such sRNA effector are by Botrytis cinerea Dicer protein Bc DCL1 Bc DCL2 Here we that sRNA that Bc DCL1 Bc DCL2 Arabidopsis tomato silences Bc DCL gene attenuates exemplifying bidirectional RNAi sRNA trafficking between fungus This can be to simultaneously We that Botrytis can take up external sRNA stranded RNA dsRNAs Applying sRNA or dsRNAs that Botrytis DCL1 DCL2 gene on fruits vegetables flowers grey mould Such pathogen gene RNA a environmentally friendly fungicides

Breaking effector to microorganism to inhabit an enormous niches is a niche offering a constant supply but to this niche ranging from passive to reactions have to be pathogen have to break if not all these For this purpose they effector into to with is organized layers effector follows this same to a hierarchy effector orchestration this we latest regarding at which effector attention is given to those effector whose is Additionally we compare to effector

Catch me if you can effector To favor es tablishment bacterium have gained to effector through effector protein be addressed to where they are to a Here we how by effector as as pathogen capabilities by have contrib uted to further our about intricate mo lecular that are between their bacterium

cDNA AFLP unravels a genome hrpG regulon pathogen Xanthomonas campestris vesicatoria Hrp protein is for pathogen Xanthomonas campestris vesicatoria hrp gene is by HrpG a HrpX an AraC cDNA AFLP technique hrpG hgi hrpG repressed hgr cDNA fragments were defining a hrpG regulon campestris vesicatoria most gene hrpG regulon was on hrpX cDNA fragments to hrp gene flanking All other gene to be scattered over chromosome plasmids gene protease a XopJ XopB Xanthomonas outer protein YopJ from Yersinia protein AvrPphD Pseudomonas syringae XopB is by Hrp deletion hgi gene a This that cDNA AFLP is a to prokaryotic transcriptomes to gene contributing to Xanthomonas effector protein

mediators Caenorhabditis elegans pathogen nematode Caenorhabditis elegans is as a versatile which to pathogen worm has to be both animal gene toxin killing on worm by a pathogen has that a gene worm susceptibil ity these gene PhoP/Q regulators Salmonella LasR Pseudomonas aeruginosa have been that worm will be relevant to other keeping with this aimed at gene have that have been suggested to a animal such as p38 kinase forward this to be done design more sensitive to effector further exact between pathogen elegans’

Cladosporium Avr2 tomato Rcr3 protease for Cf How pathogen is mysterious between  pathogen   Avr protein     protein is exception than rule During Cladosporium fulvum Avr2 protein into tomato leucine receptor Cf protein a that requires tomato cysteine protease Rcr3 We here that Avr2 Rcr3 that Rcr3 Avr2 Cf protein to an

Cladosporium fulvum effector Weapons with Tomato this I recount my personal My drive to pathogen was to alternatives for agrochemicals which was after book "Silent Spring" by Rachel Carson I on my at Laboratory Phytopathology at Wageningen University where I have worked for my entire career on between Cladosporium fulvum tomato I to Avr gene from pathogen R gene from gene classical have been breeders can with R gene durable environmentally safe against pathogen some this require when R gene cannot be introduced by classical

Cleavage Arabidopsis PBS1 by a effector R protein are to as receptor for ligands or by pathogen Avr protein most Avr protein are by which they R protein have not been Arabidopsis to Pseudomonas syringae AvrPphB requires RPS5 a R protein that have a nucleotide leucine PBS1 a protein kinase AvrPphB was to proteolytically cleave PBS1 this cleavage was for RPS5 which that AvrPphB is its enzymatic

themes     animal pathogen animal This presents developments animal with respect to effector protein to both animal are relation to pathogen will greatly add to our defining pathogen

animal bacterium pathogen to invade animal on a protein that effector protein into effector protein a such as rearrangements cytoskeleton effector varies but have parallels between some effector protein from animal bacterium These that pathogen animal

receptor uncovers protein by pathogen receptor to   pathogen     receptor nucleotide leucine NLR protein a nucleotide NB followed by a leucine LRR are initiators     that NLR with canonical an     These composite receptor are to arise from fusions between NLR that serve as "baits" for   pathogen  effector protein enabling  pathogen   names have been to these protein " " " sensor" We adopt argue for " " or NLR IDs which describes fusion assigning a universal We have scanned    genome for NLR IDs to evaluate integrations sensor/ flowering We manually wheat brassicas a subset NLR IDs cultivated wheat We have examined NLR fusions that     that some re occurring fused to NLR overlap with   pathogen   confirming that they as baits for   pathogen While some have been for durable to    pathogen We have built a reproducible pipeline for detecting     receptor We hypothesize that NLR IDs that we to protein by pathogen that this can be deployed to discover sources

Comparing engaged  effector employ modes their to resist pathogen is to as that is by to microbe is by pathogen effector is as effector At some extensively that is to be by an are more prolonged than those has that synergistic are evident which amplify compensatory dominate explaining robustness against seem to a differently

Genome Citrus Huanglongbing Bacterium Candidatus Liberibacter asiaticus Through Metagenomics Citrus huanglongbing is most citrus It is spread by citrus psyllids is with a titer phloem by any uncultured alpha Proteobacteria Candidatus Liberibacter asiaticus americanus africanus A circular asiaticus genome has been by metagenomics DNA extracted from a asiaticus psyllid 23 genome has an average 36 % GC a percentage gene both motility % % which to its asiaticus to have a for aerobic respiration is auxotrophic for at amino acid with its asiaticus as as typical free or  colonizing degradative enzyme asiaticus to have all I gene needed for both multidrug toxin effector  Multiprotein asiaticus as an branching divergent Rhizobiaceae This is genome an uncultured alpha proteobacteria that is both an     pathogen   insect symbiont

between gene their gene consequences for During pathogen an effector which Some these effector can be or by R protein from are then AVR protein This R AVR gene are frequently to genome has accelerated AVR gene fungus fungus agronomically While AVR gene by their R gene were more more between AVR R gene are AVR gene by R gene R gene AVR gene AVR gene AVR gene by its R gene cooperating R gene both to an AVR gene These were showing a with their but could from agronomic were through interspecific hybridization or introgression gene from into cultivated this we some R AVR between fungus that were their implications for AVR gene R gene

for by Phytophthora sojae effector Avr1b genome oomycete pathogen superfamilies effector protein protein RXLR EER these effector to Here we that Phytophthora sojae effector protein Avr1b can positively to can by mouse BAX protein yeast soybean Glycine max Nicotiana benthamiana We K Avr1b protein that or Avr1b to Avr1b with Rps1b gene soybean are at oomycete RXLR EER effector we that these soybean these that are for Avr1b with protein that these are for very oomycete effector that them

LysM effector Ecp6 Prevents Chitin Multicellular upon pathogen PAMP Chitin is walls chitin oligosaccharide as PAMP pathogen effector protein to PAMP to Here we that LysM effector protein Ecp6 pathogen Cladosporium fulvum through perturbation chitin During Ecp6 sequesters chitin oligosaccharide that are from walls hyphae to This a by pathogen because LysM effector are

Convergent a protein by pathogen effector from kingdoms life While principles governing are becoming clear its organization pathogen are We generated a protein protein effector from ascomycete pathogen Golovinomyces orontii Arabidopsis thaliana protein We this with for eubacterial pathogen Pseudomonas syringae oomycete pathogen Hyaloperonospora arabidopsidis protein onto which intraspecies interspecies pathogen effector converge Phenotyping 124 Arabidopsis effector interactor a correlation between intraspecies interspecies convergence altered effector most heavily protein colocalized subnuclear foci adaptively selected Arabidopsis gene are for with effector Our existence a pathogen that is Arabidopsis ions while neighborhood effector

Cultivar assigned to avrPphF Pseudomonas syringae phaseolicola bean avrPphF gene was from Pseudomonas syringae phaseolicola Pph on its to bean R1 gene for such as Red Mexican avrPphF d open frames which were both for was on a 154 kb plasmid pAV511 Pph RW60 Pph pAV511 displayed a to a such as Tendergreen Canadian Wonder Tendergreen was restored to RW60 by avrPphF alone subcloned avrPphF pAV511 greatly accelerated reaction by RW60 Canadian Wonder A gene from pAV511 avrPphC which controls to soybean was to avrPphF Canadian Wonder but not Red Mexican avrPphF conferred soybean avrPphF how effector protein from pathogen have to be by R gene be classified as by gene

Deacetylation chitin oligomers pathogen pathogen that are threats to Here we a secretory polysaccharide deacetylase A1 from fungus Verticillium dahliae most notorious pathogen Verticillium that facilitates through deacetylation chitin oligomers whose acetyl to lysine LysM receptor for ligand Polysaccharide deacetylases are fungus bacterium insect marine invertebrates have been to developmental than A phylogenetics more than protein with polysaccharide deacetylase that V dahliae A1 subtree a protein from Verticillium as as Fusarium pathogen that pathogen have adopted chitin deacetylation as a We that a Fusarium A1 is for cotton This a substantial polysaccharide deacetylases fungus a subtle whereby deacetylation chitin oligomers converts them to ligand chitosan representing a preventing chitin by pathogen

Defended to nines 25 gene for R protein have R gene which to a pathogen R gene to be maize Zea mays Hm1 was over 25 ago then R gene have been isolated protein have to Here we a meta 314 R gene R gene or receptor we distinguish by which R protein can elevate or or pathogen on by receptor protein receptor kinase or pathogen by nucleotide leucine receptor or through effector through executor gene passive or reprogramming R gene are only for a proportion R gene a clearer is will be for rational R gene

Gaining a Rice Xanthomonasoryzaepv oryzaeInteractions for Durable Rice Rice is most rice is for ensuring limit rice them BB byXanthomonas oryzaepv oryzae Xoo is very BB reduces rice yields productive irrigated rainfed lowland ecosystems is spreading to other rice ecosystems as Being a pathogen Xoointerferes with a exchange rice rice toXooinvolves between R gene rice Avr gene ofXoo covering most gene except recessive ones to BB rice has been intensively at 44 gene conferring to BB have been rice hybrids have been existence ofXooin realm a climate necessitates gene intensification gene This discusses about occurrence BB rice betweenXooand rice gene rice gene BB gene from ofOryzaand presents a on to achieve goal

protein underlies flax gene flax rust gene protein R protein pathogen Avr protein through their protein or through R Avr protein imposes against Avr effector pathogen effector through through than Here we that flax rust fungus AvrLS6 gene whose are by L5 L6 L R protein flax are with from rust AvrL56 from Avr necrotic when flax gene R gene from avr do not between AvA56 for diversifying acting on these gene they have been a gene with flax R gene Yeast hybrid that is on R Avr protein recapitulate Escherichia coli AvrL56 protein that that escape nevertheless maintain a stability that amino acid R Avr protein We that with at R Avr gene an alternative outcome pathogen to with simple balanced for nonfunctional R Avr gene

by Yersinia effector YopJ a ubiquitin protein protease Yersinia effector YopJ are both animal pathogen as as symbiont These YopJ were to as cysteine protease catalytic triad protease was for mitogen protein kinase MAPK κB NF κB animal for for YopJ were to be ubiquitin which are covalently added to protein YopJ exert their on by disrupting this posttranslational

by Pseudomonas syringae pathogen differ dramatically but for these unclear bacterium phytopathogen Pseudomonas syringae effector TTEs are for instrumental structuring exhibit between To capture gene syringae we screened 11 for TTE coupled this nearly saturating with from a collection diseased TTE vary dramatically all syringae clades surprisingly few TTEs are all Those that are requirements for We that hopM1 to dramatic we that phylogenetics informed can be to TTEs dynamism TTE is mirrored by phytotoxins highlighting both determination We these draft genome plus genome to genome for syringae we this to that pseudomonad These acquisition a megaplasmid by a clade cucuer pathogen This megaplasmid a a protein which dramatically both these pan genome

PAMP     line to on   pathogen PAMP by receptor PRR This to PAMP PAMP recognised by only a handful PRR are characterised For most they correspond to protein with a ligand ectodomain PRR with protein that as adapters or amplifiers to achieve functionality PRR is by PRR their protein by effector

effector Leptosphaeria maculans genome affected by fungus are biotechnological that are by animal fungus are fungus to their tractability fungus are pathogen or mutualists are to analyse effector gene their this we genome ascomycete Leptosphaeria maculans its protein effector maculans genome has an unusual biite with alternating guanine cytosine equilibrated adenine thymine AT homogenous nucleotide composition AT genome effector gene both which are affected by a genome This for effector underpins fungus to to constraints

effector accelerates potato phytophthora infestans gene Potato is ’ fourth it continues to endure a by pathogen Phytophthora infestans R gene into potato Solanum tuberosum is best for managing but are slow inefficient We a effector gene computationally from infestans genome to R gene An initial 54 effector a peptide a RXLR was profiled for or Avr on Solanum tentative Avr were RXLR effector IpiO stoloniferum papita more distantly bulbocastanum source R gene Rpi with stoloniferum cosegregation to infestans to IpiO IpiO with Rpi a heterologous Nicotiana benthamiana IpiO as Avr A gene to stoloniferum Rpi sto1 papita Rpi pta1 which are equivalent to Rpi Our that effector R gene Avr gene at an unprecedented promises to potato

effector MiSSP mutualistic fungus Laccaria bicolor stabilizes Populus JAZ6 protein represses jasmonic acid JA responsive gene Ectomycorrhizal fungus such as Laccaria bicolor forest sustainability by limiting to their    through a mutualistic syiotic with roots We have that  effector protein MiSSP Mycorrhiza Protein by bicolor is for syiosis with trees reasoning behind this was We here that MiSSP with protein PtJAZ6 a jasmonic acid JA gene Populus As with other JASMONATE ZIM JAZ protein PtJAZ6 with PtCOI1 JA coronatine PtJAZ6 is degraded     after JA treatment between MiSSP PtJAZ6 is to protect PtJAZ6 from this JA MiSSP is to or mitigate JA on bicolor roots We that MiSSP by bicolor can be complemented by transgenically varying PtJAZ6 or through JA gene We that bicolor to arbuscular fungus pathogen mutualism by blocking JA through MiSSP with PtJAZ6

effector Chitin by Magnaporthe oryzae Is for Rice receptor to defend themselves from pathogen These receptor pathogen PAMP that to rice Oryza sativa chitin elicitor protein CEBiP chitin oligosaccharide from walls pathogen Here we that rice fungus Magnaporthe oryzae overcomes this line by secreting an effector protein LysM Protein1 Slp1 during rice We that Slp1 accumulates at between rice can to chitin is to chitin oxygen gene we that Slp1 competes with CEBiP for chitin oligosaccharide Slp1 is by oryzae for exerts a on lesion By gene CEBiP rice oryzae to rice Slp1 We that Slp1 sequesters chitin oligosaccharide to PAMP rice spread fungus

effector against pathogen R gene against pathogen is not adequately explained by terms pathogen PAMP or effector it is that this is effector ETD Unlike ETD is by R gene localised receptor protein RLPs that engage receptor kinase SOBIR1 to this is initiated by pathogen effector is with ETD only after an elapsed period endophytic pathogen this opinion we on ETD against foliar pathogen

effector From pathogen to have to sense to pathogen have to intercept by pathogen effector convert it to to is a nucleotide /leucine NLR receptor that effector effector NLR connect ways to a to boost programs effector displays robustness against pathogen disturbance by employing compensatory mobility some NLR coordination flexibility to fine tune outputs a NLR to chromatin by balancing actions repressing activating to program dynamically for

effector protein from secretome pathogen effector protein that to effector into pathogen effector fungus has been challenging to a unifying such as effector are commonly from secretome on such as cysteine which suffers from poor accuracy We which pioneers application to effector improves effector from secretome on a achieving sensitivity over 80% that discriminate effector from noneffectors are predominantly weight protein net charge as as cysteine serine tryptophan We that is when with for priority effector is program for effector on Our will effector improve our effector pathogen is at http // csiro au

effector pathogen Commonalities amid fungus oomycete are microorganism that a pathogen These are modulators that an effector protein to Genome catalogues effector pathogen with some harboring effector gene a fraction these effector gene protein with weak or no to protein have unexpected amid This reviews our effector these We that there is for effector pathogen but that some have expanded from a ancestor by folds such as oomycete WY MAX are not predictive precise effector but serve as a chassis to protein integrity while enough for effector to protein Further effector arise short linear duplications oligomerization

replacement an effector gene oomycete Phytophthora sojae CRISPR/Cas9 Phytophthora sojae is an oomycete pathogen soybean As a its sojae has a for oomycete pathology techniques for gene replacement have hampered Phytophthora Here we a CRISPR/Cas9 enabling genome editing sojae RXLR effector gene Avr4/ as a we that a template repair Cas9 DNA strand breaks DSBs sojae was by end joining NHEJ primarily short indels Most were homozygous as a gene con by Cas9 cleavage When donor DNA was directed repair HDR was which replacement Avr4/ with NPT II gene By testing NHEJ HDR gene replacements soybean we have Avr4/ to by soybean R gene Rps4 Rps6 but to by these Our a for Phytophthora which avenues for this pathogen

elicitor effector R gene paradigm a lifetime supply can broadly under bar icrobe a under bar ssociated under bar olecular under bar atterns MAMP PAMP but microbe express a suite effector protein that to these have other receptor R protein that these pathogen effector pathogen can subsequently or delete their effector to avoid at risk a cost with those effector is other things MAMP protein that pathogen effector R protein ways which pathogen effector suites R gene These practical ramifications for durability for uses to help clarify what we know to areas that are ripe for further investigation

effector challenges fungus have that on for all upon to modify to sequester for to as "effector" is assumed to be governing that determines effector protein are capable to accommodate invaders This focuses on effector fungus for uptake effector by fungus We place emphasis on effector difficulties with effector that have helped effector to We effector fungus examine how CRISPR/Cas9 technology a avenue for accelerating our effector

 effector    protein other to circuitry these as effector became accepted as for a This effector highlights that have from pathogen effector

effector Gene Fungus Leptosphaeria maculans pathogen an protein SSPs acting as effector that to SSP gene are waves concerted at stages To date is about their genome Ascomycete Leptosphaeria maculans alternating gene GC gene poor AT AT mosaics encompassing genome are effector gene that namely no or during axenic cultures to during oilseed rape Brassica napus Here we investigated histone histone H3 lysine methylation H3K9me3 concerted effector gene maculans For this purpose we silenced players heterochromatin maintenance HP1 DIM by RNAi By HP1 GFP as a heterochromatin marker we that almost no chromatin condensation is visible which LmDIM5 was silenced by RNAi By genome oligoarrays we 369 or 390 gene silenced LmHP1 LmDIM5 transformants during axenic culture clearly favouring SSP gene AT effector gene GC to their during axenic culture These that by HP1 DIM represses at effector gene AT during axenic culture Our is that a lift chromatin allowing a to

fatty acylation drives enhances effector protein from Pseudomonas syringae pathogen animal to traffic effector protein into R gene to some effector Avr protein On r Avr protein can to pathogen We that effector from Pseudomonas syringae are to that enhances Avr protein requires consensus myristoylation Avr protein can be myristoylated These prokaryotic effector a eukaryote posttranslational to where they

External lipid PI3P pathogen effector into animal pathogen animal effector protein that are transferred into to pathogen oomycete effector protein with RXLR EER that into We here that effector pathogen fungus RXLR that oomycete RXLR to phospholipid phosphatidylinositol phosphate PI3P We that PI3P is abundant on outer on some animal All effector could human that PI3P effector be very animal human into both animal lipid raft endocytosis Blocking PI3P inhibited effector therapeutic avenues

tomato protease Rcr animal but these have not been Here we that protease Rcr3 tomato is by P69B other subtilases SBTs a solanaceous Rcr3 is a papain Cys protease PLCP tomato that both against Phytophthora infestans against pathogen Cladosporium fulvum syn Passalora fulva prevalent that Rcr3 protease can themselves at pH we that catalytically proRcr3 precursors are processed into mature mRcr3 isoforms ProRcr3 is processed by P69B other Asp SBTs solanaceous through SBT redundancy effector EPI1 infestans can Rcr3 by inhibiting SBTs that this effector by preventing Rcr3 protease Rcr3 Nicotiana benthamiana requires a SBT from a subfamily that have convergently solanaceous or are very ancient frequent incidence Asp cleavage Rcr3 protease solanaceous that protease by SBTs is a illuminating a that

    pathogen  effector Live imaging assisted by fluorescent has been to focused secretory warfare that between pathogen that feed on     pathogen succeed through spatiotemporal a  effector protein to     Some effector can be by appressoria before     where they move into neighbouring to prepare them before This latest our between their pathogen on effector

Flagellin that is by Pseudomonas syringae effector Arabidopsis NONHOST1 NHO1 is for limiting Pseudomonas bacterium but completely ineffective against bacterium Pseudomonas syringae tomato DC3000 this observation Here we that NHO1 is by flagellin bacterium syringae tabaci flagellin is unable to NHO1 multiplies than does bacterium symptoms on Arabidopsis DC3000 possesses flagellin that is potent NHO1 but this is by DC3000 a DC3000 effector protoplasts indicated that at effector HopS1 HopAll HopAF1 HopT1 HopT1 HopAA1 HopF2 HopC1 AvrPto are capable flagellin NHO1 effector HopAll is both animal    bacterium When Arabidopsis HopAll nonpathogenic hrpL bacterium purified phytotoxin coronatine a    syringae flagellin NHO1 These that flagellin incluced an A DC3000       by that to specialization DC3000 on Arabidopsis

Flax rust gene is on protein flax flax rust to formulation gene R gene Avr inhibitor gene I rust pathogen R gene have been from flax all protein Toll Interleukin receptor R gene nucleotide leucine TIR NBS LRR Avr gene have been from flax rust protein with no between no bases It is postulated that Avr protein have effector genotypes are by with R protein to rust between R Avr protein is flax flax rust both R Avr gene have signatures diversifying existence a coevolutionary between its rust pathogen

From to A for     pathogen  effector for postulates that    protein by monitoring guarding their   pathogen  effector We posit that guarded effector  are evolutionarily unstable     for R gene on or R gene guarded effector  are subject to opposing forces to manipulation by effector weaker to improve effector stronger  effector  gene or a could relax constraints a that would be solely  effector  There is for this from  effector  Pto Bs3 RCR3 RIN4 We between their hypothesize how have ways to

Fugal effector protein It is accepted that most gene that are effector Most effector are cysteine protein a has been for a few them Avr2 Avr4 Cladosporium fulvum which    cysteine protease protect chitin walls against   chitinases effector are or by protein that reside     or on effector but uptake is not effector that to relate to whether they or with their protein seem to favor  effector gene to amino acid substitutions seem to favor jettison  effector gene

Yersinia effector YopJ Yersinia YopJ apoptosis by blocking MAPK NFkappaB YopJ is a cysteine protease that cleaves a reversible translational ubiquitin or a ubiquitin protein YopJ are animal     pathogen as as a   symbiont a universal or modulating a YopJ to its as a ubiquitin protein protease its with respect to are this

effector protein past pioneering Harold Flor on flax flax rust fungus culminated his It took nearly 50 before Avr gene his was Initially Avr gene were by reverse from but genome their from fungus by a coination It is believed that most Avr gene effector that by pathogen effector protein effector are or by protein that reside on or an effector as implies that an effector guardee is guarded by protein that senses manipulation guardee to effector this we on effector some pathogen those some fungus for which no has been

effector can be colonized by fungus that have adopted ranging from syiotic to is governed all by effector These effector to accom modate invaders them with effector zone between hyphae or are transferred to This describes effector reper toires 84 colonizing fungus We on that these effector to or compatibility com mon nodes that are by effector into effector we address issue effector uptake open challenges

RNA by Hijacking RNA Botrytis cinerea causative gray mold is an pathogen that infects more than 200 Here we that some cinerea RNA Bc sRNA can silence Arabidopsis tomato gene These Bc sRNA hijack RNA RNAi by to Arabidopsis Argonaute AGO1 selectively gene Arabidopsis ago1 exhibits to cinerea cinerea dcl1 dcl2 that can no longer these Bc sRNA displays on Arabidopsis tomato this pathogen transfers "" sRNA effector into to achieve which a naturally occurring RNAi as an advanced

Genome an Potato   pathogen Pest pathogen jeopardise ever 19th potato has exemplified this threat oomycete pathogen Phytophthora infestans undergoes shifts successive migration asexual phenotypic genotypic bases these sweeps are but need to to pathogen Here we to document a 13_A2 infestans its displacement other to exceed % pathogen Britain less than We that 13_A2 are most on cultivated potatoes outcompete other Genome a 13_A2 effector gene Copy variations gene gains amino acid replacements effector gene 13_A2 to aggressiveness to drive this displacement Importantly 13_A2 intact Avrblb1 Avrblb2 Avrvnt1 effector gene that potato R receptor gene Rpi Rpi blb2 Rpi vnt1 These a for deploying to mitigate 13_A2 how pathogen monitoring with genome informs epidemics

Genome     pathogen why bigger can be fungus oomycete are pathogen Over past genome more than these pathogen have been genome genome parasites symbiont have been over time genome pathogen have been shaped by expansions these gene protein are frequently reside genome Here we these adaptable genome their we which genome has contributed to We how genome expansions have had an on conflict between these pathogen their

Genome Gene Powdery Mildew fungus Tradeoffs Extreme Parasitism Powdery mildew are phytopathogen whose are entirely on this life style We genome barley powdery mildew Blumeria graminis hordei Blumeria as as a with powdery mildew on dicotyledonous These genome massive retrotransposon genome gene missing gene enzyme carbohydrate enzyme transporter reflecting their redundancy an exclusively life style 248 effector Blumeria genome very few less than define a all mildew that most effector adaptations

Genome potato   pathogen  Phytophthora infestans Phytophthora infestans is most   pathogen   potato a for oomycete a fungus eukaryotes that are to such as brown algae diatoms As potato mid nineteenth infestans has had a tremendous on human displacement To this day it affects by most potato fourth a alternative to cereal for feeding annual potato to are conservatively estimated at $ billion this   pathogen  is challenged by its speed to such as Here we infestans genome which at to 240 megabases is by most genome so chromalveolates Its from a repetitive DNA accounting for to 4% genome with other Phytophthora genome turnover  effector protein gene that are during or are to have that These  effector gene are to expanded infestans genome This a adaptability   pathogen  to underpins its

Genome pathogen Ralstonia solanacearum Ralstonia solanacearum is a pathogen with a an unusually It is a for dissection governing We here genome its GMI1000 megabase genome is organized into replicons a chromosome a megaplasmid Both replicons have a mosaic for acquisition gene through gene mobile with percentage G+ bias have an genome genome protein with a attachment were effector protein can be Over 40 were with other genome that pathogen animal pathogen harbour arrays effector

to intimately with bacterium bacterium have ostensibly gene that them to to identities such gene are their are We 484 genome from roots Brassicaceae poplar maize We then 83 genome to thousands gene genome bacterium more carbohydrate fewer mobile than genome do We from gene other serving microbe microbe competition between bacterium We 64 protein that some are with fungus oomycete This expands genome microbe for through microbiome

mining effector Pseudomonas syringae yields picks for all TTSS prospectors pathogen animal a TTSS to effector protein into Because effector are heterogeneous there has not been a to gene them pathogen genome our inventories are incomplete A pre closure draft Pseudomonas syringae tomato DC3000 a pathogen tomato Arabidopsis has supported complementary which 36 TTSS protein more effector this These advantages coining they yield into TTSS effector syringae effector all TTSS pathogen for finding TTSS effector other bacterium that have genome

Getting  effector protein on their to     most pathogen on hrp gene which to to hrp gene a TTS that vectorial effector protein both as as into effector protein is AvrBs3 from Xanthomonas campestris vesicatoria pepper tomato AvrBs3 hypertrophy symptoms a gene reaction Intriguingly AvrBs3 has characteristic that it transcriptome Here we TTS campestris vesicatoria on protein bacterium

Go for kill how effector to coat pathogen R protein perceive pathogen effector from pathogen to effector R protein are nucleotide leucine NB LRR protein which pathogen effector or through Upon by effector protein R protein a oxygen ROS salicylic acid SA a at PR gene Initiation is correlated with a defensive scale reprogramming this we on effector R protein trafficking R protein reprogramming with gaps directions are this

Groovy times pathogen effector microorganism such as fungus oomycete an effector protein that Deciphering effector to how pathogen successfully reproduce on their became a driving paradigm oomycete pathology a effector as as validate that effector gene are forces that drive antagonistic between pathogen

Help wanted helper NLR     nucleotide leucine NLR protein as receptor to pathogen effector some sensor NLR sNLR by their pathogen effector is by which helper NLR hNLR to transduce sNLR into We briefly examples sNLR we then on hNLR requirements sNLR initiated We further how with incompatibility protein mixed kinase pseudokinase MLKL protein informs a plausible for an ancient clade hNLRs RNLs

HIGS Gene pathogen Blumeria graminis Powdery mildew fungus are pathogen that only grow on thousands their agronomical for gene is because transformation protocols are Here we that barley Hordeum vulgare wheat Triticum aestivum stranded or antisense RNA transcripts affects powdery mildew fungus Blumeria graminis Proof for gene was by effector gene Avra10 which but not gene Mla10 fungus could be rescued from Avra10 by a synthetic gene that was to RNA RNAi to silent traffic RNA from into graminis to an RNAi against pathogen

a gene potato to pathogen a virus a nematode nematode gene Gpa2 potato is described it is that gene a gene can to pathogen Gpa2 an R gene gene a approximately 115kb At these gene are corresponds to isolated Rx1 gene that to potato virus while other corresponds to Gpa2 gene that to potato cyst nematode Globodera pallida protein by Gpa2 Rx1 gene an over 88% amino acid identity to leucine zipper nucleotide leucine LZ NBS LRR gene From conservation between Gpa2 Rx1 it is clear that there is a between protein is concentrated LRR effector are more that at this case nematode virus could These underline protein for specificities against pathogen

microbe Shaping         has culminated a that is to resist by pathogen is to as PAMP has to pathogen microbe pathogen acquired to  effector protein to     to allowing  pathogen   to   pathogen  effector protein acquired protein R protein to or monitor   pathogen  effector protein this taking an we discoveries over last decade about

parasite coevolutionary conflict between Arabidopsis downy mildew are constantly exposed to by an pathogen but a somatically adaptive spite this     do not suffer epidemics how    gene that to pathogen gene counteracts   pathogen  gene for effector is to our     RPP13 gene is most gene analyzed to date    Arabidopsis thaliana Here we gene ATR13 that RPP13 we that it too exhibits extreme amino acid diversifying visible both that   pathogen   be locked a coevolutionary conflict at these where attempts to by   pathogen  are matched by capabilities by

pathogen warfare at have sensory to pathogen that These not only pathogen elicitor pathogen PAMP or effector but pathogen on to pathogen ingress are cuticle For those pathogen that penetrate to to water protoplast wounds at are created by enzymatic or integrity is by which pathogen Some fragments oligogalacturonic acid as PAMP elicitor oxygen elevated gene that PAMP a good for studying integrity to be about this

toxin by fungus Alternaria alternata toxin HST by pathogen are weight metabolite with a that as effector or certain pathogen There are by Alternaria alternata which HST are for pathogen have been as pathotypes A alternata because morphological but pathological Chemical HST from pathotypes have been A alternata HST has been extensively HST from germinating conidia prior to aids icipation HST to by their reactions attempts have been to A alternata HST mitochondrion chloroplast a metabolically enzyme have been as each HST to elucidation HST sensitivity HST have supernumerary chromosome HST gene have into A alternata pathotypes We biosynthetic cyanobacterial toxin scenarios that have to such gene

HrpZ Psph from pathogen Pseudomonas syringae phaseolicola to lipid bilayers an ion conducting pore hrp gene bacterium on their to reaction Some hrp gene constitute by which effector protein are ed into Here we that hrpZ gene from bean pathogen Pseudomonas syringae phaseolicola HrpZ Psph is an hrp syringae phaseolicola ed by pathogen Yersinia enterocolitica HrpZ Psph was to stably with liposomes synthetic bilayer Under symmetric ionic nM purified recoinant HrpZ Psph to cis planar lipid bilayers provoked an ion with a unitary conductivity 20 pS HrpZ Psph protein from syringae tomato or syringae ion currents to those stimulated by HrpZ Psph HrpZ Psph ion conducting pore was permeable for cations but did not fluxes Cl Such pore forming during

Pseudomonas syringae effector that can yeast Pseudomonas syringae tomato DC3000 TTSS is for on a that on Cosmid pHIR11 pathogen to an upon TTSS  effector HopPsyA We pHIR11 to that effector HopPtoE AvrPphE Pto AvrPpiB1 Pto AvrPtoB HopPtoF could a HopPsyA on tobacco Arabidopsis Mixed inoculum Agrobacterium that occurred     These with exception AvrPpiB1 Pto inhibited tobacco PR gene PR1a DC3000 elicited an with these an Additionally HopPtoG was as a on an by a hopPtoG these protein functioned to pro apoptotic protein Bax to yeast that these effector as protein a proportion effector that that     is for DC3000 effector a requirement for syringae

effector     pathogen analyzing their with    pathogen by injecting protein effector into exceptional effector was phytopathogen Pseudomonas syringae a bioinformatic These along with that most syringae effector by chloroplasts or mitochondria secretome syringae is a lesson gleaned from a  effector  is to syringae to

receptor receptor to pathogen an that is broadly divided into pathogen or PAMP effector is upon PAMP from pathogen by anchored receptor PRR To this line pathogen into effector that effector ETS Counteracting this synthesize R protein which pathogen effector or Avr These coevolving pathogen between pathogen which is into zigzag have been initially excluded from zigzag have substantiating notion that to fight virus a to that for pathogen most R protein against virus so with antibacterial antifungal R gene typical virus PAMP through receptor Finally more compelling a Avr that genotypes has been to as a integrating virus into pathogen zigzag this we these progresses on significance receptor receptor we a newly that is to DNA virus on receptor translational for

effector protein from secretome with fungus effector protein to We improvements to classifier for effector is trained on a larger effector utilizes a on an ensele classifiers trained on subsets offering views on classification achieves an accuracy 89% with 82% for 59 % for a classifier for effector to be protein protein net charge as as amino acid serine cysteine decreases effector secretome symbiont saprophytes by 40% when with retains value coining a stringent classifier with a false % predicts enrichments effector protein from pathogen a cysteine classifier detects enrichment only will track prioritization confidence effector for validation aid improving our effector is at http // csiro au

effector Converge onto Hubs a generate to by both pathogen pathogen effector protein into where they with protein to We generated an pathogen effector from pathogen spanning eukaryote eubacteria Arabidopsis protein to 8000 other Arabidopsis protein We noted convergence effector onto interconnected protein than connections between effector receptor We for protein that with effector from both pathogen pathogen from kingdoms protein that with a connected hubs to their life

Initiation RPS2 specified Arabidopsis is coupled to AvrRpt2 directed elimination RIN4 have a to pathogen to a pathogen is by R protein that to or a few pathogen This has to suggestions a receptor ligand R protein effector protein AvrRpt2 by Pseudomonas syringae Arabidopsis RPS2 R protein We that RPS2 with Arabidopsis RIN4 that AvrRpt2 elimination RIN4 during RPS2 AvrRpt2 RIN4 elimination rps2 ndr1 Atrar1 backgrounds that this can be achieved an RPS2 we that RPS2 initiates upon RIN4 disappearance than AvrRpt2

into genome     bacterium Xanthomonas campestris vesicatoria by genome   bacterium Xanthomonas campestris vesicatoria is causative pepper tomato which to yield This pathosystem has a for studying Here we genome pepper Xanthomonas campestris vesicatoria 85 which a circular chromosome plasmids genome has a G+ 64 % signatures genome genome comparisons a gene to both Xanthomonas axonopodis citri Xanthomonas campestris campestris a completely from Xanthomonas oiyzae oryzae A 548 coding % are to campestris vesicatoria to a which is for genome 85 all other protein described so bacterium on plasmid is to Icm/Dot human pathogen Legionella pneumophild Coxiella burnetti Comparisons with other completely     pathogen  effector protein other adhesins degrading enzyme polysaccharides

from protein pathogen effector into receptor is receptor nucleotide leucine NLR protein can canonical NB ARC nucleotide adaptor by APAF R protein CED NB ARC leucine LRR that these as effector from pathogen protein to are suspected to be effector or Here we scrutinized 31 entire genome to NLR protein Interpro search Zinc Finger BED ZBED protein a BED rice Oryza sativa was investigated by evaluating to fungus Magnaporthe oryzae rice over knock out This that all had atypical protein into their NLR protein on average % all NLR protein We that modifying ZBED gene This that NLR receptor is frequent is a to protein Further depth examination NLR protein promises to unravel protein

between microbiota pathogen terminates their that at this is engaged cooperative microbe influences by communities This microbe MAMP by receptor PRR that to load MAMP PRR emerges as a that locally sculpts asselages This a more for accommodation microbe pathogen elimination finding that are deployed by symbiont pathogen to dampen is with this but implies pressures on to outcomes on reciprocal between microbiota a shaping microbiota coinations maintaining

    are under constant threat by pathogen armed with a effector to their have that decipher pathogen appropriate impaired mounting a to pathogen has a but interconnecting that are under both These operate at through such as salicylate jasmonate ethylene to fine tune both systemic

Intervention phytohormone by pathogen effector constant struggle between microbe has as as offense pathogen To defend themselves from pathogen rely on elaborate by phytohormone pathogen have adopted innovative to phytohormone Tactics frequently employed by pathogen involve hijacking evading or disrupting hormone crosstalk As reviewed here this is achieved ally pathogen as effector which phytohormone receptor activators repressors other phytohormone Herbivores sap sucking insect employ pathogen such as virus phytoplasma or syiotic bacterium to intervene with phytohormone an phytohormone intervention employed by pests pathogen during their with will ultimately to

devices animal Multicellular eukaryotes coevolve with pathogen which exert pressure on their animal protein nucleotide leucine NLR to pathogen NLR convergently each pathogen by animal NLR have been to be overlap it is to discern principles NLR Here we attempt to articulate these principles We that NLR has for pathogen because its utility as a tightly folded "hair " device into which a virtually limitless platforms can be means to rationally design capabilities to counter

Knowing dancer from dance R gene their with other protein from   pathogen     gene is commonplace attention has turned to how protein that they biochemically to their protein to attention has turned to how protein pathogen for   pathogen what   protein be for that

Lessons effector NLR microbe A effector protein metabolite that to favor effector can receptor nucleotide leucine NLR protein enabling to fight off This between effector their receptor is shaped by intricate exceptionally this we on effector AVR Pik AVR Pia AVR Pii from rice fungus Magnaporthe oryzae syn Pyricularia oryzae their rice NLR receptor Pik Pia Pii to microbe We draw lessons effector NLR that have from studying these effector are broadly applicable to other microbe

Lessons learned from genome Ralstonia solanacearum Ralstonia solanacearum is a pathogen with a an unusually This bacterium can be free as a saprophyte water or soil genome from GMI1000 for an integrative determining bacterium to niches This speculates on some metabolic versatility to metals for motility attachment to external surfaces protein Genome about gene such as those effector It into contributing to bacterium to its to its with

transitions     Colletotrichum fungus deciphered by genome transcriptome Colletotrichum are pathogen that devastate differentiation that are with destruction necrotrophy We here genome transcriptome Colletotrichum higginsianum Arabidopsis thaliana Colletotrichum graminicola maize that both fungus have gene but gene effector pectin degrading enzyme enzyme transporter peptidases are expanded higginsianum Genome that these gene are transcribed successive waves that are to transitions effector enzyme are before during most hydrolases transporter are upregulated later at to necrotrophy Our that preinvasion substantially reprograms gene for

Ligand allosteric ADP primes a NLR pathogen by nucleotide NB leucine LRR receptor NLR Xanthomonas campestris campestris effector AvrAC uridylylates Arabidopsis PBL2 kinase latter PBL2UMP as a ligand to NLR ZAR1 precomplexed with RKS1 pseudokinase Here we cryo electron microscopy ZAR1 RKS1 ZAR1 RKS1 PBL2UMP an intermediate ZAR1LRR with animal NLRLRR is differently positioned to sequester ZAR1 an PBL2UMP is exclusively through RKS1 which with ZAR1LRR PBL2UMP stabilizes RKS1 segment which sterically ZAR1 adenosine diphosphate ADP This engenders a more flexible NB conformational other ZAR1 Our a template for NLR

both effector protein pathogen effector protein operate to Some effector have been to by mimicking exist to predict protein they perform for effector We introduce for effector protein to chloroplasts mitochondria nuclei greater accuracy for chloroplast mitochondrial to other for 652 protein For 10 effector outperforms other predicts a unrecognized chloroplast transit peptide for ToxA effector which we translocates into tobacco chloroplasts Secretome predictions confocal microscopy that rust fungus have effector that chloroplasts or nuclei is method for effector localisation is a for prioritizing effector for investigations

Manipulation phytohormone by effector pathogen phytohormone a salicylic acid SA jasmonic acid JA ethylene ET are for against a while regulators such as auxins brassinosteroids BRs cytokinins CKs abscisic acid ABA gibberellins GAs to To successfully pathogen fungus oomycete have to with phytohormone with help effector These protein toxin polysaccharides as as phytohormone or phytohormone mimics Such pathogen effector phytohormone by altering hormone by interfering with phytohormone or by altering or blocking phytohormone this we outline by phytopathogen to phytohormone to

MAPK Mitogen protein kinase MAPK are modules receptor/sensor that transduce stimuli into eukaryotes MAPK pivotal against pathogen this we upstream receptor/sensor MAPK These MAPK MAPK have as battlegrounds pathogen MAPK is earliest after pathogen /microbe PAMP/MAMP pathogen effector MAPK are / / hormone oxygen ROS stomatal closure gene phytoalexin strengthening pathogen employ effector to MAPK to

into epidemiology phytopathogen Xanthomonas Xanthomonas is a pathogen whose a these phytopathogen have ecology epidemiology Xanthomonas are as producers polysaccharide xanthan pharmaceutical industries This polymer has been phases this we summarise on from Xanthomonas opportunities that this body has for health

Mitotic Recoination Genome Forest pathogen Phytophthora ramorum alien have must to Given invasions this is sometimes paradox Phytophthora ramorum is to asexual presumed clonal It is for sudden oak United States sudden larch Europe ramorum North America Europe We genome 10 to how this pathogen can paradox Mitotic recoination MR with transposon gene density has generated runs homozygosity ROH 698 gene genotypic ROH effector was fixed NA1 An ROH affected same scaffold EU1 an MR hot a between EU1 with ROH that they differ aggressiveness not by all had signatures accelerated were gene transposon There was a striking gene all effector EU2 genome was % RxLR 18 % Crinkler effector gene with % gene We that ramorum are diverging a genome that asexual are not clonal but genotypic by MR

priming can be primed for more to or a Priming follows microbe or pathogen effector or colonisation by microbe can be by treatment with some or synthetic compounds wounding primed mobilization is with tolerance phenomenon has been for decades priming is Here I unravelling priming that is dormant mitogen protein kinase chromatin modifications

Pto to speck tomato Pto gene tomato to Pseudomonas syringae tomato causative speck Pto was introgressed from a tomato into cultivated tomato over 60 ago is to speck Pto gene that it a cytoplasmically serine threonine protein kinase this pathosystem is Pto kinase with Pseudomonas effector protein AvrPto AvrPtoB Upon AvrPto or AvrPtoB Pto kinase concert with Prf a leucine protein to There has been Pto gene details about how Pto kinase with AvrPto AvrPtoB steps Pto by Pto on this will on how Pto kinase with  effector protein defining ultimately determining which are most for inhibiting   pathogen   symptoms speck

Erwinia amylovora pathogen Erwinia amylovora as some Rosaceous apple pear quince raspberry ornamentals has mushroomed last quarter On gene a Hrp gene enzyme polysaccharides gene amylovora its have been Hrp delimited by gene gene is composed hrp/hrc HEE Hrp effector elicitor HAE Hrp enzyme IT Hrp a Hrp TTSS which protein from bacterium to apoplasts or amylovora exopolysaccharides amylovoran levan which characteristic wilting symptom other gene their protein have been as amylovora that enzyme sorbitol iron harvesting This our gene gene amylovora that are

effector Pm3 wheat to powdery mildew cereals mildew gene as for wheat Triticum aestivum Triticum turgidum Pm3 agronomically to powdery mildew Blumeria graminis has been wheat but is about gene powdery mildew Here we dissected for Pm3 that Avr with a locus_1 all AvrPm3 Pm3 We effector gene AvrPm3 a2/f2 from locus_2 which is by Pm3a Pm3f a Pm3 Nicotiana benthamiana wheat Gene Bcg1 by locus_1 AvrPm3 a2/f2 between that to protein a We a for an effector a gene a pathogen a simple controls on pathogen side is more allowing flexible to gene

MLA receptor powdery mildew AVR a effector argue for a Nucleotide leucine NLR protein animal pathogen NLR pathogen effector to barley Mla has undergone NLR each detecting a effector AVRA pathogen Blumeria graminis hordei Bgh We here Bgh AVR a AVR a9 AVR a10 AVR a22 which protein by MLA MLA9 MLA10 MLA22 receptor These effector are except for AVR a10 AVR a22 that are maintained pathogen a balanced Contrary to examples effector by NLR with Mla AVR a effector by MLA receptor

modes     against pathogen  effector  are by   pathogen  effector Microbe MAMP jasmonic acid JA /ethylene ET salicylic acid SA are antagonistic for against pathogen their precise have not been clear We constructed an Arabidopsis dde2/ein2/pad4/sid2 quadruple DDE2 EIN2 SID2 are JA ET SA pad4 affects SA sector a sector by  effector AvrRpt2 AvrRpt2 by MAMP flg22 flg22 were intact with any these gene they were abolished quadruple For purposes this AvrRpt2 flg22 were measured as relative Pseudomonas syringae bacterium to   pathogen  Alternaria brassicicola was compromised quadruple measurements all coinatorial allowed us to estimate gene their on by fitting a mixed linear This allocation that contrary to ideas each JA ET SA can positively to against both pathogen that while flg22 AvrRpt2 a overlapping they is very synergistic are evident which amplify compensatory dominate explaining robustness against

NLR animal so so animal NLR receptor perceives to pathogen their protein animal NLR are normally by microbe or    NLR   pathogen  effector    NLR  effector  or effector modifications protein latter for as as    NLR maximize to through a finite immunoreceptors We into NLR initiation through homotypic receptor compare those with NLR animal

NLR join TLRs as sensor pathogen pathogen receptor PRR are are  effector  adaptive their vertebrates Toll receptor TLRs have that has their significance facets our What makes TLRs so fascinating is their to microbe that alert this we a PRR NACHT LRR NLR which both nucleotide oligomerization NODS NALPs [NACHT LRR pyrin PYD protein] underline some intriguing between NLR TLRs that emphasize NLR as a complementary for microbe

into rice against pathogen Rice feeds more than Rice by pathogen Magnaporthe oryzae by pathogen Xanthomonas oryzae oryzae are constraints to rice Genome has to pathogen PAMP effector both pathogen as as effector receptor rice these effector gene has insight into Some such as oryzae TAL effector to rice are being for genome This that govern rice

MLA receptor   are by receptor that   pathogen PAMP or by R protein   pathogen  effector We that barley mildew A MLA R protein to against powdery mildew fungus A10 effector by MLA10 associations between receptor WRKY WRKY protein as repressors PAMP MLA to with WRKY repressor repressing PAMP Our a by which these receptor integrate   pathogen

guards baits traps pathogen by effector sensor effector is conferred by dominant R gene which predominantly nucleotide leucine protein NLR against Avr gene which effector T3Es describes T3E by whereby NLR monitor protein sensor for T3E This has a framework to T3E has rationalized how can a NLR ~160 Arabidopsis to contend with a limitless effector this we a characteristic overview T3E sensor how these sensor convey T3Es to NLR protein to

PAMP effector Blurred Dichotomy pathogen PAMP are to be microbe to to effector are or to pathogen Both can PAMP effector not all activators conform to distinction between PAMP effector For some effector while some PAMP are narrowly or to pathogen As effector PAMP be for cannot exclusively be to by terms we put forward that distinction between PAMP effector between PAMP receptor protein between cannot strictly be maintained as illustrated by examples here there is a continuum between We argue that is by receptor that appropriate ligands to amplitude which is by for

Oh places they ll go A survey phytopathogen effector their phytopathogen translocate effector protein into where they sabotage to An pathogen can translocate effector during to an macromolecules protein metabolite DNA etc them a enzymatic this we have surveyed for effector them to convey carried out by protein we have locations effector as as their these phytopathogen This validates previous observations about effector immunosuppression highlights some interesting regarding effector as as phytopathogen

Os8N3 is a gene for rice on effector gene pathogen gene gene are Here we that rice gene Os8N3 a MtN3 gene from animal is elevated upon by Xanthomonas oryzae oryzae PXO99 A on effector gene pthXo1 Os8N3 resides near xa13 PXO99A failed to Os8N3 rice with xa13 Os8N3 by inhibitory RNA that were to by PXO99A to other pathogen effector gene avrXa from PXO86 enabled PXO99 A compatibility on xa13 or Os8N3 silenced that Os8N3 is a gene for by effector PthXo1 that oryzae oryzae commandeers otherwise developmentally gene to a a which pathogen a

bacterium Plasmodesmata to Invade Surrounding A hallmark multicellular is their to maintain by communicating organs is on plasmodesmata which are lined channels connecting adjacent Upon stimulation as their Here we that pathogen Pseudomonas syringae deploys an effector protein HopO1 that HopO1 is for syringae to spread locally to neighboring during HopO1 Arabidopsis Arabidopsis thaliana distance flux between neighboring Being a ribosyltransferase catalytic HopO1 is for HopO1 with destabilizes protein LP LP5 Both LPs are Our that a bacterium utilizes an effector to for maximizing spread Pseudomonas syringae effector protein HopO1 destabilizes plasmodesmata protein to

Pathogenomics Ralstonia solanacearum Ralstonia solanacearum is a phytopathogen that attacks other over a geographical for has to an R solanacearum Genome more than representative main groups has broadened our this  pathogen   to are opening for refined are described we their how their is tightly by an intricate

a further additions for multi pathogen pathogen is at www org expertly on gene proven to outcome pathogen peer reviewed articles that gene that did not are to datasets for purposes virus are not included Here we a revised platform with search filtering extended A PHIB search is a link to PHI Canto a for authors to curate their own into October 2016 has an from 2219 manually references on 4460 gene from 264 pathogen on 16 8046 Prokaryotic pathogen are represented almost equal nuers ∼ % to % to other medical included into are pathogen effector protein curation problems encountered directions project are briefly

pathogen pathogen is at www org expertly on gene proven to outcome pathogen peer reviewed articles curates describing gene that did not to datasets for purposes virus are not included to their coverage other databases this we plus further with complementary databases Septeer 2019 3454 manually references on 680 gene from 268 pathogen on 210 801 Prokaryotic pathogen are represented almost equal nuers consist approximately 60% split 50 50 between cereal cereal 40% other medical on pathogen effector has risen by more than a entries for pathogen that infect has dramatically this We briefly direction project some existing problems with curation

between RRS1 R a protein conferring to PopP2 a effector to RRS1 R to Ralstonia solanacearum as recessive this R gene a protein whose coines TIR NBS LRR R protein a WRKY characteristic some behaves as a dominant gene Here we that PopP2 a R solanacearum effector which belongs to YopJ/AvrRxv protein is protein by RRS1 R an between PopP2 both RRS1 R RRS1 Nd Col Arabidopsis thaliana ecotypes was by yeast split ubiquitin hybrid This which R protein was not between RRS1 protein PopP1 YopJ/AvrRxv GMI1000 that Petunia We further that both Avr protein RRS1 protein colocalize that RRS1 protein are on PopP2

Phytopathogen effector Subverting Foes Battleground bacterium fungus oomycete invade their through routes These pathogen groups  effector protein that aid this a   pathogen   has by effector from   pathogen  groups by receptor phytohormone or with secretory   pathogen  penetrations     some against pathogen These are by effector that a are by completely   pathogen  groups that these are a battleground encountered by     pathogen

Phytophthora genome uncover origins Draft genome have been for soybean  pathogen  Phytophthora sojae sudden oak   pathogen  Phytophthora ramorum oomycete such as these Phytophthora Stramenopila with photosynthetic algae such as diatoms Phytophthora gene probable phototroph supports a photosynthetic ancestry for stramenopiles genome a protein with    such as hydrolases ABC transporter protein toxin proteinase inhibitors a 00 protein with to oomycete gene

Phytophthora infestans     R gene destroyer Phytophthora infestans a problem to Historically there have been controversies concerning its some which today promise to fascinating insight into its its genome as abundance effector means that this oomycete a formidable foe

Phytotoxic metabolite peptide by Dothideomycete fungus fungus belonging to Dothideomycetes phytotoxic metabolite peptide that are for Phytotoxins that a are as toxin HST HST only a or more genotypes that For pathogen producing HST are by to toxin while is on toxin HST are not main but to producing pathogen Dothideomycetes are for toxin HST because they are only so to them most HST HST will be on HST have highlighted programed as an We HST upon that this respect nucleotide leucine protein against biotrophs they can to necrotrophs This paper describes toxin HST HST by belonging to Dothideomycetes

  protein NBS LRR protein their ners Most    R protein a leucine LRR a nucleotide NBS a amino They are NBS LRR protein LRR a protein from serve as protein platforms as modules protein LRR    R protein are their can to    familiar A 149 R gene are Arabidopsis genome     must deal with difficult task asseling protein by these gene into to deal with this problem protein are spatially restricted to their improving probability that they will with their proper ners these are architecturally organized to avoid inappropriate to maintain fidelity efficiency when it is initiated into how R protein be created managed held check until stimulation Nevertheless R protein ners these that have been to date are unclear

  by  pathogen refined   is by complementary gene from   gene from   pathogen This has been interpreted as a receptor ligand which protein to protein which initiates    that protein is for their there is accumulating that protein are not receptor for protein We our examine validity receptor ligand other context most

  Advancing on frontiers have to cope with pathogen which that on receptor takes Exciting breakthroughs were on receptor PRR nucleotide NBS leucine LRR receptor NLR This PRR emphasizing most discoveries about PRR’ to mitogen protein kinase MAPK calcium Ca2+ have NLR to perceive pathogen effector more We NLR which has been greatly advanced by breakthroughs NLR NLR sensor helper Toll/interleukin receptor TIR

against virus receptor limitations that virus serious threats to virus have to an for engineered during Nevertheless a constant that has to a continuing interest virus that or This latest breakthroughs against virus attention is given to receptor with a that reseles which is broadly divided into pathogen PAMP effector An virus on translation by a receptor all virus by during is for these conversely virus to circumvent through RNA by utilizing RNA which are by virus Additionally defend themselves against virus through hormone ubiquitin 26S proteasome UPS which alternatively impairs facilitates exist hence is on extent at which these opposing emerge A deeper innovative biotechnological for improvement

an pathogen are engaged a continuous struggle for dominance with their pathogen outcomes these are to human as they can have dramatic on convergence pathogen is an picture pathogen from both have an amazing to pathogen through both pathogen elicitor pathogen through effector These biotechnological to

    pathogen have to recognise reject pathogen at stages their attempted colonisation   Nonspecific rejection arises as a consequence   pathogen attempt to breach     pathogen to penetrate beyond this barrier seek a subtle persuasive with   For some this be to outside     but for it to     or these Our its to has advanced as more is about underpinning both kinds

NBS LRR protein pathogen protein belonging to nucleotide leucine NBS LRR are for pathogen Nod LRR protein sensor that pathogen NBS LRR protein pathogen protein most effector pathogen for protein are by NBS LRR protein from modifications protein inflict on protein some NBS LRR protein pathogen protein with a protein or a pathogen protein to conformational amino LRR NBS LRR protein Such conformational are to exchange ADP for ATP by NBS which by an to pathogen

NBS LRR protein adaptable guards gene nucleotide leucine NBS LRR protein This is by gene per genome can be subdivided into TIR TNL CC CNL subfamilies Their precise is they are to monitor status protein that are by pathogen effector

receptor FLS2 is directed for by ubiquitin ligase AvrPtoB "Background An is pathogen PAMP by receptor For pathogen on to PAMP disable to PAMP receptor PRR are prime for pathogen effector FLS2 its coreceptor BAK1 EFR receptor kinase that a against pathogen Here we that Pseudomonas syringae tomato DC3000 PtoDC3000 Arabidopsis is through its effector AvrPtoB which FLS2 We that AvrPtoB through its associates with FLS2 BAK1 which with FLS2 is by flg22 AvrPtoB is as an E3 ligase to catalyze polyubiquitination kinase FLS2 a enhancement PtoDC3000 to require E3 ligase AvrPtoB Conclusions AvrPtoB initially through its tomato Pto kinase is composed at is for with Pto an E3 ligase on our we that both AvrPtoB to PtoDC3000 Arabidopsis through their to eliminate FLS2 from periphery other PAMP sensor that are constitutively or after pathogen "

parasite Bridging gap between ecology We ideas about parasites that generate Frequencies parasite that GFG coevolve familiar boom bust which is selected when is rare is selected when is can stable when epidemiological frequency ndFDS on parasite or both such that a trait to declines as its frequency can be stabilized by overdominance when heterozygous have greater than homozygotes to pathogen can persist statistical sustained by random acting on gene frequencies Stable to be lived to be detectable promoting stability parasite have been lost to parasite effector

pathogen effector probes interfering with spatial temporal manners arsenals receptor capable all pathogen To must be to or derive from Consequently to some these pathogen effector protein that This covers effector on by effector from pathogen effector to pathogen into interior for pathogen is pathogen effector a spatial temporal on stage have our effector acting organs effector are excellent probes that insight into as as vulnerability

pathogen warfare under climate by human have accelerated past 200 greenhouse gases is to continue to raise temperature change water 21st this we explore profound has on — a will not be by a pathogen if are not conducive for change CO2 concentrations temperature water can have neutral or on as each differently to these variations optima could apply to all to effector RNA hormone are all affected by On pathogen side such as toxin protein as as pathogen survival are influenced by temperature For practical reasons most laboratory investigations into pathogen at on a few static that capture only a fraction pathogen that There is need for to increasingly to fully multidimensional pathogen that are resilient to climate change Velázquez et al how climates how their pathogen

Port translocon animal bacterium a TTSS as a syringe to effector protein into Protein is by a translocon this are from being pathogen animal 111 translocon protein TTPs have been Interestingly TTP are not there are such as segments coiled coil Accumulating that TTPs are oligomeric protein channels that are inserted into by TTSS

Guidelines for a Unified Nomenclature Hop effector protein pathogen Pseudomonas syringae Pseudomonas syringae with their Hrp outer protein Hop effector protein injected into by TTSS genome for a syringae has to a effector a nomenclature has names being assigned to same Hop Hop by same alphabetic character failure name choices to standards confirmation or relatedness bioinformatic are for protein to be as Hop A generic Hop name HopXY# is wherein meership is indicated by alphabetic characters subgroup meership numerically source subscript Guidelines are for name for Hop that are to Hop chimeras pseudogenes truncations or nonexpressed Hop are described which have been to guide their into nomenclature

protease pathogen a to to create suitable for their propagation have gene whose as a how a effector to on but becomes an that on has been a have that a list pathogen effector as protease that are into to modify protein protease have been to These that translational protein through processing is a

Pseudomonas syringae effector AvrPto by receptor kinase receptor kinase such as FLS2 EFR to perceive pathogen This is by effector allowing pathogen propagation To have gene that effector reinstate Pseudomonas syringae effector AvrPto but protein kinase Pto protein Prf Here we that AvrPto receptor kinase Arabidopsis FLS2 EFR tomato LeFLS2 to to receptor kinase is for AvrPto FLS2 AvrPto Pto AvrPto to requirements Pto competes with FLS2 for AvrPto that by which AvrPto is to Pto which with Prf bacterium

Pseudomonas syringae Hrp effector protein Pseudomonas syringae is a an pathogen animal that on a to effector protein into syringae hrp/hrc gene Hrp avr Hrp outer protein hop gene effector protein hrp/hrc gene syringae syringae 61 syringae syringae B28a syringae tomato DC3000 are flanked by an exchangeable effector a effector a triite mosaic Hrp Pai that is to a tRNA Leu gene Pseudomonas aeruginosa but linkage to Hrp gene Cosmid pHIR11 a portion 61 Hrp that is to Escherichia coli Pseudomonas fluorescens to HopPsyA into tobacco eliciting a normally only by pathogen deletions DC3000 that effector is for but exchangeable effector has only a minor tomato syringae HopPsyA AvrPto culture a Hrp at pH temperature with AvrPto is by Yersinia enterocolitica AvrPto on codons which are to an Npt reporter from enterocolitica that a universal is by both animal pathogen

Pseudomonas syringae tomato DC3000 A   pathogen  for Probing Hormone 1980s   pathogen  Pseudomonas syringae have been as for   1991 a syringae tomato Pst DC3000 was to infect not only its tomato but Arabidopsis laboratory a finding that spurred intensive efforts decades to by which this that Pst DC3000 a proteinaceous effector that are through a polyketide phytotoxin coronatine which structurally mimics    hormone jasmonate JA Pst DC3000 has not only how a   pathogen  employs effector to     but has facilitated stomata JA from Pst DC3000 prove useful other    pathogen

Pseudomonas syringae tomato hijacks Arabidopsis abscisic acid to We have that a for effector by Pseudomonas syringae is abscisic acid ABA Microarray a prominent effector gene that were with ABA to this hormone gene upregulated by effector a 42% overlap with ABA responsive gene are by osmotic drought were NCED3 a enzyme ABA abscisic acid insensitive ABI1 clade gene protein phosphatases 2C PP2Cs ABA PP2C ABA insensitivity or hypersensitivity to restriction or multiplication bacterium ABA during colonisation Exogenous ABA application colonisation was an ABA biosynthetic effector AvrPtoB ABA Our that a is effector manipulation hormone which to

Pseudomonas syringae effector Last words endless arguments pathogen by injecting compositionally but effector protein pathogen Pseudomonas syringae is a for exploring such pangenome syringae effector injected by effector gene a effector vesicle trafficking a larger effector kinase disassely 28 effector a reassely a minimal simultaneously attacking both These observations coupled with effector a for coevolving dialogs between effector that emphasizes mutually governing

Pseudomonas  effector AvrPtoB     by AvrPtoB  effector protein is genera     pathogen it an Here we that Pseudomonas AvrPtoB     to initiated by Pto Cf9 protein pro apoptotic mouse protein Bax AvrPtoB yeast that AvrPtoB as a inhibitor kingdoms truncated AvrPtoB protein we AvrPtoB that are for We a Rsb that is by an AvrPtoB truncation disrupted A Pseudomonas syringae tomato DC3000 with a chromosomal AvrPtoB elicited Rsb tomato was restored when AvrPtoB was our that a  effector can     to by inhibiting

R gene by a  effector  rice R gene that recognise incompatible pathogen a to   R gene is dictated by both R gene avr gene pathogen is not Here we R gene Xa2 from rice avr gene avrXa2 from Xanthomonas oryzae oryzae Xa2 identical protein only when a rice   is challenged by bacterium harbouring avrXa2 whose is a  effector Xa2 only vicinity Xa2 to otherwise compatible   pathogen Xa2 incompatible pathogen differential R gene AvrXa2 effector

microbe a at timescales is a hallmark microbe for rests on genome interacting ners Here we into characteristics that both phytopathogen These fresh gene pathogen effector recognised RNA warfare metabolite we permissive ploidy on We that means microbe are multifaceted on each

oxygen fungus oxygen ROS a pathogen ofa pathogen by which is for further reactions ROS pathogen is unclear on pathogen so have focused on OSR to or its avoidance by effector This focuses on ROS for it has obvious that a fungalOSRsystemsmightnot have pathogen especially necrotrophs can actively to ROS take advantage ofthe ’ fungus superoxide generatingNADPH oxidases to Nox that are for that they are not pathogen but differentiation that are for

RXLR effector Some most oomycete pathogen effector protein with signature amino acid RXLR that to on RXLR effector has been very over decade that has transpired their that RXLR gene a for Phytophthora downy mildew Importantly against these oomycete is on RXLR protein have through which this can be broken most RXLR gene have a that is RXLR protein a foundation for detailed Finally have that is a for RXLR protein protein are being a Some hubs that are manipulated by bacterium fungus vulnerability

PAMP effector against Rice Fungus Magnaporthe oryzae Rice by pathogen Magnaporthe oryzae is most rice rice oryzae pathosystem has a because its scientific advancement have a pathogen PAMP effector from fungus that rice upon between effector their protein have into this we on those gene both oryzae rice that are for PAMP effector We directions for that will further our this pathosystem

Receptor kinase pathogen more than Receptor kinase RLK Receptor protein RLPs a RLK RLPs as receptor PRR that microbe as inducible have PRR their ligands PRR PRR with other RLK as multiprotein at Innovative have for their PRR pathogen can or by secreting effector protein to “hide” or PRR newly pathogen effector have been to RLK by mimicking peptide hormone ongoing RLK

Receptor kinase Integrate from receptor Are by a Pseudomonas syringae effector receptor such as FLS2 pathogen PAMP PAMP through Pseudomonas syringae effector AvrPphB a cysteine protease cleaves Arabidopsis receptor kinase PBS1 to receptor RPS5 specified effector Analyzing AvrPphB RPS5 we that AvrPphB can by cleaving PBS1 PBL kinase BIK1 PBL1 PBL2 unstimulated BIK1 PBL1 with FLS2 are upon FLS2 by its ligand flg22 that BIK1 PBL1 PBL2 PBS1 integrate from receptor AvrPphB these kinase PBS1 is monitored by RPS5 to this effector other PBL kinase for

Reconstitution a NLR resistosome conferring Nucleotide leucine receptor NLR perceive pathogen effector to NLR have until We reconstituted an Arabidopsis coiled coil NLR ZAR1 pseudokinase RKS1 uridylated protein kinase PBL2 ′ deoxyadenosine ′ triphosphate dATP oligomerization during cryo electron microscopy a wheel pentameric ZAR1 resistosome nucleotide coiled coil ZAR1 to resistosome pentamerization by forming an α helical barrel that with leucine winged remodeling switching during very amphipathic α ZAR1 to a funnel shaped that is for triggering offering to a resistosome

Xanthomonas bacterium Xanthomonas a monocotyledonous dicotyledonous multiplication on adhesins polysaccharides LPS degradative enzyme is which injects effector protein into to such as to pathogen coordinated is orchestrated by quorum transcrip tional regulators such as Clp Zur FhrR HrpX HpaR gene is by RNA protein RsmA this we on from Xanthomonas

RIN4 with Pseudomonas syringae effector is for RPM1 Arabidopsis Arabidopsis RPM1 against Pseudomonas syringae effector AvrRpm1 or AvrB An RPM1 interacting protein RIN4 coimmunoprecipitates from extracts with AvrB AvrRpm1 or RPM1 Reduction RIN4 protein both restriction pathogen by RPM1 RIN4 reduction diminution RPM1 RIN4 reduction heightened to Peronospora parasitica syringae gene RIN4 positively regulates RPM1 is formally a AvrRpm1 AvrB RIN4 This enhance RIN4 as a pathogen RPM1 "" against pathogen that AvrRpm1 AvrB to RIN4

RXLR effector reservoir Phytophthora is dominated by a with more than 00 pathogen effector that their A effector Phytophthora RXLR EER for these effector into Here we bioinformatically >30 effector gene each genome sojae ramorum A homolog Avh gene accounts for most effector Avh protein but are all from a ancestor by More than Avh protein that are arranged as a module that can be repeated up to eight times Avh gene to most genome they are nearly always at synteny breaks all oomycete effector gene its pace is with a for Avh protein with

Salicylic acid to Salicylic acid SA has as a hormone with Arabidopsis pathogen SA on SA insensitive to SA receptor how SA regulates gene with its SA is for a normal root microbiome pathogen effector have to SA or This discusses SA on SA SA

protein from Ralstonia solanacearum a hundred tricks to kill a genome has a tremendous for protein This is certainly true for pathogen Ralstonia solanacearum which is estimated to protein through protein to are II both which serve to effector conservation effector into are starting to be unravelled into on GALA PopP2 protein how R solanacearum effector by mimicking protein or by inducing their relocalization

constantly have to face pathogen attacks rarely to possessed by pathogen are perceived by recog nition that so effector both which are accompanied by a that repel pathogen attacks Here we occurring during on mitogen protein kinase

Against pathogen frontline against pathogen such as bacterium fungus oomycete is by receptor that pathogen PAMP to To this pathogen effector protein with a to have R protein detecting pathogen effector to effector an amplified with case virus no PAMP was so is to be mainly on RNA an that regulates gene chromatin states represses nucleic acid such as transposon generate 21 24 nt RNA miRNAs short interfering si RNA that repress gene Dicer DCL protein which normally miRNAs siRNA all to biogenesis siRNA that RNA to against pathogen Conversely to R protein same NB LRR are to both effector Avr protein RNA virus as a virus some Avr protein as We hypothesize that RNA concert to fight virus virus this dual by effector that both / RNA to

MAMP upstream MAPKKK Arabidopsis animal to infections are effectively "nonhosts" for most pathogen Arabidopsis /nonpathogenic Pseudomonas syringae sustains but syringae MAMP microbe marker gene We performed a AvrPto AvrPtoB as potent gene kinase MAPK Unlike effector pathogen AvrPto AvrPtoB intercept MAMP upstream MAPKKK at to receptor Arabidopsis AvrPto MAMP syringae Deletions avrPto avrPtoB from syringae its a MAMP a effector from bacterium

Stacking gene from into African highland potato to for million deaths Ireland continent during 1840s by Phytophthora infestans most potato Solanum tuberosum with about % % annual yield Saharan Africa mainly smallholder farmers We here that R gene from relatives [RB Rpi blb2 from Solanum bulbocastanum Rpi vnt1 from venturii] into potato over seasons We that stacking R gene a frequency with to with 3R gene stack from potato Desiree Victoria grew normally showing pathogen any fungicide spray their equivalent were killed Characteristics pathogen that to be lasting because it has essentially consists 2_A1 which expresses effector gene Yields from Desiree Victoria grown fungicide to scale farm holders were estimated to be 29 45 /ha This a to over national average these potato which are farmers preferred could be adopted bring income to smallholder farmers Saharan Africa

sexual wheat pathogen effector AvrStb6 strobilurin fungicide treatments are cornerstones Here we that these treatments sex parenthood wheat pathogen Zymoseptoria tritici We that tritici wheat complies with by effector AvrStb6 which is by wheat protein Stb6 implying removal avirulent from pathogen tritici crosses on wheat that sex with an avirulent parent are retained Crossing fungicide sensitive fungicide under fungicide pressure a frequency under always as male donors parenthood Modeling these observations for extended durability fungicide sex has implications for

a pathogen TE are drivers genome landscape genome is a inducing TE are pathogen are excellent to dissect on TE for pathogen i effector TE To dissect TE to we analyzed TE landscape wheat pathogen Zymoseptoria tritici We exposed to starvation Contrary to expectations we that TE most TE miniature inverted Gypsy Both context TE background i harboring same TE were predictors under Gene profiles under varied on proximity to closest TE against TE were ineffective to Next we analyzed Avr3D1 effector We that insertion TE proximity contributed to on a wheat cultivar complexity TE responsiveness to backgrounds locations substantial intraspecific to TE with consequences for

for a heterodimeric receptor receptor pathogen effector protein effector Arabidopsis receptor RPS4 RRS1 are both to to pathogen We that RPS4 RRS1 Crystal Toll interleukin receptor/ TIR RPS4 RRS1 individually as a heterodimeric at 05 65 angstrom resolution a TIR/TIR We that TIR heterodimerization is to a RRS1/RPS4 effector RPS4 TIR effector which is inhibited by RRS1 TIR through heterodimerization RPS4 RRS1 as a receptor which

barley NLR receptor MLA10 its receptor a multi protein reseling animal NOD receptor NLR NLR pathogen effector by AVR gene with barley MLA gene is NLR coiled coil CC NB LRR that each detects a effector barley powdery mildew fungus We MLA10 barley Nicotiana benthamiana MLA10 CC is by CC NB ARC by LRR receptor Enforced MLA10 by tagging with a NLS or a NES that MLA10 is but By MLA10 is to against powdery mildew fungus MLA10 retention was achieved through attachment a glucocorticoid receptor hormone GR by which we reinforced MLA10 with our showing an MLA10 this a bifurcation MLA10 a

by effector beyond Most pathogen employ a to effector T3E protein These T3Es to create a permissive niche for allowing An T3Es bacterium is has T3E from modulation hormone or organelle This insight discusses T3E other than which to modulation to survival replication dissemination Editorial by Lennon

Sugar transporter for exchange nutrition pathogen Sugar transporter are for maintenance animal blood glucose nectar seed pollen identity sugar transporter has elusive optical glucose sensor we a sugar transporter named SWEETs that at out teen Arabidopsis out over twenty rice out Caenorhabditis elegans copy human protein glucose Arabidopsis SWEET8 is for pollen viability rice SWEET11 SWEET14 are by pathogen for by means a effector to SWEET promoter symbiont pathogen SWEET gene that sugar SWEET transporter is by pathogen symbiont for nutritional metazoan be sugar from intestinal liver epididymis mammary

gene by a effector consists layers governs fends off most attempted invasions is on R gene that effector protein by pathogen to or Here we that a fungus an effector that can both R gene This effector Avr1 is by xylem fungus Fusarium oxysporum lycopersici Fol when tomato a R gene I or I At same time Avr1 protective other R gene I I on these observations we tentatively reconstruct that has taken place between tomato R gene effector Fol This has a hitherto unpredicted for durable on gene coinations

receptor by effector During microbe are by receptor PRR to an that prevents ingress pathogen must or PRR to past decade a effector T3Es protein from bacterium have been to this More detailed have been for these effector Interestingly effector a being to receptor to hijack being a fascinating pathogen effector have as to dissect

Ten things to know about oomycete effector intractable by standards oomycete have moved to centre stage on microbe on oomycete effector trafficking has to science pathology this we a historical on oomycete art effector oomycete by describing what we consider to be most about oomycete effector

AvrPm3 Pm3 effector NLR both cereal mildew on wheat wheat Pm3 gene against powdery mildew pathogen as an receptor which upon AVR effector from pathogen Here we effector protein from mildew pathogen wheat rye grass Dactylis glomerata by PM3B PM3C PM3D receptor with earlier AVRPM3 A2/F2 AVRs PM3B/ AVRPM3 B2/C2 PM3D AVRPM3 D3 to a protein with but AvrPm3 b2/c2 AvrPm3 d3 are all wheat rye mildew that Pm3b Pm3c Pm3d are restricting rye mildew on wheat divergent AVR from rye Dactylis mildew are by PM3B PM3C or PM3D their

effector HopX1 JAZ Repressors to Jasmonate Arabidopsis Pseudomonas syringae is on a which a suite effector protein into a toxin such as coronatine COR which is a hormone jasmonate isoleuce JA Ile effector to disrupt fact that effector is to effector Here we that effector HopX1 from Pseudomonas syringae tabaci Pta 11528 a that does not COR with JAZ protein a JA repressors We that hopX1 a cysteine protease that is for JAZs by HopX1 HopX1 associates with JAZ protein through its ZIM a COI1 Moreover HopX1 Arabidopsis JA gene represses salicylic acid SA complements a COR deficient syringae tomato Pto DC3000 during infections HopX1 promoted when by to a extent as COR this was on its catalytic Altogether our that JAZ protein are effector to JA Arabidopsis HopX1 illustrates a paradigm an alternative solution to COR with outcome

birth effector pathogen genome fungus oomycete are risks to to their overcoming pathogen effector to with These effector are genome We how effector gene were lost pathogen genome drawing on between effector chromosomal rearrangements Some effector entered pathogen genome or introgression effector arose through gene or from coding an effector is tightly to its during Some effector converged on an imposed by against were drivers effector that to mosaics effector Such effector mosaics was foundation for pathogen

Phytophthora infestans RXLR effector AVR3a is to R3a hypersensitivity INF1 Nicotiana benthamiana RXLR effector AVR3a Phytophthora infestans on potato R3a gene Avr3a protein that differ only amino acid which are mature protein Avirulent Avr3a which AVR3a KI amino acid K 80 I 103 express only avr3a AVR3a EM 80 103 Only AVR3a KI protein is where it R3a hypersensitivity to other oomycete protein AVR3a KI a peptide followed by a centered on consensus RXLR that is to a malaria parasites between Avr3a R3a can be reconstructed by their Nicotiana benthamiana We benthamiana to further Avr3a R3a R3a by AVR3a KI is on ubiquitin ligase protein SGT1 heat shock protein HSP90 AVR3a KI AVR3a EM protein are equally stable that difference R3a cannot be attributed to AVR3a EM protein instability AVR3a KI is to by elicitin INF1 infestans a for this protein indicated that amino acid AVR3a KI which excludes RXLR is for with that AVR3a KI other RXLR effector is but is not for effector We that both amino acid K 80 I 103 mature AVR3a to effector

genome Arabidopsis tomato pathogen Pseudomonas syringae tomato DC3000 We genome pathogen Pseudomonas syringae tomato DC3000 DC3000 which is on tomato Arabidopsis thaliana DC3000 genome megabases a circular chromosome plasmids which 63 We 298 gene gene 31 effector protein gene were paralogous were proximal to mobile which % DC3000 genome bacterium possesses a transporter for acquisition sugars as as gene attachment to surfaces Over % gene are dedicated to which need for to encountered during epiphytic a degree with pseudomonads Pseudomonas putida Pseudomonas aeruginosa 159 gene to DC3000 which 811 a

between jasmonic acid other hormone jasmonic acid as a to a hormone developmental to a On hand hormone allocate resources to most serious on other hand hormone balance between have between jasmonic acid JA other hormone on these this paper mainly reviews between JA other hormone balance between protein JASMONATE ZIM PROTEIN JAZ MYC2 as are highlighted We that JA with other hormone [such as auxin ethylene ET abscisic acid ABA salicylic acid SA brassinosteroids BRs gibberellin GA ] to a tolerance against pathogen such as Magnaporthe oryzae Pseudomonas syringae JA as a phytohormone

Crystal TAL effector PthXo1 Bound to Its DNA DNA by TAL effector is by tandem each 33 to 35 that specify nucleotides diresidues RVDs crystal PthXo1 bound to its DNA was by by heavy atom derivatization Each a left handed bundle that presents an RVD loop to DNA to a right handed superhelix wrapped around DNA groove RVD a stabilizing contact with protein backbone while makes a base contact to DNA sense strand degenerate amino with DNA RVDs noncanonical associations illustrates TAL effector DNA

genome     pathogen Cladosporium fulvum Dothistroma septosporum to But Signatures Ancestry We genome Dothideomycete pathogen Cladosporium fulvum syn Passalora fulva Dothistroma septosporum Dse that are but have both fungus grow extracellularly contact with mesophyll is a biotroph tomato while Dse is a hemibiotroph pine genome these fungus have a gene % gene both genome are but differ >61 Dse 31 which is mainly to difference 4 % versus % Dse to is suggested by diverged gene an alpha tomatinase gene that we predict be for detoxification tomatine while this gene is absent Dse gene protein are to each areas are for these gene gene ancestry effector gene Ecp2 Avr4 are Dse a Cf Ecp2 Cf Strikingly gene toxin dothistromin a for Dse are but their differs markedly with essentially no by Likewise has a carbohydrate degrading enzyme catalog that is more to that necrotrophs or hemibiotrophs a larger pectinolytic gene than Dse but these gene are not or are pseudogenized their genome that these pathogen had a ancestral but to by a coination differentiated gene pseudogenization gene

nonobligate biotrophs is a pervasive trait that fungus oomycete pathogen convergences gene inorganic nitrogen inorganic sulfur thiamine gene carbohydrate enzyme enzyme Some biotrophs but not all marked genome because a retrotransposons I argue here that constraints on transposon is by advantages conferred by variability that from transposition by creation palettes effector gene genome gene are consequences this trade off gene that are not for on a disappeared but we do not know what lost make some these pathogen

Magnaporthe oryzae effector AvrPiz RING E3 Ubiquitin Ligase APIP6 to pathogen Rice a few effector protein by oomycete pathogen have been elucidated for pathogen effector is for those fungus Here we that effector AvrPiz from rice fungus Magnaporthe oryzae preferentially accumulates interfacial is then into rice Oryza sativa AvrPiz rice flg22 chitin oxygen ROS enhances to oryzae that AvrPiz to pathogen PAMP rice that AvrPiz ubiquitin ligase rice RING E3 ubiquitin ligase APIP6 that return APIP6 ubiquitinates AvrPiz Interestingly agroinfection that AvrPiz AvrPiz Interacting Protein APIP6 are both degraded when coexpressed Nicotiana benthamiana APIP6 rice to a reduction flg22 ROS gene rice to oryzae Taken our a which a effector ubiquitin proteasome for PAMP

Facets Through Secretomics secretome is frame proteomics aiming at characterizing protein their accounting for their this we aim to pertaining to through conventional unconventional protein those exosomes or vesicles are to actively a from polymers RNA DNA to compounds ATP phytochemicals metabolite phytohormone All these pivotal fungus or oomycete both for fungus deleterious outcomes pathogen for For instance that such by roots is paramount to sculpt rhizospheric microbiota Our aim this is to extend definition secretome to a broader sense to functioning /microorganism holobiont perspectives will be brought to along with that should establishing an friendly

NB‐LRR protein RGA4 RGA5 to protein nucleotide leucine protein NB LRR are sensor which pathogen AVR protein We that RGA4 RGA5 NB LRR from rice to to pathogen Magnaporthe oryzae accomplish AVR RGA4 an AVR that is repressed RGA5 both rice protoplasts Nicotiana benthamiana Upon pathogen effector AVR Pia by to RGA5 is relieved RGA4 RGA5 homo hetero through their coiled coil rice protoplast that RGA4 RGA5 localize to Upon AVR Pia neither RGA4 nor RGA5 is re to These a for hetero NB LRR RGA4 while RGA5 as a repressor RGA4 as an AVR receptor

NLR Annotator Receptor gene nucleotide leucine NLR receptor protein pathogen by pathogen effector genome NLR gene hexaploid wheat Triticum aestivum cultivar Chinese Spring genome a detailed its NLR complement NLR intrafamily hinder their accurate Here we NLR Annotator a software for silico NLR transcript for wheat we universal applicability NLR Annotator taxa We applied our to wheat it with a transcript subset gene from gene to phylogeny profile NLR gene We 400 NLR which 560 were as gene with intact open frames NLR with subclades subclade predominantly locate proximity to NLR a paired helper Most NLR 88% lower percentile transcripts young subjected to we up 266 NLR To utility our for positional gene we estimated NLR gene intervals mapped rust gene Our will gene wheat to

pan genome effector landscape a pathogen effector by receptor to effector protects against pathogen a prevalence against pathogen is We constructed Pseudomonas syringae effector Compendium PsyTEC to pan genome complexity 512 effector protein distributed from 494 to 529 representative We screened PsyTEC on Arabidopsis thaliana 59 eliciting 11 % from 2 % with orthologs distributed 96 % syringae We undescribed receptor CAR1 which effector AvrE HopAA1 that 94 % to be by CAR1 or ZAR1

pathogen additions developments pathogen a epidemics that threaten yield as as human animal ecosystem health To coat greater is on pathogen catalogues verified effector gene from protist pathogen are with gene included pathogen infect a humans animal insect fish other fungus at http //www org stores on 285 gene 4102 110 160 103 54 animal 181 drawn from 1243 references Phenotypic gene has been by manual curation peer reviewed A vocabulary consisting terms permits comparisons taxonomic were mapped their gene to genome Ensel genome gene hotspots can be visualized genome browsers plans for community curation inclusion

  microbe are pathogen that impair    to a branched branch responds to to microbe pathogen responds to  pathogen   or through their on These      pathogen   to which they extraordinary into kingdoms A detailed     will underpin improvement for fibre biofuels

Pseudomonas syringae Hrp has a triite mosaic composed a gene bounded by exchangeable effector effector that to bacterium Pseudomonas syringae is divided into differing with syringae syringae Psy syringae tomato Pto representing divergent syringae hrp/hrc gene a protein that to translocate Avr Hop effector protein into DNA hrp/hrc Psy 61 Psy B28a Pto DC3000 has a Hrp Pai with a triite mosaic hrp/hrc gene is all is flanked by a exchangeable effector EEL a effector CEL EELs begin nt stop codon hrpK end after kb dissimilar intervening DNA with tRNALeu queA tgt that are Pseudomonas aeruginosa but linkage to any Hrp Pai EELs effector HopPsyA HrmA Psy 61 protein to AvrPphE AvrB/AvrC/AvrPphC AvrBsT/AvrRxv/YopJ protein Psy B28a EELs mobile have a G + lower than rest Hrp Pai or syringae genome CEL at that are between Psy B28a Pto DC3000 Deletion Pto DC3000 EEL slightly reduces tomato deletion a portion CEL reduces abolishes tomato

Pseudomonas syringae phytotoxin coronatine by overcoming salicylic acid Arabidopsis thaliana   pathogen   Pseudomonas syringae to this is coronatine COR a phytotoxin believed to as an analogue or more jasmonates a     regulators COR biosynthetic COR syringae tomato DC3000 exhibit on Arabidopsis thaliana tomato biochemically COR DC3000 were to explore COR its precursors coronafacic acid CFA coronamic acid CMA on A thaliana Inoculation with DC3000 jasmonate responsive transcripts with a that accumulates CFA which is structurally to methyl jasmonate MeJA did not COR but not CFA stimulates jasmonate during syringae A thaliana COR to grow to was fully restored A thaliana deficient for salicylic acid SA COR grew to SA deficient symptoms were these these that COR is both for overcoming or SA during     for normal symptom A thaliana

Rice Protein Pair RGA4/RGA5 Magnaporthe oryzae effector AVR Pia AVR1 CO39 by R protein pathogen Avr protein by or are multidomain protein a nucleotide NB a leucine LRR NB LRR protein coding gene from rice Oryza sativa RGA4 RGA5 were to be for Magnaporthe oryzae effector AVR1 CO39 RGA4 RGA5 oryzae effector AVR Pia that R protein dual For RGA5 alternative transcripts RGA5 A RGA5 were that only RGA5 A while RGA5 is Yeast hybrid coimmunoprecipitation fluorescence resonance energy fluorescence lifetime imaging AVR Pia AVR1 CO39 to RGA5 A for Avr protein by to a R protein seems to be for as an AVR Pia did not RGA5 A A Avr with to Avr rice R protein Pik was RGA5 A This a Avr protein through to a LRR

rice TAL effector protein XA10 calcium depletion endoplasmic reticulum between R gene their Avr gene pathogen can a our R gene Here we Xa10 a TAL effector R gene for to rice Oryza sativa Xa10 a for TAL effector AvrXa10 EBEAvrXa10 its promoter AvrXa10 Xa10 Xa10 rice Nicotiana benthamiana HeLa Xa10 gene XA10 as hexamers endoplasmic reticulum ER is with ER Ca2+ depletion HeLa XA10 that ER Ca2+ depletion benthamiana HeLa rice We that XA10 is an inducible intrinsic terminator protein that by a ER Ca2+

pathogen protein A fungus can wilting through most being Verticillium dahliae Fusarium oxysporum all microorganism fungus protein during genome proteomics screens have these protein cysteine protein necrosis inducing protein enzyme Gene deletion have that some these protein are for while other protein enigmatic On other hand can some protein or their actions We give an overview protein to be by fungus their

auxin during between pathogen their pathogen have to their to to less these is hormone hormone analogs there is that modulation hormone is during pathogen auxin indole Acetic acid IAA that auxin Auxin is for being but have that elevated IAA or auxin can some pathogen to stimulating during by gall forming bacterium auxin auxin can antagonize Auxin can as a to some pathogen this we that auxin auxin auxin on pathogen during with emphasis on impacts auxin on with pathogen

speed genome pathogen waltz with fungus oomycete deep pathogen last have seen genome a multitude these so pathogen Already have pathogen genome tend to gene effector that effector gene are not randomly distributed genome but tend to be with repetitive These have to ‘ speed genome’ which pathogen genome have a biite with gene sparse serving as a cradle for adaptive Here we this how pathogen are to adaptations at time scales We will introduce next phase on this topic

Tomato receptor Ve1 effector pathogen by genome RNA pathogen effector to on their receptor to try to intercept these effector tomato receptor Ve1 governs to fungus Verticillium dahliae Verticillium albo atrum but Verticillium effector By genome a 50 Kb stretch was that only transcriptome Verticillium Nicotiana benthamiana only a ORF this Ave1 for on Ve1 tomato that Ave1 Ve1 that Ave1 markedly to not only on tomato but on Arabidopsis Interestingly Ave1 is to a natriuretic peptide protein were only pathogen Xanthomonas axonopodis fungus Colletotrichum higginsianum Cercospora beticola Fusarium oxysporum lycopersici Ave1 coincident with Ave1 a flexible that Verticillium acquired Ave1 from through gene by we that Ave1 from oxysporum beticola can Ve1 line with this observation Ve1 was to oxysporum tomato showing that this receptor is against pathogen

ToxA Tsn1 for Indian Wheat An Inverse ToxA Tsn1 is an an inverse gene ToxA a toxin HST which as a effector is for pathogen genome pathogen Pyrenophora tritici repentis Parastagonospora nodorum Bipolaris sorokiniana have been to ToxA gene Tsn1 is a sensitivity gene whose helps a ToxA pathogen to wheat Tsn1 are to this is attributed to a other gene which im Tsn1 110 sorokiniana collected from ME5A ME4C megaenvironments India were screened for ToxA gene % were to be ToxA Similarly 220 Indian wheat were screened for Tsn1 gene 81 36 % were to be Tsn1 When wheat 11 with Tsn1 with tsn1 were inoculated with ToxA seedlings only those Tsn1 not tsn1 necrotic spots surrounded by a chlorotic No such distinction between Tsn1 tsn1 carriers was when adult were inoculated This that Tsn1 facilitated against wheat wheat genotypes for Tsn1 can improve to

Programming Phytophthora sojae RXLR effector genome soybean pathogen Phytophthora sojae nearly 400 gene effector protein RXLR EER Here we a survey a sample sojae effector Forty % effector gene sojae for 169 effector most could by BAX effector PAMP INF1 while themselves most effector was prior to was further to A that was weakly prior to but to 120 during h most effector could by effector most effector could INF1 effector branches this misexpression effector sojae transformants

Transcriptome metabolite Zymoseptoria tritici on wheat a biphasic with differential pathogen chromosomal a on definition fungus Zymoseptoria tritici Septoria tritici wheat Triticum aestivum pathogen on wheat that intimate between fungus We deep RNA metabolomics to investigate pathogen an asexual reproductive tritici on wheat Over pathogen gene more than wheat gene more than 300 metabolite were differentially Intriguingly chromosome contributed unequally to gene down gene was inoculated There was for acquisition from symptomless by tritici which instead be utilizing lipid fatty acid stores for fungus then subsequently manipulated carbohydrates fructan metabolite during to This coincided with jasmonic acid gene scale other gene metabolite effector protein were with phase suggested that have overlapping/redundant tritici on wheat through this initial by a slow nutritionally pathogen followed by hyper during a subtle definition

Magnaporthe oryzae effector into Rice Their to Movement about how pathogen that translocate effector protein to neutralize To globally rice hyphae IH invade successive rice Oryza sativa while enclosed extrainvasive hyphal live imaging we a interfacial BIC which accumulates fluorescently labeled effector by IH each newly entered rice effector were into BICs at tips initially hyphae These tip BICs were left behind beside differentiated bulbous IH as fungus continued to Fluorescence recovery after photobleaching that effector protein PWL2 for prevents weeping lovegrass [Eragrostis curvula] continued to accumulate BICs after IH were elsewhere PWL2 BAS1 for protein BIC protein were into rice By BAS4 which uniformly outlines IH was not into Fluorescent PWL2 BAS1 protein that reached rice moved into uninvaded neighbors preparing before We for that underpin effector into BICs to rice to movement rice

assisted to Leptosphaeria maculans Leptosphaeria biglobosa pathogen fungus have a tendency genome by increasing TE Through Leptosphaeria maculans Leptosphaeria biglobosa Dothideomycetes Pleosporales having ranges abilities cruciferous were to infer TE on genome shaping on rise pathogen maculans brassicae most damaging on oilseed rape is only to have a TE invaded genome 32 % to other genome <% These TE had an at by creating TE are suspected to have been instrumental chromosomal rearrangements to TE with gene had an incidence on by promoting translocations effector gene to tuning effector gene maculans brassicae genome by TE followed by bursts TE allowed this to to to its making this genome a peculiarity its own as as Pleosporales

transposon passively actively to speed genome a pathogen to which is especially relevant for pathogen that engage " " with their pathogen gene mediating transposon these transposon therein Here we that transposon are driving force for adaptive genome pathogen Verticillium dahliae We that LS by rearrangements that are by erroneous strand repair utilizing transposon We that duplications are LS against an older episode Finally LS are transposon which to genome we for genome shaping by transposon both an passive which impacts pathogen

Pseudomonas syringae effector RIM Arabidopsis   have that pathogen is a that   pathogen PAMP A uses diseaseresistance R protein to  effector protein that are into     by   pathogen Here we that these are We that Pseudomonas syringae effector AvrRpt2 AvrRpm1 PAMP compromise RIN4 is an Arabidopsis protein by AvrRpt2 AvrRpm1 for We that RIN4 is itself a PAMP R protein RPS2 RPM1 sense  effector RIN4 R protein    against effector that PAMP a link between

effector protein from pathogen Xanthomonas their with Xanthomonas campestris vesicatoria most other pathogen on a TTS which is by hrp gene These gene are are for bacterium to be for TTS protein into milieu effector protein into effector with to pathogen or have an that betrays bacterium to effector were by their with TTS to effector A effector protein are AvrBs3 Xanthomonas AvrBs3 to where it gene that is Xanthomonas is YopJ/AvrRxv latter protein to as SUMO cysteine protease Here we will an overview about TTS its AvrRxv AvrBs3 more detail

protein Pseudomonas syringae is an by pathogen to effector protein into This advancements Pseudomonas syringae gene Hrp pilus effector to date their after into

protein     pathogen protein TTSS is a organelle envelope bacterium it structurally protein into    animal to have basic this needle/pilus formation pores / mRNA requirement chaperone protein for optimal  effector protein most our about TTSS is from bacterium are learned from     bacterium Here we most salient TTSS with special emphasis on

 effector protein agents     bacterium  effector protein into    a Hrp TTSS TTSS these pathogen cannot defeat grow lesions or nonhosts   pathogen  genome projects employing bioinformatic to TTSS Hrp regulon promoters TTSS that Pseudomonas Xanthomonas Ralstonia arsenals effector Hrp TTSS employs customized chaperones envelope by TTSS animal pathogen a more TTSS protein to effector     effector can as agents that betray   pathogen  to    some Investigations effector     have as for effector some effector to cysteine protease or protein tyrosine phosphatase to bacterium

islands Some pathogen have by acquiring islands PIs which are gene PIs  effector  TTS have been gramnegative pathogen TTS are contact can toxin into expanding list bacterium to TTS gene genera Yersinia Salmonella Shigella Escherichia Pseudomonas Bordetella Burkholderia Chlamydia din a     pathogen or symbiont This discusses TTS PIs organisation such for TTS as for treatments

macromolecules by ancestrally to machines are promiscuous macromolecular that human health clinical settings is exceptionally problematic to dissemination gene other has that pathogen mammals Agrobacterium tumefaciens Bordetella pertussis Helicobacter pylori Legionella pneumophila have ancestrally to for purpose delivering effector to Each these DNA or protein to a myriad during ancestrally to are to as list is increasing that macromolecular by these is a phenomenon

Unconventionally effector pathogen salicylate by fungus oomycete pose an increasing threat to ecosystem health These pathogen while taxonomically by secreting effector which are on peptide Here we that Phytophthora sojae Verticillium dahliae isochorismatases PsIsc1 VdIsc1 that are for PsIsc1 VdIsc1 can salicylate hydrolyse isochorismate A triad catalytic is for both protein are isochorismatase effector that disrupt salicylate by its precursor these protein peptide but exhibit characteristics that to unconventional this is a for delivering effector an pathogen

Exploiting Age effector Potato Solanum tuberosum is It suffers from a by Phytophthora infestans This oomycete pathogen RXLR effector that AVR protein which are by R protein from Solanum Most Solanum R gene to have coevolved with infestans at its center Mexico R Avr gene were here we catalog R AVR We that infestans employs for evading R protein ial ial context our effector Genome catalogs infestans effector are enabling effectoromics that R gene R gene with expanded pathogen has Importantly monitoring effector pathogen can assist R gene

    protein R protein bacterium virus or fungus by   pathogen  effector  that are during effector are   pathogen  protein that to for promotion   pathogen  life  effector R protein are their both effector  some R protein as receptor   pathogen  effector protein most to this R protein require protein or Some are by R gene to be technologies arising from proteomics revolution will greatly expand our to investigate R protein

syioses Mycorrhizas are most interkingdom as they involve ~340 land ~50 taxa soil fungus these mutually fungus receive photo carbon with mineral such as phosphorus nitrogen exchange More than 150 on mycorrhizas has raised awareness their biodiversity this we on phylogenomic to fungus their with land As mycorrhizas feature a on ner taxonomy we explore between During fungus have refined their capabilities to take advantage their as sources protective niches while have to accommodate symbiont Intimate associations with pervasive have originated at crossroads between these Our these syioses where fungus as biofertilizers bioprotectors to design biotechnological applications addressing challenges

hierarchical clustering protein to classify rank effector rust fungus Rust fungus are pathogen that considerable on Puccinia graminis tritici wheat stem rust Melampsora larici populina poplar leaf rust pathogen have deleterious impacts on wheat poplar wood pathogen such as rust fungus effector that as modulators can or on effector from other pathogen can be for characterisation effector genome rust fungus We designed a comprehensive silico pipeline to effector from genome rust fungus pipeline is on observation that effector protein from pathogen have at i a ii are by gene have to haustorial protein are cysteine v a effector or a vi are by gene with intergenic vii internal viii do not PFAM except those with We Markov clustering hierarchical clustering to classify protein rust pathogen rank them according to their likelihood being effector this we eight effector that we consider value for This a effector haustorial protein cysteine protein This comprehensive classification effector from these rust pathogen is an initial probing germplasm for

Wheat receptor kinase protein Stb6 controls to pathogen Zymoseptoria tritici gene is most by to fend off pathogen most gene nucleotide leucine protein NLR pathogen Avr effector to This a which halts further Here we wheat Stb6 gene that it a receptor kinase WAK protein which detects a effector pathogen a This by this protein Moreover Stb6 is to our gene specifying to Zymoseptoria tritici an foliar pathogen wheat damaging septoria tritici STB

genome Pseudomonas syringae phaseolicola 1448A gene transposition Pseudomonas syringae phaseolicola a     pathogen is bean this we on genome syringae phaseolicola 1448A which 353 open frames on circular chromosome 928 8 bp plasmids 131 950 by 51 11 bp with a divergent syringae tomato DC3000 a degree conservation at gene genome 133 were as orthologs these a reciprocal best hit method with 941 syntenic these are at they have ranges 1448A beans DC3000 is on tomato Arabidopsis Examination complement survival a substantial but not overlap between these distinguishing feature between is their distinctive With to a fifth pseudomonad genome we were to 56 that Pseudomonas genome 365 that are syringae

genome Puccinia striiformis tritici gene BackgroundThe stripe rust pathogen Puccinia striiformis tritici Pst threats wheat to Pst is by pathogen but are not clearly To Pst previous were from a ethyl methanesulfonate EMS phenotyped for progenitor was asseled annotated for establishing a quality genome were to to for Avr gene ResultsThe reads were mapped to genome to After selecting EMS preferred 264 630 118 913 nucleotide SNP 89 08 2 513 Indels Insertion/deletion were to scaffolds haplotigs Deleterious SNPs Indels occurred 1866 gene Genome 54 gene with A 62 gene were to 16 gene after through for effector degree 48 gene protein SPs gene but with <= 001 to Eight gene were as effector with confidence as they met or to effector ConclusionsGenome with progenitor unraveled a along genome confidence effector gene as for gene Pst gene were from SNPs Indels by artificial these gene are resources for pathogen will be to their between wheat Pst pathogen

Xanthomonas AvrBs3 effector Xanthomonads are pathogen that on to most Xanthomonas is a Hrp T3S that translocates effector protein into effector are to perform a tasks to dissemination We are only beginning to effector effector Xanthomonas is AvrBs3/PthA or TAL TAL effector as activators TAL effector is by a modular DNA Here we TAL effector their

Xanthomonas pathogen this Jones colleagues extremely Xanthomonas how these pathogen their effector for evasion these prototypical pathogen paves to coat Xanthomonas encompass a pathogen that for this we examine into pathogen Xanthomonas Xanthomonas that have substantially our pathogen We emphasize xanthomonads such as effector effector II gene evasion We Xanthomonas implications for outbreaks for

Xanthomonas oryzae pathogen a Xanthomonas oryzae oryzae Xanthomonas oryzae oryzicola leaf streak rice Oryza sativa which constrain this staple Asia Africa Tremendous has been characterizing for oryzae oryzae by while oryzae oryzicola leaf streak by colonizing parenchyma rice there are 29 gene for to but so only a few for leaf streak Over oryzae oryzae have been Both pathogen exhibit have begun to be elucidated AvrBs3/PthA effector a oryzae oryzae oryzae oryzicola rice gene for from maize against leaf streak has a some by gene animal This reviews that spans a It presents a on challenges for opportunities that oryzae with rice as for as as other with implications for animal
