# Supplementary material for: A survey of highly cited studies on plant pathogen effectors during the last two decades (2000-2020)
Source: Front Plant Sci. 2022 Dec 5;13:920281. doi: 10.3389/fpls.2022.920281 (PMC9762492; doi:10.3389/fpls.2022.920281)

Supplementary Figures

**Supplementary Figure S1.** The number of publications pertaining to plant pathogen effectors increased exponentially between 2000 and 2020. The red line and the equation indicate the linear increase of the number of publications related to plant effector biology between 2000 and 2020.


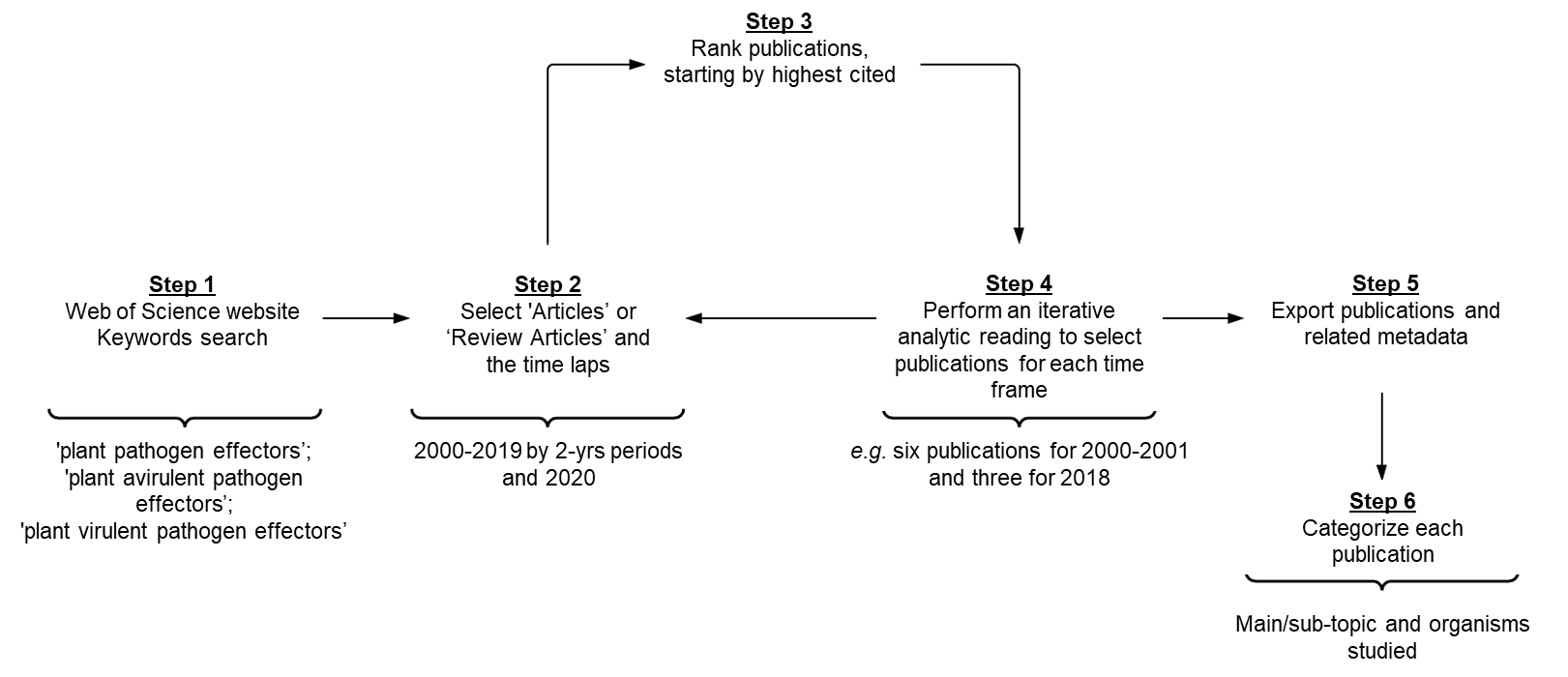


**Supplementary Figure S2.** Operational diagram displaying the step-by-step pipeline used to build the HIPE and HIPE-fun collections. Using the Web of Science portal, we built the HIPE and HIPE-fun collections by using various key words (step 1), article type (step 2), number of citations (step 3) and publication period parameters (step 4; see Supplementary Methods). Secondly (step 5), we collected and archived the pdf version and metadata of each publication into a public Mendeley web folder according to the search output, as follow: 'HIPE collection' (<https://www.zotero.org/groups/4410902/hipe_collection/library>) and 'HIPE-fun collection' (<https://www.zotero.org/groups/4410905/hipe-fun_collection/library>). Finally (step 6), we analyzed both collections to extract from each publication the scientific knowledge brought to readers (*i.e.,* the science) and the structure of the research community behind them (*i.e.,* the people, the labs, the institutions, ...).

**Supplementary Figure S3.** The seven main research topics show comparable citation rates. Boxplots indicating the annual citation rate of the seven main research topics addressed by the HIPEs. The red arrow indicates the HIPE with the highest annual citation rate, which corresponds to the 2006 review by Jones and Dangl that presented the seminal zig-zag model of the plant immune system. The raw data used to build this figure are available in the Dataset 1.


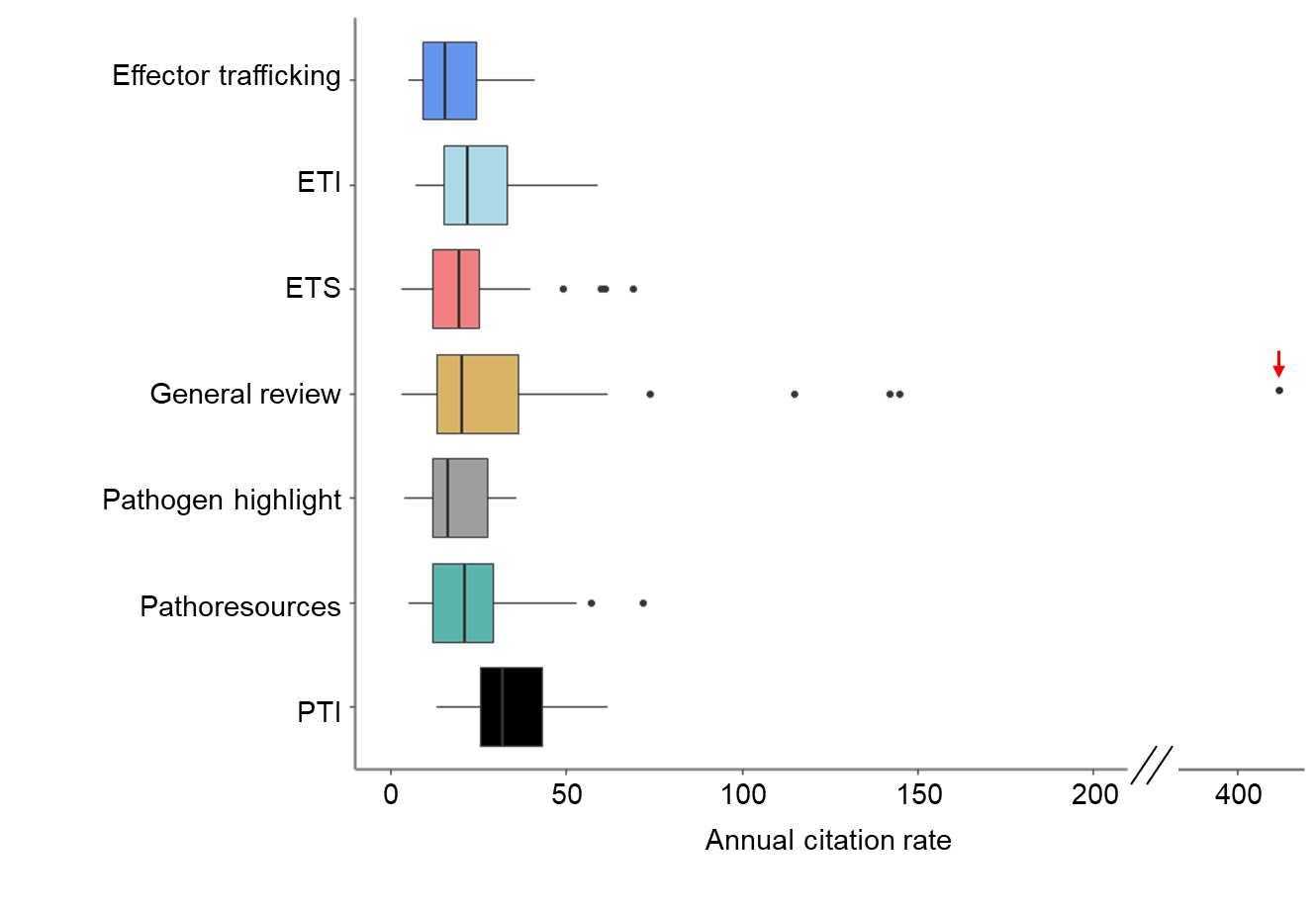

Supplement: Supplementary file 1 [file DataSheet_1.zip › Data Sheet 1 (17)/Supplementary Figures.DOCX]
