# Supplementary material for: A survey of highly cited studies on plant pathogen effectors during the last two decades (2000-2020)
Source: Front Plant Sci. 2022 Dec 5;13:920281. doi: 10.3389/fpls.2022.920281 (PMC9762492; doi:10.3389/fpls.2022.920281)
Supplement: Supplementary file 1 [file DataSheet_1.zip › Data Sheet 1 (17)/Supplementary Methods.docx]

Supplementary Methods

# Building of the HIPE and HIPE-fun collections

To identify and extract publications addressing plant pathogen effectors that have been published between 2000 and 2020 and that have been highly cited, we used a step-by-step pipeline that used the Web of Science website as described previously by Petre et al. (2022), with the following modifications. We used the Mendeley software instead of Zotero. We selected publications following searches with three groups of keywords: 'plant pathogen effector', 'plant pathogen avirulence effector' and 'plant pathogen virulence effector' (see technical note below for details). We defined 12 time periods (2000-2001, 2002-2003, 2004-2005, 2006-2007, 2008-2009, 2010-2011, 2012-2013, 2014-2015, 2016-2017, 2018, 2019, 2020), the last three years have been considered separately due to low citation numbers (grouping them in pairs would have excluded publications from the most recent year). We considered three publications per year (*e.g.* we selected six publications for the time period 2000-2002 and only three publications for the year 2020). In order to import all the metadata of the publications (*e.g.* PDF, title, abstract, author list, journal, ...), we used the 'Mendeley Web Importer' tool. Finally, we removed all redundant publications between the three different extractions to obtain a final collection of 249 non-redundant publications (HIPE collection). In addition, we reperformed the above-mentioned step-by-step pipeline to extract a second corpus of 100 research articles focused only on fungal pathogens (HIPE-fun collection). We archived both HIPE and HIPE-fun collections in a public Mendeley web folder as follow: 'HIPE collection' (<https://www.zotero.org/groups/4410902/hipe_collection/library>) and 'HIPE-fun collection' (<https://www.zotero.org/groups/4410905/hipe-fun_collection/library>).

# Categorization of publications into research topics and organisms studied

To group HIPEs according to their research topics, we performed an iterative analytical reading of the publications and keywords aimed at identifying their main research questions and the organism studied, as described previously (Petre et al*.*, 2022). Throughout this process, we grouped the HIPEs into (i) topics and subtopics according to the main and the specific research question they address, respectively, or (ii) the pathogenic organisms or group of pathogenic organisms they address (Dataset 1). In addition, to evaluate the temporal distribution of the HIPEs within each group cited above, we quantified their number per one six-year time frame (low number of publications during this frame) and four five-year time: 2000-2005, 2006-2010, 2021-2015, and 2016-2020, respectively.

# Generation of word clouds and analysis of key organisms and molecules

To analyze text content, we built text files using Microsoft Excel and Text Wrangler softwares, as described previously (Petre et al*.*, 2022). We first manually copied and pasted the relevant text from Mendeley metadata (publication title and abstract) into a Microsoft Excel spreadsheet (Dataset 1) and we then generated the word clouds containing only words referring to organisms and molecules with filtered text files (Dataset 2). An accurate quantification of the most frequent words was performed using the online WordClouds generator (<https://www.wordclouds.com/>). To generate Excel spreadsheets with the word occurrences, we imported our filtered text files and we used the 'Word list' function to export them.

# Pilot searches selected suitable key words strings to identify the HIPEs

As for our prior study (*i.e.*, *Petre et al*., 2022), before undertaking the present study we performed pilot searches on the Web of Science to optimize the keywords in order to capture the most relevant literature. In a first time, this pilot analysis revealed that the words ‘plant’ and pathogen’ need to be associated with the word ‘effector’ to effectively capture publications that pertain to effectors in the context of plant pathology. Indeed, the word ‘plant’ decisively selects plant science publications (*i.e.*, omitting it from the key word identifies publications from the biomedical field or studies that focus on effectors of animal pathogens). Also, the word ‘pathogen’ decisively selects publications that pertain to plant pathology (*i.e.*, omitting it identifies publications that address signaling molecules, which are termed ‘effectors’ in some fields, in a context of plant growth or of response to abiotic stress for instance). In a second time, we noted that the searches with the key words ‘plant pathogen effector’ identified a significant share of studies in which effectors would not be central (i.e., effectors were used as tools such as cellular probes or immune elicitors, or the word effector just appeared in the abstract of a publication addressing a broader research question in molecular plant pathology). To specify further the searches, we then added word ‘virulence’ or ‘avirulence’ to the key word string ‘plant pathogen effector’; doing so helped capture additional publications that more specifically address either the virulence function of the effectors (*i.e.*, ‘plant pathogen virulence effector’) or the avirulence activity of the effectors (*i.e*., plant pathogen avirulence effector’). The three searches with the key word strings ‘plant pathogen effector’, ‘plant pathogen virulence effector’, and ‘plant pathogen avirulence effector’ yielded diverse sets of publications that we considered relevant based on our own expertise in plant effector biology (only 21 publications out of 249 were redundantly identified by the three searches; see figure 1A in the main manuscript). Therefore, we considered that this combination of keywords was adequate to capture a significant share of the relevant literature in the field (although no combination of keywords can perfectly capture everything relevant in the databases, as discussed in the main manuscript).
