# Supplementary material for: A survey of highly cited studies on plant pathogen effectors during the last two decades (2000-2020)
Source: Front Plant Sci. 2022 Dec 5;13:920281. doi: 10.3389/fpls.2022.920281 (PMC9762492; doi:10.3389/fpls.2022.920281)
Supplement: Supplementary file 1 [file DataSheet_1.zip › Data Sheet 1 (17)/Supplementary Text.DOCX]

Supplementary Text

# The 'Effector trafficking' topic studies how pathogens deliver effectors into host tissues

The 'Effector trafficking' topic comprises 16 publications (six research articles and ten reviews) that collectively gathered around 4.4k citations. Among them, bacterial studies predominate, as only three publications focused on filamentous pathogens. The topic comprises two subtopics: 'Type III/IV bacterial system' and 'Filamentous pathogen translocation pathways'. The 'Type III/IV bacterial system' subtopic comprises 13 publications; the majority of which address the current knowledge on the Type III secretion system (TTSS) and associated effectors (Cornelis and Van Gijsegem, 2000; Büttner and Bonas, 2002a, 2002b; Jin et al., 2003; Alfano and Collmer, 2004; He et al., 2004; Lindeberg et al., 2012; Galán et al., 2014) or the Type IV secretion system (Christie and Vogel, 2000; Christie, 2001). In addition, three publications describe and analyze how hrp/hrc genes encode the Hrp (type III secretion) system and other putative effectors as well as its associated secretion mechanisms (Alfano et al., 2000; Collmer et al., 2000; Lee et al., 2001). The 'Filamentous pathogen translocation pathways' subtopic comprises three publications. One publication addresses the functioning of the biotrophic interfacial complex (BIC) of *Magnaporthe oryzae* (Khang et al., 2010), and two publications focus on how effectors traffic to the host cell cytosol (Whisson et al., 2007; Kale et al., 2010).

# The 'ETI' topic highlights effector recognition-based immunity

The 'ETI' (Effector-Triggered Immunity) topic comprises 41 publications (30 research articles and 11 reviews) that collectively gathered around 9.7k citations. Near 76% of those publications emphasize a specific organism (or group of organisms), including notably fungi and bacteria (41 and 27%, respectively). This topic comprises two subtopics: 'Immune receptor' and 'Effector recognition'. The 'Immune receptor' subtopic comprises 17 publications that summarize and discuss recent findings on the diversity, on the structure, and on the function of NLRs (nucleotide-binding leucine-rich repeat receptors) (van Der Vossen et al., 2000; Martin et al., 2003; Pedley and Martin, 2003; Belkhadir et al., 2004; DeYoung and Innes, 2006; McHale et al., 2006; Shen et al., 2007; Bai et al., 2012; Williams et al., 2014; Cesari et al., 2014; Lu et al., 2016; Saintenac et al., 2018; Marchal et al., 2018; Wang et al., 2019a, 2019b; Jubic et al., 2019) or compare plant and animal immune systems (Maekawa et al., 2011). The 'Effector recognition' subtopic comprises 24 publications. A first set of papers addresses direct and indirect recognition of pathogens by the plant immune system (Ellis et al., 2007; Petit-Houdenot and Fudal, 2017; Kourelis and Van Der Hoorn, 2018). A second set focuses on the direct recognition model following the gene-for-gene model (Allen et al., 2004; Gu et al., 2005; Yang et al., 2006; Dodds et al., 2006; Vleeshouwers et al., 2011; de Jonge et al., 2012; Bourras et al., 2015, 2019; Saur et al., 2019; Navathe et al., 2020; Paulus et al., 2020). A last set studies the indirect recognition of pathogen effectors (Tian et al., 2014) following the guard model (Mackey et al., 2002; Axtell and Staskawicz, 2003; Deslandes et al., 2003; Shao et al., 2003) and finally the recognition mediated by NLR-ID deriving from the decoy model (Cesari et al., 2013; Le Roux et al., 2015; Sarris et al., 2015, 2016; Kroj et al., 2016).

# The 'ETS' topic addresses how pathogens use effectors to promote infection

The 'ETS' (Effector-Triggered Susceptibility) topic comprises 78 publications (45 research articles and 33 reviews) that collectively gathered around 17k citations. Nearly 94% of those publications emphasize bacteria or filamentous pathogens (60 and 40%, respectively). This topic comprises five subtopics: 'Bacteria review', 'Effector expression', 'Filamentous pathogen review', 'Functional characterization' and 'Structural characterization'. The 'Bacteria review' subtopic comprises 17 publications. A first well-defined set of publications summarizes our knowledge on Type III bacterial effectors (Winstanley and Hart, 2001; Greenberg and Vinatzer, 2003; Oh and Beer, 2005; Gürlebeck et al., 2006; Boch and Bonas, 2010; Macho and Zipfel, 2015; Büttner, 2016; Macho, 2016). A second set of publications reviews the recent investigations on intricate molecular interactions with bacterial elicitation and evasion of plant innate immunity (Abramovitch et al., 2006; Deslandes and Rivas, 2012) and comprises comparative analyses with the fungal pathogens (Hogenhout et al., 2009; Khan et al., 2018) or animal-specific bacteria (Orth, 2002; Büttner and Bonas, 2003). One publication studies the impact of auxin on pathogenesis and during the interactions with bacterial pathogens (Kunkel and Harper, 2018). Finally, a last set of publications summarizes researches on the infection pathways of the genus *Xanthomonas sp*. (Büttner and Bonas, 2010) and more precisely the contribution of transcription activator-like effectors (TALEs) (Perez-Quintero and Szurek, 2019). The 'Effector expression' subtopic comprises two publications and highlights the epigenetic regulation of effector gene expression in the fungus *Leptosphaeria maculans* (Soyer et al., 2014) and the regulation of the *hrp* gene cluster encoding the type III protein secretion system (TTSS) (Noël et al., 2001). The 'Filamentous pathogen review' subtopic comprises 12 publications reviewing the recent findings on fungal effectors (Stergiopoulos and de Wit, 2009; Giraldo and Valent, 2013; Stergiopoulos et al., 2013; Tsuge et al., 2013; Anderson et al., 2015; de Sain and Rep, 2015; Lo Presti et al., 2015; Selin et al., 2016; Franceschetti et al., 2017; Han and Kahmann, 2019), on oomycete effectors (Schornack et al., 2009), and on the use of effectors as cellular probes (Toruño et al., 2016). The 'Functional characterization' subtopic comprises 46 publications. Nearly 71% of those publications study the modulation of immune responses by effectors (Mackey et al., 2003; Xia, 2004; Rooney, 2005; Kim et al., 2005; Li et al., 2005; Bos et al., 2006; Nomura, 2006; Zhang et al., 2007, 2010; Xiang et al., 2008; Göhre et al., 2008; Houterman et al., 2008; Gimenez-Ibanez et al., 2009; Feng et al., 2012; Park et al., 2012; Weiberg et al., 2013; Wang et al., 2016; Gao et al., 2019; Hou et al., 2019), compare bacterial and fungal modulation of plant immunity (Göhre and Robatzek, 2008), address phytohormone modulation (Gimenez-Ibanez *et al.*, 2014; Kazan and Lyons, 2014; Liu *et al.*, 2014; Plett *et al.*, 2014), or focus on plant trafficking modulation (Orth, 2000; DebRoy et al., 2004; Brooks et al., 2005; de Torres-Zabala et al., 2007; Kay et al., 2007; Shan et al., 2008; Chen et al., 2010; de Jonge et al., 2010; Mentlak et al., 2012; Aung et al., 2020). A second set of publications identifies avirulence genes and validates their Avr activity (Tsiamis et al., 2000; Zhong et al., 2017) and highlights the masking model as a counter-defense of pathogen to evade the immune system (Plissonneau et al., 2016; Ma et al., 2017). Five publications characterize the activity of type III bacterial effectors (Nimchuk et al., 2000; Abramovitch et al., 2003; Jamir et al., 2004; Abramovitch and Martin, 2005; He et al., 2006) and the associated environmental influences (Xin et al., 2016). Finally, two publications study the virulence activity and virulent motifs of effectors secreted by the soybean pathogen *Phytophthora sojae* (Dou et al., 2008; Ma et al., 2015). The 'Structural characterization' subtopic comprises a single publication that reports the crystal structure of the TALE PthXo1 of *Xanthomonas oryzae* (Mak et al., 2012).

# The 'General review' topic conceptualizes our knowledge on plant-pathogen interactions

The 'General review' topic comprises 39 publications (all reviews) that collectively gathered around 18k citations. Nearly 77% of those publications do not emphasize on a specific or a group of organisms. The topic comprises three subtopics: 'Host immunity', 'Pathosystem evolution' and 'Recognition model'. The 'Host immunity' subtopic comprises 22 publications. A first series of nine publications summarizes and contextualizes recent findings on ETI (Wu et al., 2014; Cui et al., 2015; Thordal-Christensen, 2020), PTI (PAMP-triggered immunity) (Boller and Felix, 2009; Hématy et al., 2009), compares them (Chisholm et al., 2006; Qiu et al., 2008; Dodds and Rathjen, 2010; Tsuda and Katagiri, 2010; Thomma et al., 2011; Liu et al., 2013), or emphasize some taxonomic groups (Liu *et al.*, 2014; Zvereva and Pooggin, 2012). The other set of publications describes the contribution of phytohormones to plant immunity (Zhang and Li, 2019), the regulation and the signaling pathways of host immunity (Feys and Parker, 2000; Hacquard et al., 2017; Wang et al., 2020), the antiviral immunity (Calil and Fontes, 2017; Gouveia et al., 2017), the defense priming (Conrath, 2011), or the non-host resistance in plant (Nürnberger and Lipka, 2005). Finally, two publications compare immunity between plants and animals (Staskawicz et al., 2001) and between bacteria and fungi (van’t Slot and Knogge, 2002). The 'Pathosystem evolution' subtopic comprises eight publications that summarize our knowledge on evolutionary models and mechanisms of plant-pathogen interactions (Brown and Tellier, 2011; Schulze-Lefert and Panstruga, 2011; Raffaele and Kamoun, 2012; Dong et al., 2015; Frantzeskakis et al., 2019) with a focus on climate change (Velásquez et al., 2018) and R-Avr (Resistance gene – Avirulence gene) interactions (Białas et al., 2018). Finally, one publication compares the evolution in bacterial and fungal pathosystems (Dou and Zhou, 2012). The 'Recognition model' subtopic comprises nine publications and proposed different conceptual models focused on the plant immune system (Nimchuk et al., 2001; Bonas and Lahaye, 2002; Jones and Takemoto, 2004; Jones and Dangl, 2006; Bent and Mackey, 2007; van Der Hoorn and Kamoun, 2008; Stotz et al., 2014) and comparative analyses with the animal immune system (Jones et al., 2016). Finally, one publication summarizes specifically bacterial recognitions models (Khan et al., 2016).

# The 'Pathogen highlight' topic emphasizes specific organisms

The 'Pathogen highlight' topic comprises 12 publications (all reviews) that collectively gathered around 1.6k citations. These reviews emphasize bacteria (6), fungi (4), oomycetes (1) and nematodes (1). It comprises three subtopics: 'Bacterium', 'Filamentous pathogen' and 'Nematode'. The 'Bacterium' subtopic comprises six publications and summarize recent findings on the genus *Xanthomonas* (Niño-Liu et al., 2006; An et al., 2020; Kumar et al., 2020; Timilsina et al., 2020) and on the genus *Pseudomonas* (Xin and He, 2013; Xin et al., 2018). The 'Filamentous pathogen' subtopic comprises five publications that summarize recent findings on the oomycete *Phytophthora infestans* (Fry, 2008), on the fungus *Cladosporium fulvum* (de Wit, 2016), on the rust fungi (Lorrain et al., 2019), on the mycorrhizal fungi (Genre et al., 2020) and that draw an overview of selected fungal species with a focus on PAMPs and associated effectors (de Wit et al., 2009). The 'Nematode' subtopic comprises a single publication on the model species *Caenorhabditis elegans* (Alegado et al., 2003).

# The 'Pathoresources' topic shows how omics approaches and technological innovations drive the study of plant-pathogen interactions

The 'Pathoresources' topic comprises 53 publications (42 research articles and 11 reviews) that collectively gathered around 11k citations. It comprises three subtopics: 'Omic data', 'Lab tool' and 'Computational prediction'. The 'Omic data' subtopic comprises 41 publications. Almost half of those publications provide genomic data (Salanoubat et al., 2002; Buell et al., 2003; Genin and Boucher, 2004; Joardar et al., 2005; Thieme et al., 2005; Tyler et al., 2006; Yoshida et al., 2009; Duan et al., 2009; Haas et al., 2009; Spanu et al., 2010; Baltrus et al., 2011; Rouxel et al., 2011; O’Connell et al., 2012; Cooke et al., 2012; Genin and Denny, 2012; Grandaubert et al., 2014; Faino et al., 2016; Kema et al., 2018; Levy et al., 2018; Dale et al., 2019; Laflamme et al., 2020; Li et al., 2020; Wyatt et al., 2020) and four publications provide comparative analyses of genomic data (de Wit et al., 2012; Spanu, 2012; Fouché et al., 2018, 2020). A second set of papers reports effectoromic analyses (Jiang et al., 2008; Vleeshouwers et al., 2008), secretomic analyses (Kamoun, 2006, 2007; Poueymiro and Genin, 2009; Bos et al., 2010; Vincent et al., 2020), transcriptomic analyses (Wang et al., 2011; Rudd et al., 2015), and studies of the effector-target interactome network (Mukhtar et al., 2011; Weßling et al., 2014). Finally, three publications describe the pathogen–host interactions database (PHI-base) providing curated molecular and biological in- formation on gene catalogues associated with pathogen–host interactions (Urban et al., 2015, 2017, 2020). The 'Lab tool' subtopic comprises three publications and describe molecular genetic tools to enhance lab investigations for a better comprehension of plant-pathogen interactions (Nowara et al., 2010; Fang and Tyler, 2016; Ghislain et al., 2019). Finally, the 'Computational prediction' subtopic comprises nine publications which describe pipelines to identify and characterize effectors of filamentous pathogens (Saunders et al., 2012; Sperschneider et al., 2016, 2017, 2018a, 2018b), to identify and characterize effectors of bacteria (Collmer et al., 2002; Chang et al., 2005; Lindeberg et al., 2005), or to annotate NLRs (Steuernagel et al., 2020).

# The 'PTI' topic describes the interplay between immune signaling and effectors

The 'PTI' (Pattern-Triggered Immunity) topic comprises ten publications (four research articles and six reviews) that collectively gathered around 2.7k citations. Among them, five publications do not focus on a specific organism or a group of organisms, four study specifically focus on a bacterium and only one on a fungus. The topic comprises two subtopics: 'Pattern-recognition receptor' and 'Signaling mechanisms'. The 'Pattern-recognition receptor' (PRR) subtopic comprises two reviews that summarize and discuss recent findings on plant PRRs, their diversity to recognize different ligands, and the ability of pathogen effectors to interfere with early PRR signaling (Zipfel, 2009; Tang et al., 2017). The 'Signaling mechanisms' subtopic comprises eight publications. Four publications describe downstream molecular events with: two summarize the complex network of signaling pathways occurring during PTI with an emphasis on mitogen-activated protein kinases (MAPK) and effectors employed to suppress it (Meng and Zhang, 2013; Bigeard et al., 2015); and two highlight the activity of MAPK4 (Qiu et al., 2008) and BIK1 (Lu et al., 2010) in the early signaling pathways and effectors associated with that process. In addition, two publications report the contribution of phytohormones to effector recognition (Tsuda and Katagiri, 2010; Yang et al., 2019), one addresses the role of reactive oxygen species (ROS) in signaling and defense reactions against filamentous fungi and fungal effectors that suppress the host oxidative burst (Heller and Tudzynski, 2011), and one provides evidence of calcium-dependent PTI in plants (Tian et al., 2019).

# References

Abramovitch, R. B., Anderson, J. C., and Martin, G. B. (2006). Bacterial elicitation and evasion of plant innate immunity. *Nat. Rev. Mol. Cell Biol.* 7, 601–611. doi:10.1038/nrm1984.

Abramovitch, R. B., Kim, Y. J., Chen, S., Dickman, M. B., and Martin, G. B. (2003). *Pseudomonas* type III effector AvrPtoB induces plant disease susceptibility by inhibition of host programmed cell death. *EMBO J.* 22, 60–69. doi:10.1093/emboj/cdg006.

Abramovitch, R. B., and Martin, G. B. (2005). AvrPtoB: a bacterial type III effector that both elicits and suppresses programmed cell death associated with plant immunity. *FEMS Microbiol. Lett.* 245, 1–8. doi:10.1016/j.femsle.2005.02.025.

Alegado, R. A., Campbell, M. C., Chen, W. C., Slutz, S. S., and Tan, M. W. (2003). Characterization of mediators of microbial virulence and innate immunity using the *Caenorhabditis elegans* host-pathogen model. *Cell. Microbiol.* 5, 435–444. doi:10.1046/j.1462-5822.2003.00287.x.

Alfano, J. R., Charkowski, A. O., Deng, W. L., Badel, J. L., Petnicki-Ocwieja, T., Van Dijk, K., et al. (2000). The *Pseudomonas syringae* Hrp pathogenicity island has a tripartite mosaic structure composed of a cluster of type III secretion genes bounded by exchangeable effector and conserved effector loci that contribute to parasitic fitness and pathogenicit. *PNAS* 97, 4856–4861. doi:10.1073/pnas.97.9.4856.

Alfano, J. R., and Collmer, A. (2004). Type III secretion system effector proteins: double agents in bacterial disease and plant defense. *Annu. Rev. Phytopathol.* 42, 385–414. doi:10.1146/annurev.phyto.42.040103.110731.

Allen, R. L., Bittner-Eddy, P. D., Grenville-Briggs, L. J., Meitz, J. C., Rehmany, A. P., Rose, L. E., et al. (2004). Host-parasite coevolutionary conflict between *Arabidopsis* and downy mildew. *Science* 306, 1957–1960. doi:10.1126/science.1104022.

An, S. Q., Potnis, N., Dow, M., Vorhölter, F. J., He, Y. Q., Becker, A., et al. (2020). Mechanistic insights into host adaptation, virulence and epidemiology of the phytopathogen *Xanthomonas*. *FEMS Microbiol. Rev.* 44, 1–32. doi:10.1093/femsre/fuz024.

Anderson, R. G., Deb, D., Fedkenheuer, K., and McDowell, J. M. (2015). Recent progress in RXLR effector research. *Mol. Plant-Microbe Interact.* 28, 1063–1072. doi:10.1094/MPMI-01-15-0022-CR.

Aung, K., Kim, P., Li, Z., Joe, A., Kvitko, B., Alfano, J. R., et al. (2020). Pathogenic bacteria target plant plasmodesmata to colonize and invade surrounding tissues. *Plant Cell* 32, 595–611. doi:10.1105/tpc.19.00707.

Axtell, M. J., and Staskawicz, B. J. (2003). Initiation of *RPS2*-specified disease resistance in *Arabidopsis* is coupled to the AvrRpt2-directed elimination of RIN4. *Cell* 112, 369–377. doi:10.1016/S0092-8674(03)00036-9.

Bai, S., Liu, J., Chang, C., Zhang, L., Maekawa, T., Wang, Q., et al. (2012). Structure-function analysis of barley NLR immune receptor MLA10 reveals its cell compartment specific activity in cell death and disease resistance. *PLoS Pathog.* 8, e1002752. doi:10.1371/journal.ppat.1002752.

Baltrus, D. A., Nishimura, M. T., Romanchuk, A., Chang, J. H., Mukhtar, M. S., Cherkis, K., et al. (2011). Dynamic evolution of pathogenicity revealed by sequencing and comparative genomics of 19 *Pseudomonas syringae* isolates. *PLoS Pathog.* 7, e1002132. doi:10.1371/journal.ppat.1002132.

Belkhadir, Y., Subramaniam, R., and Dangl, J. L. (2004). Plant disease resistance protein signaling: NBS-LRR proteins and their partners. *Curr. Opin. Plant Biol.* 7, 391–399. doi:10.1016/j.pbi.2004.05.009.

Bent, A. F., and Mackey, D. (2007). Elicitors, effectors, and *R* genes: the new paradigm and a lifetime supply of questions. *Annu. Rev. Phytopathol.* 45, 399–436. doi:10.1146/annurev.phyto.45.062806.094427.

Białas, A., Zess, E. K., De La Concepcion, J. C., Franceschetti, M., Pennington, H. G., Yoshida, K., et al. (2018). Lessons in effector and NLR biology of plant-microbe systems. *Mol. Plant-Microbe Interact.* 31, 34–45. doi:10.1094/MPMI-08-17-0196-FI.

Bigeard, J., Colcombet, J., and Hirt, H. (2015). Signaling mechanisms in pattern-triggered immunity (PTI). *Mol. Plant* 8, 521–539. doi:10.1016/j.molp.2014.12.022.

Boch, J., and Bonas, U. (2010). *Xanthomonas* AvrBs3 family-type III effectors: discovery and function. *Annu. Rev. Phytopathol.* 48, 419–436. doi:10.1146/annurev-phyto-080508-081936.

Boller, T., and Felix, G. (2009). A renaissance of elicitors: perception of microbe-associated molecular patterns and danger signals by pattern-recognition receptors. *Annu. Rev. Plant Biol.* 60, 379–407. doi:10.1146/annurev.arplant.57.032905.105346.

Bonas, U., and Lahaye, T. (2002). Plant disease resistance triggered by pathogen-derived molecules: Refined models of specific recognition. *Curr. Opin. Microbiol.* 5, 44–50. doi:10.1016/S1369-5274(02)00284-9.

Bos, J. I. B., Kanneganti, T. D., Young, C., Cakir, C., Huitema, E., Win, J., et al. (2006). The C-terminal half of *Phytophthora infestans* RXLR effector AVR3a is sufficient to trigger R3a-mediated hypersensitivity and suppress INF1-induced cell death in *Nicotiana benthamiana*. *Plant J.* 48, 165–176. doi:10.1111/j.1365-313X.2006.02866.x.

Bos, J. I. B., Prince, D., Pitino, M., Maffei, M. E., Win, J., and Hogenhout, S. A. (2010). A functional genomics approach identifies candidate effectors from the aphid species *Myzus persicae* (green peach aphid). *PLoS Genet.* 6, e1001216. doi:10.1371/journal.pgen.1001216.

Bourras, S., Kunz, L., Xue, M., Praz, C. R., Müller, M. C., Kälin, C., et al. (2019). The *AvrPm3-Pm3* effector-NLR interactions control both race-specific resistance and host-specificity of cereal mildews on wheat. *Nat. Commun.* 10, 2292. doi:10.1038/s41467-019-10274-1.

Bourras, S., McNally, K. E., Ben-David, R., Parlange, F., Roffler, S., Praz, C. R., et al. (2015). Multiple avirulence loci and allele-specific effector recognition control the *Pm3* race-specific resistance of wheat to powdery mildew. *Plant Cell* 27, 2991–3012. doi:10.1105/tpc.15.00171.

Brooks, D. M., Bender, C. L., and Kunkel, B. N. (2005). The *Pseudomonas syringae* phytotoxin coronatine promotes virulence by overcoming salicylic acid-dependent defences in *Arabidopsis thaliana*. *Mol. Plant Pathol.* 6, 629–639. doi:10.1111/j.1364-3703.2005.00311.x.

Brown, J. K. M., and Tellier, A. (2011). Plant-parasite coevolution: bridging the gap between genetics and ecology. *Annu. Rev. Phytopathol.* 49, 345–367. doi:10.1146/annurev-phyto-072910-095301.

Buell, C. R., Joardar, V., Lindeberg, M., Selengut, J., Paulsen, I. T., Gwinn, M. L., et al. (2003). The complete genome sequence of the *Arabidopsis* and tomato pathogen *Pseudomonas syringae* pv. *tomato* DC3000. *PNAS* 100, 10181–10186. doi:10.1073/pnas.1731982100.

Büttner, D. (2016). Behind the lines-actions of bacterial type III effector proteins in plant cells. *FEMS Microbiol. Rev.* 40, 894–937. doi:10.1093/femsre/fuw026.

Büttner, D., and Bonas, U. (2002a). Getting across - bacterial type III effector proteins on their way to the plant cell. *EMBO J.* 21, 5313–5322. doi:10.1093/emboj/cdf536.

Büttner, D., and Bonas, U. (2002b). Port of entry - the type III secretion translocon. *Trends Microbiol.* 10, 186–192. doi:10.1016/S0966-842X(02)02331-4.

Büttner, D., and Bonas, U. (2003). Common infection strategies of plant and animal pathogenic bacteria. *Curr. Opin. Plant Biol.* 6, 312–319. doi:10.1016/S1369-5266(03)00064-5.

Büttner, D., and Bonas, U. (2010). Regulation and secretion of *Xanthomonas* virulence factors. *FEMS Microbiol. Rev.* 34, 107–133. doi:10.1111/j.1574-6976.2009.00192.x.

Calil, I. P., and Fontes, E. P. B. (2017). Plant immunity against viruses: antiviral immune receptors in focus. *Ann. Bot.* 119, 711–723. doi:10.1093/aob/mcw200.

Cesari, S., Kanzaki, H., Fujiwara, T., Bernoux, M., Chalvon, V., Kawano, Y., et al. (2014). The NB‐LRR proteins RGA4 and RGA5 interact functionally and physically to confer disease resistance. *EMBO J.* 33, 1941–1959. doi:10.15252/embj.201487923.

Cesari, S., Thilliez, G., Ribot, C., Chalvon, V., Michel, C., Jauneau, A., et al. (2013). The rice resistance protein pair RGA4/RGA5 recognizes the *Magnaporthe oryzae* effectors AVR-Pia and AVR1-CO39 by direct binding. *Plant Cell* 25, 1463–1481. doi:10.1105/tpc.112.107201.

Chang, J. H., Urbach, J. M., Law, T. F., Arnold, L. W., Hu, A., Gombar, S., et al. (2005). A high-throughput, near-saturating screen for type III effector genes from *Pseudomonas syringae*. *PNAS* 102, 2549–2554. doi:10.1073/pnas.0409660102.

Chen, L. Q., Hou, B. H., Lalonde, S., Takanaga, H., Hartung, M. L., Qu, X. Q., et al. (2010). Sugar transporters for intercellular exchange and nutrition of pathogens. *Nature* 468, 527–532. doi:10.1038/nature09606.

Chisholm, S. T., Coaker, G., Day, B., and Staskawicz, B. J. (2006). Host-microbe interactions: shaping the evolution of the plant immune response. *Cell* 124, 803–814. doi:10.1016/j.cell.2006.02.008.

Christie, P. J. (2001). Type IV secretion: intercellular transfer of macromolecules by systems ancestrally related to conjugation machines. *Mol. Microbiol.* 40, 294–305. doi:10.1046/j.1365-2958.2001.02302.x.

Christie, P. J., and Vogel, J. P. (2000). Bacterial type IV secretion: conjugation systems adapted to deliver effector molecules to host cells. *Trends Microbiol.* 8, 354–360. doi:10.1016/S0966-842X(00)01792-3.

Collmer, A., Badel, J. L., Charkowski, A. O., Deng, W.-L., Fouts, D. E., Ramos, A. R., et al. (2000). *Pseudomonas syringae* Hrp type III secretion system and effector proteins. *PNAS* 97, 8770–8777. doi:10.1073/pnas.97.16.8770.

Collmer, A., Lindeberg, M., Petnicki-Ocwieja, T., Schneider, D. J., and Alfano, J. R. (2002). Genomic mining type III secretion system effectors in *Pseudomonas syringae* yields new picks for all TTSS prospectors. *Trends Microbiol.* 10, 462–469. doi:10.1016/S0966-842X(02)02451-4.

Conrath, U. (2011). Molecular aspects of defence priming. *Trends Plant Sci.* 16, 524–531. doi:10.1016/j.tplants.2011.06.004.

Cooke, D. E. L., Cano, L. M., Raffaele, S., Bain, R. A., Cooke, L. R., Etherington, G. J., et al. (2012). Genome analyses of an aggressive and invasive lineage of the irish potato famine pathogen. *PLoS Pathog.* 8, e1002940. doi:10.1371/journal.ppat.1002940.

Cornelis, G. R., and Van Gijsegem, F. (2000). Assembly and function of type III secretory systems. *Annu. Rev. Microbiol.* 54, 735–774. doi:10.1146/annurev.micro.54.1.735.

Cui, H., Tsuda, K., and Parker, J. E. (2015). Effector-triggered immunity: from pathogen perception to robust defense. *Annu. Rev. Plant Biol.* 66, 487–511. doi:10.1146/annurev-arplant-050213-040012.

Dale, A. L., Feau, N., Everhart, S. E., Dhillon, B., Wong, B., Sheppard, J., et al. (2019). Mitotic recombination and rapid genome evolution in the invasive forest pathogen *Phytophthora ramorum*. *MBio* 10, e02452-18. doi:10.1128/mBio.02452-18.

de Jonge, R., Van Esse, H. P., Kombrink, A., Shinya, T., Desaki, Y., Bours, R., et al. (2010). Conserved fungal LysM effector Ecp6 prevents chitin-triggered immunity in plants. *Science* 329, 953–955. doi:10.1126/science.1190859.

de Jonge, R., Van Esse, H. P., Maruthachalam, K., Bolton, M. D., Santhanam, P., Saber, M. K., et al. (2012). Tomato immune receptor Ve1 recognizes effector of multiple fungal pathogens uncovered by genome and RNA sequencing. *PNAS* 109, 5110–5115. doi:10.1073/pnas.1119623109.

de Sain, M., and Rep, M. (2015). The role of pathogen-secreted proteins in fungal vascular wilt diseases. *Int. J. Mol. Sci.* 16, 23970–23993. doi:10.3390/ijms161023970.

de Torres-Zabala, M., Truman, W., Bennett, M. H., Lafforgue, G., Mansfield, J. W., Rodriguez Egea, P., et al. (2007). *Pseudomonas syringae* pv. *tomato* hijacks the *Arabidopsis* abscisic acid signalling pathway to cause disease. *EMBO J.* 26, 1434–1443. doi:10.1038/sj.emboj.7601575.

de Wit, P. J. G. M. (2016). *Cladosporium fulvum* effectors: weapons in the arms race with tomato. *Annu. Rev. Phytopathol.* 54, 1–23. doi:10.1146/annurev-phyto-011516-040249.

de Wit, P. J. G. M., Mehrabi, R., Van Den Burg, H. A., and Stergiopoulos, I. (2009). Fungal effector proteins: past, present and future. *Mol. Plant Pathol.* 10, 735–747. doi:10.1111/j.1364-3703.2009.00591.x.

de Wit, P. J. G. M., Van Der Burgt, A., Ökmen, B., Stergiopoulos, I., Abd-Elsalam, K. A., Aerts, A. L., et al. (2012). The genomes of the fungal plant pathogens *Cladosporium fulvum* and *Dothistroma septosporum* reveal adaptation to different hosts and lifestyles but also signatures of common ancestry. *PLoS Genet.* 8, e1003088. doi:10.1371/journal.pgen.1003088.

DebRoy, S., Thilmony, R., Kwack, Y.-B., Nomura, K., and He, S. Y. (2004). A family of conserved bacterial effectors inhibits salicylic acid-mediated basal immunity and promotes disease necrosis in plants. *PNAS* 101, 9927–9932. doi:10.1073/pnas.0401601101.

Deslandes, L., Olivier, J., Peeters, N., Feng, D. X., Khounlotham, M., Boucher, C., et al. (2003). Physical interaction between RRS1-R, a protein conferring resistance to bacterial wilt, and PopP2, a type III effector targeted to the plant nucleus. *PNAS* 100, 8024–8029. doi:10.1073/pnas.1230660100.

Deslandes, L., and Rivas, S. (2012). Catch me if you can: bacterial effectors and plant targets. *Trends Plant Sci.* 17, 644–655. doi:10.1016/j.tplants.2012.06.011.

DeYoung, B. J., and Innes, R. W. (2006). Plant NBS-LRR proteins in pathogen sensing and host defense. *Nat. Immunol.* 7, 1243–1249. doi:10.1038/ni1410.

Dodds, P. N., Lawrence, G. J., Catanzariti, A.-M., Teh, T., Wang, C.-I. A., Ayliffe, M. A., et al. (2006). Direct protein interaction underlies gene-for-gene specificity and coevolution of the flax resistance genes and flax rust avirulence genes. *PNAS* 103, 8888–8893. doi: 10.1073/pnas.0602577103.

Dodds, P. N., and Rathjen, J. P. (2010). Plant immunity: towards an integrated view of plant-pathogen interactions. *Nat. Rev. Genet.* 11, 539–548. doi:10.1038/nrg2812.

Dong, S., Raffaele, S., and Kamoun, S. (2015). The two-speed genomes of filamentous pathogens: Waltz with plants. *Curr. Opin. Genet. Dev.* 35, 57–65. doi:10.1016/j.gde.2015.09.001.

Dou, D., Kale, S. D., Wang, X., Chen, Y., Wang, Q., Wang, X., et al. (2008). Conserved C-terminal motifs required for avirulence and suppression of cell death by *Phytophthora sojae* effector Avr1b. *Plant Cell* 20, 1118–1133. doi:10.1105/tpc.107.057067.

Dou, D., and Zhou, J. M. (2012). Phytopathogen effectors subverting host immunity: different foes, similar battleground. *Cell Host Microbe* 12, 484–495. doi:10.1016/j.chom.2012.09.003.

Duan, Y., Zhou, L., Hall, D. G., Li, W., Doddapaneni, H., Lin, H., et al. (2009). Complete genome sequence of citrus huanglongbing bacterium, “*Candidatus liberibacter asiaticus*” obtained through metagenomics. *Mol. Plant-Microbe Interact.* 22, 1011–1020. doi:10.1094/MPMI-22-8-1011.

Ellis, J. G., Dodds, P. N., and Lawrence, G. J. (2007). Flax rust resistance gene specificity is based on direct resistance-avirulence protein interactions. *Annu. Rev. Phytopathol.* 45, 289–306. doi:10.1146/annurev.phyto.45.062806.094331.

Faino, L., Seidl, M. F., Shi-Kunne, X., Pauper, M., Van Den Berg, G. C. M., Wittenberg, A. H. J., et al. (2016). Transposons passively and actively contribute to evolution of the two-speed genome of a fungal pathogen. *Genome Res.* 26, 1091–1100. doi:10.1101/gr.204974.116.

Fang, Y., and Tyler, B. M. (2016). Efficient disruption and replacement of an effector gene in the oomycete *Phytophthora sojae* using CRISPR/Cas9. *Mol. Plant Pathol.* 17, 127–139. doi:10.1111/mpp.12318.

Feng, F., Yang, F., Rong, W., Wu, X., Zhang, J., Chen, S., et al. (2012). A *Xanthomonas* uridine 5′-monophosphate transferase inhibits plant immune kinases. *Nature* 485, 114–118. doi:10.1038/nature10962.

Feys, B. J., and Parker, J. E. (2000). Interplay of signaling pathways in plant disease resistance. *Trends Genet.* 16, 449–455. doi:10.1016/S0168-9525(00)02107-7.

Fouché, S., Badet, T., Oggenfuss, U., Plissonneau, C., Francisco, C. S., and Croll, D. (2020). Stress-driven transposable element de-repression dynamics and virulence evolution in a fungal pathogen. *Mol. Biol. Evol.* 37, 221–239. doi:10.1093/molbev/msz216.

Fouché, S., Plissonneau, C., and Croll, D. (2018). The birth and death of effectors in rapidly evolving filamentous pathogen genomes. *Curr. Opin. Microbiol.* 46, 34–42. doi:10.1016/j.mib.2018.01.020.

Franceschetti, M., Maqbool, A., Jiménez-Dalmaroni, M. J., Pennington, H. G., Kamoun, S., and Banfield, M. J. (2017). Effectors of filamentous plant pathogens: commonalities amid diversity. *Microbiol. Mol. Biol. Rev.* 81, e00066-16. doi:10.1128/mmbr.00066-16.

Frantzeskakis, L., Di Pietro, A., Rep, M., Schirawski, J., Wu, C. H., and Panstruga, R. (2019). Rapid evolution in plant–microbe interactions – a molecular genomics perspective. *New Phytol.* 225, 1134–1142. doi:10.1111/nph.15966.

Fry, W. (2008). *Phytophthora infestans*: the plant (and *R* gene) destroyer. *Mol. Plant Pathol.* 9, 385–402. doi:10.1111/j.1364-3703.2007.00465.x.

Galán, J. E., Lara-Tejero, M., Marlovits, T. C., and Wagner, S. (2014). Bacterial type III secretion systems: Specialized nanomachines for protein delivery into target cells. *Annu. Rev. Microbiol.* 68, 415–438. doi:10.1146/annurev-micro-092412-155725.

Gao, F., Zhang, B. Sen, Zhao, J. H., Huang, J. F., Jia, P. S., Wang, S., et al. (2019). Deacetylation of chitin oligomers increases virulence in soil-borne fungal pathogens. *Nat. Plants* 5, 1167–1176. doi:10.1038/s41477-019-0527-4.

Genin, S., and Boucher, C. (2004). Lessons learned from the genome analysis of *Ralstonia solanacearum*. *Annu. Rev. Phytopathol.* 42, 107–134. doi:10.1146/annurev.phyto.42.011204.104301.

Genin, S., and Denny, T. P. (2012). Pathogenomics of the *Ralstonia solanacearum* species complex. *Annu. Rev. Phytopathol.* 50, 67–89. doi:10.1146/annurev-phyto-081211-173000.

Genre, A., Lanfranco, L., Perotto, S., and Bonfante, P. (2020). Unique and common traits in mycorrhizal symbioses. *Nat. Rev. Microbiol.* 18, 649–660. doi:10.1038/s41579-020-0402-3.

Ghislain, M., Byarugaba, A. A., Magembe, E., Njoroge, A., Rivera, C., Román, M. L., et al. (2019). Stacking three late blight resistance genes from wild species directly into African highland potato varieties confers complete field resistance to local blight races. *Plant Biotechnol. J.* 17, 1119–1129. doi:10.1111/pbi.13042.

Gimenez-Ibanez, S., Boter, M., Fernández-Barbero, G., Chini, A., Rathjen, J. P., and Solano, R. (2014). The bacterial effector HopX1 targets JAZ transcriptional repressors to activate jasmonate signaling and promote infection in Arabidopsis. *PLoS Biol.* 12, e1001792. doi:10.1371/journal.pbio.1001792.

Gimenez-Ibanez, S., Hann, D. R., Ntoukakis, V., Petutschnig, E., Lipka, V., and Rathjen, J. P. (2009). AvrPtoB targets the LysM receptor kinase CERK1 to promote bacterial virulence on plants. *Curr. Biol.* 19, 423–429. doi:10.1016/j.cub.2009.01.054.

Giraldo, M. C., and Valent, B. (2013). Filamentous plant pathogen effectors in action. *Nat. Rev. Microbiol.* 11, 800–814. doi:10.1038/nrmicro3119.

Göhre, V., and Robatzek, S. (2008). Breaking the barriers: microbial effector molecules subvert plant immunity. *Annu. Rev. Phytopathol.* 46, 189–215. doi:10.1146/annurev.phyto.46.120407.110050.

Göhre, V., Spallek, T., Häweker, H., Mersmann, S., Mentzel, T., Boller, T., et al. (2008). Plant pattern-recognition receptor FLS2 is directed for degradation by the bacterial ubiquitin ligase AvrPtoB. *Curr. Biol.* 18, 1824–1832. doi:10.1016/j.cub.2008.10.063.

Gouveia, B. C., Calil, I. P., Machado, J. P. B., Santos, A. A., and Fontes, E. P. B. (2017). Immune receptors and co-receptors in antiviral innate immunity in plants. *Front. Microbiol.* 7, 2139. doi:10.3389/fmicb.2016.02139.

Grandaubert, J., Lowe, R. G. T., Soyer, J. L., Schoch, C. L., Van De Wouw, A. P., Fudal, I., et al. (2014). Transposable element-assisted evolution and adaptation to host plant within the *Leptosphaeria maculans-Leptosphaeria biglobosa* species complex of fungal pathogens. *BMC Genomics* 15, 891. doi:10.1186/1471-2164-15-891.

Greenberg, J. T., and Vinatzer, B. A. (2003). Identifying type III effectors of plant pathogens and analyzing their interaction with plant cells. *Curr. Opin. Microbiol.* 6, 20–28. doi:10.1016/S1369-5274(02)00004-8.

Gu, K., Yang, B., Tian, D., Wu, L., Wang, D., Sreekala, C., et al. (2005). *R* gene expression induced by a type-III effector triggers disease resistance in rice. *Nature* 435, 1122–1125. doi:10.1038/nature03630.

Gürlebeck, D., Thieme, F., and Bonas, U. (2006). Type III effector proteins from the plant pathogen <i>Xanthomonas</i< and their role in the interaction with the host plant. *J. Plant Physiol.* 163, 233–255. doi:10.1016/j.jplph.2005.11.011.

Haas, B. J., Kamoun, S., Zody, M. C., Jiang, R. H. Y., Handsaker, R. E., Cano, L. M., et al. (2009). Genome sequence and analysis of the Irish potato famine pathogen *Phytophthora infestans*. *Nature* 461, 393–398. doi:10.1038/nature08358.

Hacquard, S., Spaepen, S., Garrido-Oter, R., and Schulze-Lefert, P. (2017). Interplay between innate immunity and the plant microbiota. *Annu. Rev. Phytopathol.* 55, 565–589. doi:10.1146/annurev-phyto-080516-035623.

Han, X., and Kahmann, R. (2019). Manipulation of phytohormone pathways by effectors of filamentous plant pathogens. *Front. Plant Sci.* 10, 822. doi:10.3389/fpls.2019.00822.

He, P., Shan, L., Lin, N. C., Martin, G. B., Kemmerling, B., Nürnberger, T., et al. (2006). Specific bacterial suppressors of MAMP signaling upstream of MAPKKK in *Arabidopsis* innate immunity. *Cell* 125, 563–575. doi:10.1016/j.cell.2006.02.047.

He, S. Y., Nomura, K., and Whittam, T. S. (2004). Type III protein secretion mechanism in mammalian and plant pathogens. *BBA* 1694, 181–206. doi:10.1016/j.bbamcr.2004.03.011.

Heller, J., and Tudzynski, P. (2011). Reactive oxygen species in phytopathogenic fungi: signaling, development, and disease. *Annu. Rev. Phytopathol.* 49, 369–390. doi:10.1146/annurev-phyto-072910-095355.

Hématy, K., Cherk, C., and Somerville, S. (2009). Host-pathogen warfare at the plant cell wall. *Curr. Opin. Plant Biol.* 12, 406–413. doi:10.1016/j.pbi.2009.06.007.

Hogenhout, S. A., Van Der Hoorn, R. A. L., Terauchi, R., and Kamoun, S. (2009). Emerging concepts in effector biology of plant-associated organisms. *Mol. Plant-Microbe Interact.* 22, 115–122. doi:10.1094/MPMI.

Hou, Y., Zhai, Y., Feng, L., Karimi, H. Z., Rutter, B. D., Zeng, L., et al. (2019). A *Phytophthora* effector suppresses trans-kingdom RNAi to promote disease susceptibility. *Cell Host Microbe* 25, 153-165.e5. doi:10.1016/j.chom.2018.11.007.

Houterman, P. M., Cornelissen, B. J. C., and Rep, M. (2008). Suppression of plant resistance gene-based immunity by a fungal effector. *PLoS Pathog.* 4. doi:10.1371/journal.ppat.1000061.

Jamir, Y., Guo, M., Oh, H. S., Petnicki-Ocwieja, T., Chen, S., Tang, X., et al. (2004). Identification of *Pseudomonas syringae* type III effectors that can suppress programmed cell death in plants and yeast. *Plant J.* 37, 554–565. doi:10.1046/j.1365-313X.2003.01982.x.

Jiang, R. H. Y., Tripathy, S., Govers, F., and Tyler, B. M. (2008). RXLR effector reservoir in two *Phytophthora* species is dominated by a single rapidly evolving superfamily with more than 700 members. *PNAS* 105, 4874–4879. doi:10.1073/pnas.0709303105.

Jin, Q., Thilmony, R., Zwiesler-Vollick, J., and He, S. Y. (2003). Type III protein secretion in *Pseudomonas syringae*. *Microbes Infect.* 5, 301–310. doi:10.1016/S1286-4579(03)00032-7.

Joardar, V., Lindeberg, M., Jackson, R. W., Selengut, J., Dodson, R., Brinkac, L. M., et al. (2005). Whole-genome sequence analysis of *Pseudomonas syringae* pv. *phaseolicola* 1448A reveals divergence among pathovars in genes involved in virulence and transposition. *J. Bacteriol.* 187, 6488–6498. doi:10.1128/JB.187.18.6488-6498.2005.

Jones, D. A., and Takemoto, D. (2004). Plant innate immunity - direct and indirect recognition of general and specific pathogen-associated molecules. *Curr. Opin. Immunol.* 16, 48–62. doi:10.1016/j.coi.2003.11.016.

Jones, J. D. G., and Dangl, J. L. (2006). The plant immune system. *Nature* 444, 323–329. doi:10.1038/nature05286.

Jones, J. D. G., Vance, R. E., and Dangl, J. L. (2016). Intracellular innate immune surveillance devices in plants and animals. *Science*  354, aaf6395. doi:10.1126/science.aaf6395.

Jubic, L. M., Saile, S., Furzer, O. J., El Kasmi, F., and Dangl, J. L. (2019). Help wanted: helper NLRs and plant immune responses. *Curr. Opin. Plant Biol.* 50, 82–94. doi:10.1016/j.pbi.2019.03.013.

Kale, S. D., Gu, B., Capelluto, D. G. S., Dou, D., Feldman, E., Rumore, A., et al. (2010). External lipid PI3P mediates entry of eukaryotic pathogen effectors into plant and animal host cells. *Cell* 142, 284–295. doi:10.1016/j.cell.2010.06.008.

Kamoun, S. (2006). A catalogue of the effector secretome of plant pathogenic oomycetes. *Annu. Rev. Phytopathol.* 44, 41–60. doi:10.1146/annurev.phyto.44.070505.143436.

Kamoun, S. (2007). Groovy times: filamentous pathogen effectors revealed. *Curr. Opin. Plant Biol.* 10, 358–365. doi:10.1016/j.pbi.2007.04.017.

Kay, S., Hahn, S., Marois, E., Hause, G., and Bonas, U. (2007). A bacterial effector acts as a plant transcription factor and induces a cell size regulator. *Science* 318, 648–651. doi:10.1126/science.1144956.

Kazan, K., and Lyons, R. (2014). Intervention of phytohormone pathways by pathogen effectors. *Plant Cell* 26, 2285–2309. doi:10.1105/tpc.114.125419.

Kema, G. H. J., Mirzadi Gohari, A., Aouini, L., Gibriel, H. A. Y., Ware, S. B., van den Bosch, F., et al. (2018). Stress and sexual reproduction affect the dynamics of the wheat pathogen effector AvrStb6 and strobilurin resistance. *Nat. Genet.* 50, 375–380. doi:10.1038/s41588-018-0052-9.

Khan, M., Seto, D., Subramaniam, R., and Desveaux, D. (2018). Oh, the places they’ll go! A survey of phytopathogen effectors and their host targets. *Plant J.* 93, 651–663. doi:10.1111/tpj.13780.

Khan, M., Subramaniam, R., and Desveaux, D. (2016). Of guards, decoys, baits and traps: pathogen perception in plants by type III effector sensors. *Curr. Opin. Microbiol.* 29, 49–55. doi:10.1016/j.mib.2015.10.006.

Khang, C. H., Berruyer, R., Giraldo, M. C., Kankanala, P., Park, S. Y., Czymmek, K., et al. (2010). Translocation of *Magnaporthe oryzae* effectors into rice cells and their subsequent cell-to-cell movement. *Plant Cell* 22, 1388–1403. doi:10.1105/tpc.109.069666.

Kim, M. G., Da Cunha, L., McFall, A. J., Belkhadir, Y., DebRoy, S., Dangl, J. L., et al. (2005). Two *Pseudomonas syringae* type III effectors inhibit RIN4-regulated basal defense in *Arabidopsis*. *Cell* 121, 749–759. doi:10.1016/j.cell.2005.03.025.

Kourelis, J., and Van Der Hoorn, R. A. L. (2018). Defended to the nines: 25 years of resistance gene cloning identifies nine mechanisms for R protein function. *Plant Cell* 30, 285–299. doi:10.1105/tpc.17.00579.

Kroj, T., Chanclud, E., Michel-Romiti, C., Grand, X., and Morel, J. B. (2016). Integration of decoy domains derived from protein targets of pathogen effectors into plant immune receptors is widespread. *New Phytol.* 210, 618–626. doi:10.1111/nph.13869.

Kumar, A., Kumar, R., Sengupta, D., Das, S. N., Pandey, M. K., Bohra, A., et al. (2020). Deployment of genetic and genomic tools toward gaining a better understanding of rice-Xanthomonasoryzae pv. oryzae interactions for development of durable bacterial blight resistant rice. *Front. Plant Sci.* 11, 1152. doi:10.3389/fpls.2020.01152.

Kunkel, B. N., and Harper, C. P. (2018). The roles of auxin during interactions between bacterial plant pathogens and their hosts. *J. Exp. Bot.* 69, 245–254. doi:10.1093/jxb/erx447.

Laflamme, B., Dillon, M. M., Martel, A., Almeida, R. N. D., Desveaux, D., and Guttman, D. S. (2020). The pan-genome effector-triggered immunity landscape of a host-pathogen interaction. *Science (80-. ).* 367, 763–768. doi:10.1126/science.aax4079.

Le Roux, C., Huet, G., Jauneau, A., Camborde, L., Trémousaygue, D., Kraut, A., et al. (2015). A receptor pair with an integrated decoy converts pathogen disabling of transcription factors to immunity. *Cell* 161, 1074–1088. doi:10.1016/j.cell.2015.04.025.

Lee, J., Klüsener, B., Tsiamis, G., Stevens, C., Neyt, C., Tampakaki, A. P., et al. (2001). HrpZPsph from the plant pathogen *Pseudomonas syringae* pv. *phaseolicola* binds to lipid bilayers and forms an ion-conducting pore *in vitro*. *PNAS* 98, 289–294. doi:10.1073/pnas.011265298.

Levy, A., Salas Gonzalez, I., Mittelviefhaus, M., Clingenpeel, S., Herrera Paredes, S., Miao, J., et al. (2018). Genomic features of bacterial adaptation to plants. *Nat. Genet.* 50, 138–150. doi:10.1038/s41588-017-0012-9.

Li, X., Lin, H., Zhang, W., Zou, Y., Zhang, J., Tang, X., et al. (2005). Flagellin induces innate immunity in nonhost interactions that is suppressed by *Pseudomonas syringae* effectors. *PNAS* 102, 12990–12995. doi:10.1073/pnas.0502425102.

Li, Y., Xia, C., Wang, M., Yin, C., and Chen, X. (2020). Whole-genome sequencing of *Puccinia striiformis* f. sp. *tritici* mutant isolates identifies avirulence gene candidates. *BMC Genomics* 21, 247. doi:10.1186/s12864-020-6677-y.

Lindeberg, M., Cunnac, S., and Collmer, A. (2012). *Pseudomonas syringae* type III effector repertoires: last words in endless arguments. *Trends Microbiol.* 20, 199–208. doi:10.1016/j.tim.2012.01.003.

Lindeberg, M., Stavrinides, J., Chang, J. H., Alfano, J. R., Collmer, A., Dangl, J. L., et al. (2005). Proposed guidelines for a unified nomenclature and phylogenetic analysis of type III hop effector proteins in the plant pathogen *Pseudomonas syringae*. *Mol. Plant-Microbe Interact.* 18, 275–282. doi:10.1094/MPMI-18-0275.

Liu, T., Song, T., Zhang, X., Yuan, H., Su, L., Li, W., et al. (2014a). Unconventionally secreted effectors of two filamentous pathogens target plant salicylate biosynthesis. *Nat. Commun.* 5, 4686. doi:10.1038/ncomms5686.

Liu, W., Liu, J., Ning, Y., Ding, B., Wang, X., Wang, Z., et al. (2013). Recent progress in understanding PAMP-and effector-triggered immunity against the rice blast fungus *Magnaporthe oryzae*. *Mol. Plant* 6, 605–620. doi:10.1093/mp/sst015.

Liu, W., Liu, J., Triplett, L., Leach, J. E., and Wang, G. L. (2014b). Novel insights into rice innate immunity against bacterial and fungal pathogens. *Annu. Rev. Phytopathol.* 52, 213–241. doi:10.1146/annurev-phyto-102313-045926.

Lo Presti, L., Lanver, D., Schweizer, G., Tanaka, S., Liang, L., Tollot, M., et al. (2015). Fungal effectors and plant susceptibility. *Annu. Rev. Plant Biol.* 66, 513–545. doi:10.1146/annurev-arplant-043014-114623.

Lorrain, C., Gonçalves dos Santos, K. C., Germain, H., Hecker, A., and Duplessis, S. (2019). Advances in understanding obligate biotrophy in rust fungi. *New Phytol.* 222, 1190–1206. doi:10.1111/nph.15641.

Lu, D., Wu, S., Gao, X., Zhang, Y., Shan, L., and He, P. (2010). A receptor-like cytoplasmic kinase, BIK1, associates with a flagellin receptor complex to initiate plant innate immunity. *PNAS* 107, 496–501. doi:10.1073/pnas.0909705107.

Lu, X., Kracher, B., Saur, I. M. L., Bauer, S., Ellwood, S. R., Wise, R., et al. (2016). Allelic barley MLA immune receptors recognize sequence-unrelated avirulence effectors of the powdery mildew pathogen. *PNAS* 113, E6486–E6495. doi:10.1073/pnas.1612947113.

Ma, Z., Song, T., Zhu, L., Ye, W., Wang, Y., Shao, Y., et al. (2015). A *Phytophthora sojae* glycoside hydrolase 12 protein is a major virulence factor during soybean infection and is recognized as a PAMP. *Plant Cell* 27, 2057–2072. doi:10.1105/tpc.15.00390.

Ma, Z., Zhu, L., Song, T., Wang, Y., Zhang, Q., Xia, Y., et al. (2017). A paralogous decoy protects *Phytophthora sojae* apoplastic effector PsXEG1 from a host inhibitor. *Science* 355, 710–714. doi:10.1126/science.aai7919.

Macho, A. P. (2016). Subversion of plant cellular functions by bacterial type-III effectors: beyond suppression of immunity. *New Phytol.* 210, 51–57. doi:10.1111/nph.13605.

Macho, A. P., and Zipfel, C. (2015). Targeting of plant pattern recognition receptor-triggered immunity by bacterial type-III secretion system effectors. *Curr. Opin. Microbiol.* 23, 14–22. doi:10.1016/j.mib.2014.10.009.

Mackey, D., Belkhadir, Y., Alonso, J. M., Ecker, J. R., and Dangl, J. L. (2003). *Arabidopsis* RIN4 is a target of the type III virulence effector AvrRpt2 and modulates RPS2-mediated resistance. *Cell* 112, 379–389. doi:10.1016/S0092-8674(03)00040-0.

Mackey, D., Holt, B. F., Wiig, A., and Dangl, J. L. (2002). RIN4 interacts with *Pseudomonas syringae* type III effector molecules and is required for RPM1-mediated resistance in *Arabidopsis*. *Cell* 108, 743–754. doi:10.1016/S0092-8674(02)00661-X.

Maekawa, T., Kufer, T. A., and Schulze-Lefert, P. (2011). NLR functions in plant and animal immune systems: so far and yet so close. *Nat. Immunol.* 12, 818–826. doi:10.1038/ni.2083.

Mak, A. N. S., Bradley, P., Cernadas, R. A., Bogdanove, A. J., and Stoddard, B. L. (2012). The crystal structure of TAL effector PthXo1 bound to its DNA target. *Science (80-. ).* 335, 716–719. doi:10.1126/science.1216211.

Marchal, C., Zhang, J., Zhang, P., Fenwick, P., Steuernagel, B., Adamski, N. M., et al. (2018). BED-domain-containing immune receptors confer diverse resistance spectra to yellow rust. *Nat. Plants* 4, 662–668. doi:10.1038/s41477-018-0236-4.

Martin, G. B., Bogdanove, A. J., and Sessa, G. (2003). Understanding the functions of plant disease resistance proteins. *Annu. Rev. Plant Biol.* 54, 23–61. doi:10.1146/annurev.arplant.54.031902.135035.

McHale, L., Tan, X., Koehl, P., and Michelmore, R. W. (2006). Plant NBS-LRR proteins: adaptable guards. *Genome Biol.* 7, 212. doi:10.1186/gb-2006-7-4-212.

Meng, X., and Zhang, S. (2013). MAPK cascades in plant disease resistance signaling. *Annu. Rev. Phytopathol.* 51, 245–266. doi:10.1146/annurev-phyto-082712-102314.

Mentlak, T. A., Kombrink, A., Shinya, T., Ryder, L. S., Otomo, I., Saitoh, H., et al. (2012). Effector-mediated suppression of chitin-triggered immunity by *Magnaporthe oryzae* is necessary for rice blast disease. *Plant Cell* 24, 322–335. doi:10.1105/tpc.111.092957.

Mukhtar, M. S., Carvunis, A. R., Dreze, M., Epple, P., Steinbrenner, J., Moore, J., et al. (2011). Independently evolved virulence effectors converge onto hubs in a plant immune system network. *Science* 333, 596–601. doi:10.1126/science.1203659.

Navathe, S., Yadav, P. S., Chand, R., Mishra, V. K., Vasistha, N. K., Meher, P. K., et al. (2020). ToxA-TSN1 interaction for spot blotch susceptibility in Indian wheat: an example of inverse gene-for-gene relationship. *Plant Dis.* 104, 71–81. doi:10.1094/PDIS-05-19-1066-RE.

Nimchuk, Z., Marois, E., Kjemtrup, S., Leister, R. T., Katagiri, F., and Dangl, J. L. (2000). Eukaryotic fatty acylation drives plasma membrane targeting and enhances function of several type III effector proteins from *Pseudomonas syringae*. *Cell* 101, 353–363. doi:10.1016/S0092-8674(00)80846-6.

Nimchuk, Z., Rohmer, L., Chang, J. H., and Dangl, J. L. (2001). Knowing the dancer from the dance: *R*-gene products and their interactions with other proteins from host and pathogen. *Curr. Opin. Plant Biol.* 4, 288–294. doi:10.1016/S1369-5266(00)00175-8.

Niño-Liu, D. O., Ronald, P. C., and Bogdanove, A. J. (2006). *Xanthomonas oryzae* pathovars: model pathogens of a model crop. *Mol. Plant Pathol.* 7, 303–324. doi:10.1111/j.1364-3703.2006.00344.x.

Noël, L., Thieme, F., Nennstiel, D., and Bonas, U. (2001). cDNA-AFLP analysis unravels a genome-wide *hrpG*-regulon in the plant pathogen Xanthomonas campestris pv. vesicatoria. *Mol. Microbiol.* 41, 1271–1281. doi:10.1046/j.1365-2958.2001.02567.x.

Nomura, K. (2006). A bacterial virulence protein suppresses host innate immunity to cause plant disease. *Science* 313, 220–223. doi:10.1126/science.1129523.

Nowara, D., Schweizer, P., Gay, A., Lacomme, C., Shaw, J., Ridout, C., et al. (2010). HIGS: Host-induced gene silencing in the obligate biotrophic fungal pathogen *Blumeria graminis*. *Plant Cell* 22, 3130–3141. doi:10.1105/tpc.110.077040.

Nürnberger, T., and Lipka, V. (2005). Non-host resistance in plants: new insights into an old phenomenon. *Mol. Plant Pathol.* 6, 335–345. doi:10.1111/j.1364-3703.2005.00279.x.

O’Connell, R. J., Thon, M. R., Hacquard, S., Amyotte, S. G., Kleemann, J., Torres, M. F., et al. (2012). Lifestyle transitions in plant pathogenic *Colletotrichum* fungi deciphered by genome and transcriptome analyses. *Nat. Genet.* 44, 1060–1065. doi:10.1038/ng.2372.

Oh, C. S., and Beer, S. V. (2005). Molecular genetics of *Erwinia amylovora* involved in the development of fire blight. *FEMS Microbiol. Lett.* 253, 185–192. doi:10.1016/j.femsle.2005.09.051.

Orth, K. (2000). Disruption of signaling by *Yersinia* effector YopJ, a ubiquitin-like protein protease. *Science*  290, 1594–1597. doi:10.1126/science.290.5496.1594.

Orth, K. (2002). Function of the *Yersinia* effector YopJ. *Curr. Opin. Microbiol.* 5, 38–43. doi:10.1016/S1369-5274(02)00283-7.

Park, C. H., Chen, S., Shirsekar, G., Zhou, B., Khang, C. H., Songkumarn, P., et al. (2012). The *Magnaporthe* *oryzae* effector AvrPiz-t targets the RING E3 ubiquitin ligase APIP6 to suppress pathogen-associated molecular pattern-triggered immunity in rices. *Plant Cell* 24, 4748–4762. doi:10.1105/tpc.112.105429.

Paulus, J. K., Kourelis, J., Ramasubramanian, S., Homma, F., Godson, A., Hörger, A. C., et al. (2020). Extracellular proteolytic cascade in tomato activates immune protease Rcr3. *PNAS* 117, 17409–17417. doi:10.1073/pnas.1921101117.

Pedley, K. F., and Martin, G. B. (2003). Molecular basis of *Pto*-mediated resistance to bacterial speck disease in tomato. *Annu. Rev. Phytopathol.* 41, 215–243. doi:10.1146/annurev.phyto.41.121602.143032.

Perez-Quintero, A. L., and Szurek, B. (2019). A decade decoded: spies and hackers in the history of TAL effectors research. *Annu. Rev. Phytopathol.* 57, 459–481. doi:10.1146/annurev-phyto-082718-100026.

Petit-Houdenot, Y., and Fudal, I. (2017). Complex interactions between fungal avirulence genes and their corresponding plant resistance genes and consequences for disease resistance management. *Front. Plant Sci.* 8, 1072. doi:10.3389/fpls.2017.01072.

Plett, J. M., Daguerre, Y., Wittulsky, S., Vayssier̀es, A., Deveau, A., Melton, S. J., et al. (2014). Effector MiSSP7 of the mutualistic fungus *Laccaria bicolor* stabilizes the *Populus* JAZ6 protein and represses jasmonic acid (JA) responsive genes. *PNAS* 111, 8299–8304. doi:10.1073/pnas.1322671111.

Plissonneau, C., Daverdin, G., Ollivier, B., Blaise, F., Degrave, A., Fudal, I., et al. (2016). A game of hide and seek between avirulence genes *AvrLm4-7* and *AvrLm3* in *Leptosphaeria maculans*. *New Phytol.* 209, 1613–24. doi:10.1111/nph.13736.

Poueymiro, M., and Genin, S. (2009). Secreted proteins from *Ralstonia solanacearum*: a hundred tricks to kill a plant. *Curr. Opin. Microbiol.* 12, 44–52. doi:10.1016/j.mib.2008.11.008.

Qiu, J. L., Fiil, B. K., Petersen, K., Nielsen, H. B., Botanga, C. J., Thorgrimsen, S., et al. (2008). *Arabidopsis* MAP kinase 4 regulates gene expression through transcription factor release in the nucleus. *EMBO J.* 27, 2214–2221. doi:10.1038/emboj.2008.147.

Raffaele, S., and Kamoun, S. (2012). Genome evolution in filamentous plant pathogens: why bigger can be better. *Nat. Rev. Microbiol.* 10, 417–430. doi:10.1038/nrmicro2790.

Rooney, H. C. E. (2005). *Cladosporium* Avr2 inhibits tomato Rcr3 protease required for Cf-2-dependent disease resistance. *Science*  308, 1783–1786. doi:10.1126/science.1111404.

Rouxel, T., Grandaubert, J., Hane, J. K., Hoede, C., Van De Wouw, A. P., Couloux, A., et al. (2011). Effector diversification within compartments of the *Leptosphaeria maculans* genome affected by repeat-induced point mutations. *Nat. Commun.* 2, 202. doi:10.1038/ncomms1189.

Rudd, J. J., Kanyuka, K., Hassani-Pak, K., Derbyshire, M., Andongabo, A., Devonshire, J., et al. (2015). Transcriptome and metabolite profiling of the infection cycle of *Zymoseptoria tritici* on wheat reveals a biphasic interaction with plant immunity involving differential pathogen chromosomal contributions and a variation on the hemibiotrophic lifest. *Plant Physiol.* 167, 1158–1185. doi:10.1104/pp.114.255927.

Saintenac, C., Lee, W. S., Cambon, F., Rudd, J. J., King, R. C., Marande, W., et al. (2018). Wheat receptor-kinase-like protein Stb6 controls gene-for-gene resistance to fungal pathogen *Zymoseptoria tritici*. *Nat. Genet.* 50, 368–374. doi:10.1038/s41588-018-0051-x.

Salanoubat, M., Genin, S., Artiguenave, F., Gouzy, J., Mangenot, S., Arlat, M., et al. (2002). Genome sequence of the plant pathogen *Ralstonia solanacearum*. *Nature* 415, 497–502. doi:10.1038/415497a.

Sarris, P. F., Cevik, V., Dagdas, G., Jones, J. D. G., and Krasileva, K. V (2016). Comparative analysis of plant immune receptor architectures uncovers host proteins likely targeted by pathogens. *BMC Biol.* 14. doi:10.1186/s12915-016-0228-7.

Sarris, P. F., Duxbury, Z., Huh, S. U., Ma, Y., Segonzac, C., Sklenar, J., et al. (2015). A plant immune receptor detects pathogen effectors that target WRKY transcription factors. *Cell* 161, 1089–1100. doi:10.1016/j.cell.2015.04.024.

Saunders, D. G. O., Win, J., Cano, L. M., Szabo, L. J., Kamoun, S., and Raffaele, S. (2012). Using hierarchical clustering of secreted protein families to classify and rank candidate effectors of rust fungi. *PLoS One* 7. doi:10.1371/journal.pone.0029847.

Saur, I. M. L., Bauer, S., Kracher, B., Lu, X., Franzeskakis, L., Müller, M. C., et al. (2019). Multiple pairs of allelic MLA immune receptor-powdery mildew AVR a effectors argue for a direct recognition mechanism. *Elife* 8. doi:10.7554/eLife.44471.

Schornack, S., Huitema, E., Cano, L. M., Bozkurt, T. O., Oliva, R., Van Damme, M., et al. (2009). Ten things to know about oomycete effectors. *Mol. Plant Pathol.* 10, 795–803. doi:10.1111/j.1364-3703.2009.00593.x.

Schulze-Lefert, P., and Panstruga, R. (2011). A molecular evolutionary concept connecting nonhost resistance, pathogen host range, and pathogen speciation. *Trends Plant Sci.* 16, 117–125. doi:10.1016/j.tplants.2011.01.001.

Selin, C., de Kievit, T. R., Belmonte, M. F., and Fernando, W. G. D. (2016). Elucidating the role of effectors in plant-fungal interactions: progress and challenges. *Front. Microbiol.* 7, 600. doi:10.3389/fmicb.2016.00600.

Shan, L., He, P., Li, J., Heese, A., Peck, S. C., Nürnberger, T., et al. (2008). Bacterial effectors target the common signaling partner BAK1 to disrupt multiple MAMP receptor-signaling complexes and impede plant immunity. *Cell Host Microbe* 4, 17–27. doi:10.1016/j.chom.2008.05.017.

Shao, F., Golstein, C., Ade, J., Stoutemyer, M., Dixon, J. E., and Innes, R. W. (2003). Cleavage of Arabidopsis PBS1 by a bacterial type III effector. *Science* 301, 1230–1233. doi:10.1126/science.1085671.

Shen, Q.-H., Saijo, Y., Mauch, S., Biskup, C., Bieri, S., Keller, B., et al. (2007). Nuclear activity of MLA immune receptors links isolate-specific and basal disease-resistance responses. *Science* 315, 1098–1103. doi:10.1126/science.1136372.

Soyer, J. L., El Ghalid, M., Glaser, N., Ollivier, B., Linglin, J., Grandaubert, J., et al. (2014). Epigenetic control of effector gene expression in the plant pathogenic fungus *Leptosphaeria maculans*. *PLoS Genet.* 10, 1004227. doi:10.1371/journal.pgen.1004227.

Spanu, P. D. (2012). The genomics of obligate (and nonobligate) biotrophs. *Annu. Rev. Phytopathol.* 50, 91–109. doi:10.1146/annurev-phyto-081211-173024.

Spanu, P. D., Abbott, J. C., Amselem, J., Burgis, T. A., Soanes, D. M., Stüber, K., et al. (2010). Genome expansion and gene loss in powdery mildew fungi reveal tradeoffs in extreme parasitism. *Science*  330, 1543–1546. doi:10.1126/science.1194573.

Sperschneider, J., Catanzariti, A. M., Deboer, K., Petre, B., Gardiner, D. M., Singh, K. B., et al. (2017). LOCALIZER: Subcellular localization prediction of both plant and effector proteins in the plant cell. *Sci. Rep.* 7. doi:10.1038/srep44598.

Sperschneider, J., Dodds, P. N., Gardiner, D. M., Singh, K. B., and Taylor, J. M. (2018a). Improved prediction of fungal effector proteins from secretomes with EffectorP 2.0. *Mol. Plant Pathol.* 19, 2094–2110. doi:10.1111/mpp.12682.

Sperschneider, J., Dodds, P. N., Singh, K. B., and Taylor, J. M. (2018b). ApoplastP: prediction of effectors and plant proteins in the apoplast using machine learning. *New Phytol.* 217, 1764–1778. doi:10.1111/nph.14946.

Sperschneider, J., Gardiner, D. M., Dodds, P. N., Tini, F., Covarelli, L., Singh, K. B., et al. (2016). EffectorP: predicting fungal effector proteins from secretomes using machine learning. *New Phytol.* 210, 743–761. doi:10.1111/nph.13794.

Staskawicz, B. J., Mudgett, M. B., Dangl, J. L., and Galan, J. E. (2001). Common and contrasting themes of plant and animal diseases. *Science*  292, 2285–2289. doi:10.1126/science.1062013.

Stergiopoulos, I., Collemare, J., Mehrabi, R., and De Wit, P. J. G. M. (2013). Phytotoxic secondary metabolites and peptides produced by plant pathogenic Dothideomycete fungi. *FEMS Microbiol. Rev.* 37, 67–93. doi:10.1111/j.1574-6976.2012.00349.x.

Stergiopoulos, I., and de Wit, P. J. G. M. (2009). Fungal effector proteins. *Annu. Rev. Phytopathol.* 47, 233–263. doi:10.1146/annurev.phyto.112408.132637.

Steuernagel, B., Witek, K., Krattinger, S. G., Ramirez-Gonzalez, R. H., Schoonbeek, H. J., Yu, G., et al. (2020). The NLR-annotator tool enables annotation of the intracellular immune receptor repertoire. *Plant Physiol.* 183, 468–482. doi:10.1104/pp.19.01273.

Stotz, H. U., Mitrousia, G. K., de Wit, P. J. G. M., and Fitt, B. D. L. (2014). Effector-triggered defence against apoplastic fungal pathogens. *Trends Plant Sci.* 19, 491–500. doi:10.1016/j.tplants.2014.04.009.

Tang, D., Wang, G., and Zhou, J. M. (2017). Receptor kinases in plant-pathogen interactions: more than pattern recognition. *Plant Cell* 29, 618–637. doi:10.1105/tpc.16.00891.

Thieme, F., Koebnik, R., Bekel, T., Berger, C., Boch, J., Büttner, D., et al. (2005). Insights into genome plasticity and pathogenicity of the plant pathogenic bacterium *Xanthomonas campestris* pv. *vesicatoria* revealed by the complete genome sequence. *J. Bacteriol.* 187, 7254–7266. doi:10.1128/JB.187.21.7254-7266.2005.

Thomma, B. P., Nurnberger, T., and Joosten, M. H. (2011). Of PAMPs and effectors: The blurred PTI-ETI dichotomy. *Plant Cell* 23, 4–15. doi:10.1105/tpc.12.5.817.

Thordal-Christensen, H. (2020). A holistic view on plant effector-triggered immunity presented as an iceberg model. *Cell. Mol. Life Sci.* 77, 3963–3976. doi:10.1007/s00018-020-03515-w.

Tian, D., Wang, J., Zeng, X., Gu, K., Qiu, C., Yang, X., et al. (2014). The rice TAL effector-dependent resistance protein XA10 triggers cell death and calcium depletion in the endoplasmic reticulum. *Plant Cell* 26, 497–515. doi:10.1105/tpc.113.119255.

Tian, W., Hou, C., Ren, Z., Wang, C., Zhao, F., Dahlbeck, D., et al. (2019). A calmodulin-gated calcium channel links pathogen patterns to plant immunity. *Nature* 572, 131–135. doi:10.1038/s41586-019-1413-y.

Timilsina, S., Potnis, N., Newberry, E. A., Liyanapathiranage, P., Iruegas-Bocardo, F., White, F. F., et al. (2020). *Xanthomonas* diversity, virulence and plant–pathogen interactions. *Nat. Rev. Microbiol.* 18, 415–427. doi:10.1038/s41579-020-0361-8.

Toruño, T. Y., Stergiopoulos, I., and Coaker, G. (2016). Plant-pathogen effectors: cellular probes interfering with plant defenses in spatial and temporal manners. *Annu. Rev. Phytopathol.* 54, 419–441. doi:10.1146/annurev-phyto-080615-100204.

Tsiamis, G., Mansfield, J. W., Hockenhull, R., Jackson, R. W., Sesma, A., Athanassopoulos, E., et al. (2000). Cultivar-specific avirulence and virulence functions assigned to *avrPphF* in *Pseudomonas syringae* pv. *phaseolicola*, the cause of bean halo-blight disease. *EMBO J.* 19, 3204–3214. doi:10.1093/emboj/19.13.3204.

Tsuda, K., and Katagiri, F. (2010). Comparing signaling mechanisms engaged in pattern-triggered and effector-triggered immunity. *Curr. Opin. Plant Biol.* 13, 459–465. doi:10.1016/j.pbi.2010.04.006.

Tsuge, T., Harimoto, Y., Akimitsu, K., Ohtani, K., Kodama, M., Akagi, Y., et al. (2013). Host-selective toxins produced by the plant pathogenic fungus *Alternaria alternata*. *FEMS Microbiol. Rev.* 37, 44–66. doi:10.1111/j.1574-6976.2012.00350.x.

Tyler, B. M., Tripathy, S., Zhang, X., Dehal, P., Jiang, R. H. Y., Aerts, A., et al. (2006). *Phytophthora* genome sequences uncover evolutionary origins and mechanisms of pathogenesis. *Science (80-. ).* 313, 1261–1266. doi:10.1126/science.1128796.

Urban, M., Cuzick, A., Rutherford, K., Irvine, A., Pedro, H., Pant, R., et al. (2017). PHI-base: a new interface and further additions for the multi-species pathogen-host interactions database. *Nucleic Acids Res.* 45, D604–D610. doi:10.1093/nar/gkw1089.

Urban, M., Cuzick, A., Seager, J., Wood, V., Rutherford, K., Venkatesh, S. Y., et al. (2020). PHI-base: The pathogen-host interactions database. *Nucleic Acids Res.* 48, D613–D620. doi:10.1093/nar/gkz904.

Urban, M., Pant, R., Raghunath, A., Irvine, A. G., Pedro, H., and Hammond-Kosack, K. E. (2015). The pathogen-host interactions database (PHI-base): additions and future developments. *Nucleic Acids Res.* 43, D645–D655. doi:10.1093/nar/gku1165.

van’t Slot, K. A. E., and Knogge, W. (2002). A dual role for microbial pathogen-derived effector proteins in plant disease and resistance. *CRC. Crit. Rev. Plant Sci.* 21, 229–271. doi:10.1080/0735-260291044223.

van Der Hoorn, R. A. L., and Kamoun, S. (2008). From guard to decoy: a new model for perception of plant pathogen effectors. *Plant Cell* 20, 2009–2017. doi:10.1105/tpc.108.060194.

van Der Vossen, E. A. G., van Der Voort, J. N. A. M. R., Kanyuka, K., Bendahmane, A., Sandbrink, H., Baulcombe, D. C., et al. (2000). Homologues of a single resistance-gene cluster in potato confer resistance to distinct pathogens: a virus and a nematode. *Plant J.* 23, 567–576. doi:10.1046/j.1365-313X.2000.00814.x.

Velásquez, A. C., Castroverde, C. D. M., and He, S. Y. (2018). Plant–pathogen warfare under changing climate conditions. *Curr. Biol.* 28, R619–R634. doi:10.1016/j.cub.2018.03.054.

Vincent, D., Rafiqi, M., and Job, D. (2020). The multiple facets of plant–fungal interactions revealed through plant and fungal secretomics. *Front. Plant Sci.* 10. doi:10.3389/fpls.2019.01626.

Vleeshouwers, V. G. A. A., Raffaele, S., Vossen, J. H., Champouret, N., Oliva, R., Segretin, M. E., et al. (2011). Understanding and exploiting late blight resistance in the age of effectors. *Annu. Rev. Phytopathol.* 49, 507–531. doi:10.1146/annurev-phyto-072910-095326.

Vleeshouwers, V. G. A. A., Rietman, H., Krenek, P., Champouret, N., Young, C., Oh, S.-K., et al. (2008). Effector genomics accelerates discovery and functional profiling of potato disease resistance and *Phytophthora infestans* avirulence genes. *PLoS One* 3, e2875. doi:10.1371/journal.pone.0002875.

Wang, J., Hu, M., Wang, J., Qi, J., Han, Z., Wang, G., et al. (2019a). Reconstitution and structure of a plant NLR resistosome conferring immunity. *Science* 364, eaav5870. doi:10.1126/science.aav5870.

Wang, J., Wang, J., Hu, M., Wu, S., Qi, J., Wang, G., et al. (2019b). Ligand-triggered allosteric ADP release primes a plant NLR complex. *Science (80-. ).* 364, eaav5868. doi:10.1126/science.aav5868.

Wang, M., Weiberg, A., Lin, F. M., Thomma, B. P. H. J., Huang, H. Da, and Jin, H. (2016). Bidirectional cross-kingdom RNAi and fungal uptake of external RNAs confer plant protection. *Nat. Plants* 2, 16151. doi:10.1038/nplants.2016.151.

Wang, Q., Han, C., Ferreira, A. O., Yu, X., Ye, W., Tripathy, S., et al. (2011). Transcriptional programming and functional interactions within the *Phytophthora sojae* RXLR effector repertoire. *Plant Cell* 23, 2064–2086. doi:10.1105/tpc.111.086082.

Wang, W., Feng, B., Zhou, J. M., and Tang, D. (2020). Plant immune signaling: advancing on two frontiers. *J. Integr. Plant Biol.* 62, 2–24. doi:10.1111/jipb.12898.

Weiberg, A., Wang, M., Lin, F.-M., Zhao, H., Zhang, Z., Kaloshian, I., et al. (2013). Fungal small RNAs suppress plant immunity by hijacking host RNA interference pathways. *Science* 342, 118–123. doi:10.1126/science.1239705.

Weßling, R., Epple, P., Altmann, S., He, Y., Yang, L., Henz, S. R., et al. (2014). Convergent targeting of a common host protein-network by pathogen effectors from three kingdoms of life. *Cell Host Microbe* 16, 364–375. doi:10.1016/j.chom.2014.08.004.

Whisson, S. C., Boevink, P. C., Moleleki, L., Avrova, A. O., Morales, J. G., Gilroy, E. M., et al. (2007). A translocation signal for delivery of oomycete effector proteins into host plant cells. *Nature* 450, 115–118. doi:10.1038/nature06203.

Williams, S. J., Sohn, K. H., Wan, L., Bernoux, M., Sarris, P. F., Segonzac, C., et al. (2014). Structural basis for assembly and function of a heterodimeric plant immune receptor. *Science* 344, 299–303. doi:10.1126/science.1247357.

Winstanley, C., and Hart, C. A. (2001). Type III secretion systems and pathogenicity islands. *J. Med. Microbiol.* 50, 116–126. doi:10.1099/0022-1317-50-2-116.

Wu, L., Chen, H., Curtis, C., and Fu, Z. Q. (2014). Go in for the kill: how plants deploy effector-triggered immunity to combat pathogens. *Virulence* 5, 710–721. doi:10.4161/viru.29755.

Wyatt, N. A., Richards, J. K., Brueggeman, R. S., and Friesen, T. L. (2020). A comparative genomic analysis of the barley pathogen *Pyrenophora* *teres* f. *teres* identifies subtelomeric regions as drivers of virulence. *Mol. Plant-Microbe Interact.* 33, 173–188. doi:10.1094/MPMI-05-19-0128-R.

Xia, Y. (2004). Proteases in pathogenesis and plant defence. *Cell. Microbiol.* 6, 905–913. doi:10.1111/j.1462-5822.2004.00438.x.

Xiang, T., Zong, N., Zou, Y., Wu, Y., Zhang, J., Xing, W., et al. (2008). *Pseudomonas syringae* effector AvrPto blocks innate immunity by targeting receptor kinases. *Curr. Biol.* 18, 74–80. doi:10.1016/j.cub.2007.12.020.

Xin, X.-F., Nomura, K., Aung, K., Velásquez, A. C., Yao, J., Boutrot, F., et al. (2016). Bacteria establish an aqueous living space in plants crucial for virulence. *Nature* 539, 524–529. doi:10.1038/nature20166.

Xin, X. F., and He, S. Y. (2013). *Pseudomonas syringae* pv. *tomato* DC3000: a model pathogen for probing disease susceptibility and hormone signaling in plants. *Annu. Rev. Phytopathol.* 51, 473–498. doi:10.1146/annurev-phyto-082712-102321.

Xin, X. F., Kvitko, B., and He, S. Y. (2018). *Pseudomonas syringae*: what it takes to be a pathogen. *Nat. Rev. Microbiol.* 16, 316–328. doi:10.1038/nrmicro.2018.17.

Yang, B., Sugio, A., and White, F. F. (2006). *Os8N3* is a host disease-susceptibility gene for bacterial blight of rice. *PNAS* 103, 10503–10508. doi:10.1073/pnas.0604088103.

Yang, J., Duan, G., Li, C., Liu, L., Han, G., Zhang, Y., et al. (2019). The crosstalks between jasmonic acid and other plant hormone signaling highlight the involvement of jasmonic acid as a core component in plant response to biotic and abiotic stresses. *Front. Plant Sci.* 10, 1349. doi:10.3389/fpls.2019.01349.

Yoshida, K., Saitoh, H., Fujisawa, S., Kanzaki, H., Matsumura, H., Yoshida, K., et al. (2009). Association genetics reveals three novel avirulence genes from the rice blast fungal pathogen *Magnaporthe oryzae*. *Plant Cell* 21, 1573–1591. doi:10.1105/tpc.109.066324.

Zhang, J., Li, W., Xiang, T., Liu, Z., Laluk, K., Ding, X., et al. (2010). Receptor-like cytoplasmic kinases integrate signaling from multiple plant immune receptors and are targeted by a *Pseudomonas syringae* effector. *Cell Host Microbe* 7, 290–301. doi:10.1016/j.chom.2010.03.007.

Zhang, J., Shao, F., Li, Y., Cui, H., Chen, L., Li, H., et al. (2007). A *Pseudomonas syringae* effector inactivates MAPKs to suppress PAMP-induced immunity in plants. *Cell Host Microbe* 1, 175–185. doi:10.1016/j.chom.2007.03.006.

Zhang, Y., and Li, X. (2019). Salicylic acid: biosynthesis, perception, and contributions to plant immunity. *Curr. Opin. Plant Biol.* 50, 29–36. doi:10.1016/j.pbi.2019.02.004.

Zhong, Z., Marcel, T. C., Hartmann, F. E., Ma, X., Plissonneau, C., Zala, M., et al. (2017). A small secreted protein in *Zymoseptoria tritici* is responsible for avirulence on wheat cultivars carrying the *Stb6* resistance gene. *New Phytol.* 214, 619–631. doi:10.1111/nph.14434.

Zipfel, C. (2009). Early molecular events in PAMP-triggered immunity. *Curr. Opin. Plant Biol.* 12, 414–420. doi:10.1016/j.pbi.2009.06.003.

Zvereva, A. S., and Pooggin, M. M. (2012). Silencing and innate immunity in plant defense against viral and non-viral pathogens. *Viruses* 4, 2578–2597. doi:10.3390/v4112578.
